# The telomere-to-telomere (T2T) genome of *Peucedanum praeruptorum* Dunn provides insights into the genome evolution and coumarin biosynthesis

**DOI:** 10.1093/gigascience/giae025

**Published:** 2024-06-05

**Authors:** Mingzhou Bai, Sanjie Jiang, Shanshan Chu, Yangyang Yu, Dai Shan, Chun Liu, Liang Zong, Qun Liu, Nana Liu, Weisong Xu, Zhanlong Mei, Jianbo Jian, Chi Zhang, Shancen Zhao, Tsan-Yu Chiu, Henrik Toft Simonsen

**Affiliations:** DTU Bioengineering, Technical University of Denmark, Kongens Lyngby 2800, Denmark; BGI-Genomics, BGI-Shenzhen, Shenzhen 518000, China; BGI-Genomics, BGI-Shenzhen, Shenzhen 518000, China; School of Pharmacy, Anhui University of Chinese Medicine, Hefei 230000, China; Anhui Province Key Laboratory of Research and Development of Chinese Medicine, Hefei 230000, China; BGI-Genomics, BGI-Shenzhen, Shenzhen 518000, China; BGI-Genomics, BGI-Shenzhen, Shenzhen 518000, China; College of Tropical Crops, Hainan University, Haikou 570228, China; Wuhan BGI Technology Service Co., Ltd. BGI-Wuhan, Wuhan 430000, China; Wuhan BGI Technology Service Co., Ltd. BGI-Wuhan, Wuhan 430000, China; College of Pharmaceutical Science, Zhejiang University of Technology, Hangzhou 310000, China; HIM-BGI Omics Center, Zhejiang Cancer Hospital, Hangzhou Institute of Medicine (HIM), Chinese Academy of Sciences (CAS), Hangzhou 310000, China; BGI-Genomics, BGI-Shenzhen, Shenzhen 518000, China; BGI-Genomics, BGI-Shenzhen, Shenzhen 518000, China; DTU Bioengineering, Technical University of Denmark, Kongens Lyngby 2800, Denmark; BGI-Genomics, BGI-Shenzhen, Shenzhen 518000, China; BGI-Genomics, BGI-Shenzhen, Shenzhen 518000, China; BGI-Genomics, BGI-Shenzhen, Shenzhen 518000, China; BGI-Genomics, BGI-Shenzhen, Shenzhen 518000, China; HIM-BGI Omics Center, Zhejiang Cancer Hospital, Hangzhou Institute of Medicine (HIM), Chinese Academy of Sciences (CAS), Hangzhou 310000, China; Laboratoire Biotechnologies Végétales Plantes aromatiques et médicinales, Université Jean Monnet, St. Étienne 42023, France

**Keywords:** *Peucedanum praeruptorum*, T2T genome, coumarin biosynthesis

## Abstract

**Background:**

Traditional Chinese medicine has used *Peucedanum praeruptorum* Dunn (Apiaceae) for a long time. Various coumarins, including the significant constituents praeruptorin (A–E), are the active constituents in the dried roots of *P. praeruptorum*. Previous transcriptomic and metabolomic studies have attempted to elucidate the distribution and biosynthetic network of these medicinal-valuable compounds. However, the lack of a high-quality reference genome impedes an in-depth understanding of genetic traits and thus the development of better breeding strategies.

**Results:**

A telomere-to-telomere (T2T) genome was assembled for *P. praeruptorum* by combining PacBio HiFi, ONT ultra-long, and Hi-C data. The final genome assembly was approximately 1.798 Gb, assigned to 11 chromosomes with genome completeness >98%. Comparative genomic analysis suggested that *P. praeruptorum* experienced 2 whole-genome duplication events. By the transcriptomic and metabolomic analysis of the coumarin metabolic pathway, we presented coumarins’ spatial and temporal distribution and the expression patterns of critical genes for its biosynthesis. Notably, the *COSY* and cytochrome *P450* genes showed tandem duplications on several chromosomes, which may be responsible for the high accumulation of coumarins.

**Conclusions:**

A T2T genome for *P. praeruptorum* was obtained, providing molecular insights into the chromosomal distribution of the coumarin biosynthetic genes. This high-quality genome is an essential resource for designing engineering strategies for improving the production of these valuable compounds.

## Background

*Peucedanum praeruptorum* Dunn (NCBI:txid312531) belongs to the plant family Apiaceae. Its dried root “Peucedani Radix” is used in traditional Chinese medicine, and the pharmacological activity is attributed to terpenoids and coumarins (e.g., praeruptorin A–E) [1]. The root extracts of *P. praeruptorum* have been applied to treat headaches, coughing, and vomiting and have the potential to reverse multidrug resistances [1]. Among the active ingredients, coumarins are a class of compounds with a core structure that comprise a fused benzene and α-pyrone ring. Generally, coumarins can be classified as simple coumarins, furocoumarins, pyranocoumarins, phenylcoumarins, and biscoumarins [2, 3]. Simple coumarins are widespread in various plant families, but the distribution of furanocoumarins is more limited. Furanocoumarins are identified in some plant families, including Apiaceae, Asteraceae, Moraceae, Pittosporaceae, Rosaceae, Rutaceae, Solanaceae, and Thymelaeaceae [4]. Linear furanocoumarins have been found in at least 19 plant families, with the majority found in Rutaceae and Apiaceae. The Apiaceae family, especially in the subfamily Apioideae, is a notable source of both linear and angular furanocoumarins [3, 4]. While many plants synthesize linear furanocoumarins without angular counterparts, the production of angular furanocoumarins without linear ones is rare, suggesting a more recent evolution of angular biosynthesis [2, 3]. However, no genetic evidence has supported this hypothesis yet.

The biosynthesis of the coumarin core structure is derived from phenylalanine. The phenylalanine is deaminated by phenylalanine ammonia lyase (PAL) to cinnamic acid and sequentially metabolized by cinnamate 4-hydroxylase (C4′H), 4-coumarate-coenzyme A (CoA) ligase (4′CL), and *p*-coumaroyl-CoA 2′-hydroxylase (C2′H) to form umbelliferone [5, 6]. Many feeding studies have shown that umbelliferone is the precursor to form more complex coumarins (e.g., pyranocoumarins or furanocoumarins) [7].

The umbelliferone dimethylallyltransferases (UDTs) are enzymes belonging to the prenyltransferase family. The UDTs perform specific prenylation at either the C6 or C8 position of umbelliferone, which then lead to linear or angular furano/pyranocoumarins, respectively [8]. Currently, through the analysis of both transcriptomic and metabolomic data, 3 distinct prenyltransferases (PpPT1–3) in *P. praeruptorum* have been identified as responsible for the prenylation of the simple coumarin skeleton, forming linear or angular precursors. Additionally, 2 novel CYP450 cyclases (PpDC and PpOC) have been shown to be responsible for the cyclization of these linear/angular precursors into either tetrahydrofurans or tetrahydropyrans [9]. Another recent study in *P. praeruptorum* also combined comparative transcriptomics and metabolomics to provide insights into transcriptional changes and the reduction of coumarins after blooming, disclosing the key gene regulatory networks of coumarin biosynthesis in *P. praeruptorum* at the vegetative growth stages and reproductive stages [10].

Comparative genomic analysis across species provides insights into understanding evolutionary relationships and the genetic basis of speciation. Several high-quality genomes in Apioideae have been published, including carrot [11], coriander [12], celery [13, 14], and medicinal plants such as *Angelica sinensis* [15, 16] and *Bupleurum chinense* [17]. A chromosomal-level genome of *P. praeruptorum* was very recently published [18]. Here, we independently assembled a telomere-to-telomere (T2T) genome of *P. praeruptorum* along with identifications of genes that coded for enzymes that are involved in the biosynthesis of the medically important coumarins. The genetic basis of these key traits in *P. praeruptorum* can provide a clear roadmap (e.g., gene clusters, regulatory elements) for future breeding or even the key biosynthetic gene discoveries for synthetic biology applications.

## Methods

### Plant materials and DNA/RNA isolation

The individual plants of *P. praeruptorum* Dunn (Apiaceae) were collected between April 2022 and November 2022 at the Anhui University of Chinese Medicine Garden in Heifei City, Anhui Province, China. The plant growth site was situated amid the Huai and Yangtze Rivers, commonly called the Jianghuai area. Fresh, young, and healthy leaves were harvested for the extraction of high-molecular-weight genomic DNA using a modified cetyltri-methylammonium bromide (CTAB) method and nuclei method, respectively, for short reads and long reads (PacBio and Nanopore ultra-long) sequencing. Samples from leaves, stems, roots, flowers, and fruit tissues at 3 different growth stages were utilized for RNA extraction employing a RNeasy PowerWater Kit (Qiagen).

### Library preparation and sequencing

The quality control and quantity assessment of the isolated DNA was conducted using a NanoDrop 2000 (Thermo Scientific) and a Qubit 2.0 Fluorometer (Life Technologies). Following purification with the Qiagen genomic kit (Qiagen, 13343), approximately 5 μg *P. praeruptorum* DNA was utilized for constructing short DNA insert size (∼350 bp) libraries using the MGIEasy Universal DNA Library Prep Kit and generating 20-kb PacBio HiFi sequencing libraries with the SMRTbell Prep Kit 2.0. Subsequently, short libraries were sequenced on an DNBSEQ-T7 (RRID:SCR_017981) sequencing platform with 150-bp paired-end reads. The SageHLS HMW library system (Sage Science) was utilized to select approximately 10 μg genomic DNA with a size of about 100 kb for the construction of an ultra-long Nanopore library using the ONT 1D Sequencing Kit (SQK-LSK109). PacBio HiFi sequencing and ultra-long ONT libraries were performed on the PacBio SequeII platform (RRID:SCR_017990) and Nanopore PromethION sequence (RRID:SCR_017987). SMRT cell subread was generated and processed using the CCS algorithm of SMRTLink (v8.0.0) [19, 20]. The MGIEasy RNA Directional Library Prep Kit (MGI) was utilized to construct RNA libraries, with approximately 1 to 2 μg total RNA from each tissue sample employed. Subsequently, all libraries were subjected to sequencing on DNBSEQ-G400 (RRID:SCR_017980), generating 150-bp paired-end reads.

The Hi-C library was prepared to facilitate the anchoring of assembled contigs to chromosomes through the following steps. The fresh young leaves were cross-linked using formaldehyde (Sigma), followed by resuspension in lysis buffer. Chromatins were fragmented using MboI (NEB) restriction endonucleases. Biotin labeling was performed, and cross-linking was achieved using T4 DNA Ligase (ENZYMATICS). The captured fragments were isolated using Streptavidin-coated magnetic beads (Thermo Fisher Scientific). An “A” base was added at the 3′-end of each strand using the KAPA HYPER PREP KIT (KAPA). After purification, the Hi-C library was sequenced with PE150 in the DNBSEQ-T7 sequencing platform.

### Genome survey and *de novo* assembly

A pilot genome survey was performed prior to the long read (PacBio and ONT) sequencing. This was done to establish a cost-effective strategy. With the 150-bp short reads, Jellyfish [21] and Genomescope (RRID:SCR_017014) 1.0 were used to predict the genomic characteristics [22]. The genome size and heterozygosity rate of the *P. praeruptorum* were determined through *k*-mer analysis. This showed that PacBio, Hi-C, and ONT could be done on the extracted DNA.

With the combination of PacBio HiFi, Hi-C, and ONT ultra-long data (with a length of 100 kb or more), an initial *P. praeruptorum* contig assembly was performed by Hifiasm (RRID:SCR_021069) (version 0.19.5) with default parameters to obtain the draft genome [23].

Purge_haplotigs (RRID:SCR_017616) (version 1.0.4, parameter: -a 70) [24] was applied to identify possible hybrid sequences in the draft genome based on sequence similarity and read coverage depth, and such hybrid sequences were removed prior to genome anchoring according to the interaction and depth conditions. To identify possible contamination and plastid (chloroplasts and mitochondrial) sequences, BLASTN (RRID:SCR_001598) (version 2.11.0+, parameters: -evalue 0.00001 -max_hsps 1) was used to perform NT alignment on the draft genome to identify such sequences. These were removed prior to the genome anchoring.

Following the “cleanup” of the sequence data, Juicer (RRID:SCR_017226) (version 1.6, parameter: default) [20] was used to align the Hi-C data to the draft genome, and 3D-DNA (version 180922, parameter: -r 0) [25] was used for preliminary anchoring. JuiceBox (RRID:SCR_021172) (version 1.11.08) [26] was applied to visualize the 3D-DNA results for manual error correction, generating a “chromosome genome.” The ONT ultra-long reads were aligned to the “chromosome genome” sequences using minimap2 (version 2–2.24) [27], and the gap filing was facilitated by TGS-GapCloser (RRID:SCR_017633) (version v1.2.0, parameter: –min_nread 10) [28]. All reads aligned once within 100 bp at the end of the chromosome were collected, and the reads containing artifact sequences were filtered out. The read with the median extendable length was defined as ref and the others as a query. Medaka_consensus (version 1.7.2, parameter: -ax map-ont) [29] was applied to reassemble the ref telomere and the query telomere to get the consensus sequences.

The consensus sequences (more than 4 repeat units) were aligned to both ends of each chromosome by BLASTN (version 2.11.0+) [30] according to the positional relationship in the alignment. The telomere sequence was replaced with the aligned sequences at coverage ≥90. The gap-free genome sequence was obtained, and error correction was performed on short reads using Pilon (RRID:SCR_014731) (version 1.23, parameters: –fix snps, indels) [31]. The distribution of all repeat categories was investigated. The LINE/L1 distribution is consistent with the centromere distribution of *P. praeruptorum*, and this region is also located in a low-gene region. The completeness of the new genome was assessed using BUSCO (RRID:SCR_015008) (version 5.1.2) with the embryophyta_odb10 database, which comprises 1,614 conserved core eukaryotic genes [32].

### Genome annotation

The newly gap-free assembled genomes of *P. praeruptorum* were utilized to annotate repetitive elements and genes. The annotation of repetitive sequences is performed using a combination of methods. First, *de novo* prediction based on features of repeated sequences utilizing TRF (version 4.09) was performed [33]. Second, a homology-based prediction method employing RepeatMasker (RRID:SCR_012954) (version open-4.0.9) was utilized [34] based on a repeat database [35], followed by the construction of a custom library for repetitive sequence features using RepeatModeler (RRID:SCR_015027) (version open-1.0.11v2.0) [36] and LTR_FINDER (version 1.0.7) [37]. *De novo* predictions were performed through RepeatMasker (version open-4.0.9) [34].

The prediction of the gene set was conducted by integrating different methods, including homologous prediction based on homologs from 9 closely related species (*Angelica sinensis, Apium graveolens, Aralia elata, Coriandrum sativum, Daucus carota, Eleutherococcus senticosus, Oenanthe sinensis, Panax ginseng, Panax notoginseng*), using Exonerate (RRID:SCR_016088) (version 2.2.0) [38] and Liftoff (version 1.6.3) [39]. *De novo* prediction based on *ab initio* approaches was also employed, including AUGUSTUS (RRID:SCR_008417) (version v3.2.3) [40] and GlimmerHMM (RRID:SCR_008417) (version 3.0.4) [41], and the transcriptome-based prediction was performed using RNA sequencing (RNA-seq) data. A total of 198 Gb of the 33 samples of newly sequenced RNA-seq data were mapped to the newly assembled genome sequences using HISAT2 (RRID:SCR_015530) (version 2.1.0) [42]. Stringtie (RRID:SCR_016323) 2.1.6 [43] was employed for transcript identification and transcript-assisted annotation. Finally, the gene set of *P. praeruptorum* was integrated from the different methods by implementing the MAKER pipeline (RRID:SCR_005309) (v3.31.8) [44]. For noncoding RNA, tRNAscan-SE (RRID:SCR_008637) (version 1.3.1) [45] was used to identify transfer RNA (tRNA) sequences in the genome based on the structural characteristics of tRNA. Since ribosomal RNA (rRNA) is highly conserved, rRNA sequences of the closely related species mentioned above were selected as reference sequences, and BLASTN alignment was used to identify rRNA. MicroRNA (miRNA) and small nuclear RNA (snRNA) sequences were annotated by the covariance model of the Rfam family and INFERNAL that comes with Rfam (RRID:SCR_007891) (version 14.8) [46]. The completeness of the genome annotation was assessed using BUSCO (version 5.1.2) [32] with the embryophyta_odb10 database.

### Gene family and phylogenomic analysis

The genome sequences of *P. praeruptorum* and 10 representative plants (*A. sinensi*s, *A. graveolens, Arabidopsis thaliana, C. sativum, D. carota, Oryza sativa, P. notoginseng, Populus trichocarpa, Theobroma cacao, Vitis vinifera*) were utilized for gene family clustering and phylogenetic analysis. The gene sets of the 11 species included in the analysis were processed as follows. In cases where multiple transcripts of a gene (resulting from variable splicing) existed in the annotation files, only the longest transcript was retained. Genes encoding proteins with fewer than 30 amino acids or genes containing internal stop codons were excluded. The protein sequence similarity among all species was determined using an all-vs-all BLASTP (RRID:SCR_001010) (e-value 1e-5) approach, followed by gene family clustering using OrthoMCL (RRID:SCR_007839) (version 2.0.9) [47]. A total of 489 single-copy orthologous genes were identified, and multisequence alignment of coding sequences was aligned using MAFFT (RRID:SCR_011811) (version 7.487) [48]. This was followed by a single-copy supergene filtered with a minimum corresponding amino acid length of 100. The conserved sites were obtained using the default parameters of Gblocks (RRID:SCR_015945) (version 0.91b) [49], followed by the construction of a phylogenetic tree using RaxML (RRID:SCR_006086) (version 8.2.12) [50] with parameter (-fa -N 100 -m GTRGAMMA). The divergence time of 11 target plant species was inferred using MCMCtree of PAML (RRID:SCR_014932) (version 4.9j) [51], incorporating 2 calibrated divergence time intervals: *O. sativa–V. vinifera* (163.5–142.1 million years ago [Mya]) and *P. notoginseng–D. carota* (69.0–54.3 Mya) by TimeTree (RRID:SCR_021162). The gene family expansion and contraction of 11 species were identified using the CAFE (version 4.2) pipeline [41], and the gene families were subjected to KEGG and GO enrichment analysis to elucidate their functional roles. The collinearity of genome and whole-genome duplication (WGD) events was analyzed by the WGDI pipeline [52]. First, the protein sequences of *P. praeruptorum* were compared to those of 3 other species (*A. sinensis, C. sativum*, and *D. carota*) through all-vs-all BLASTP analysis with an e-value threshold set at 1e-5. Subsequently, gene location information and chromosome length data were extracted. The syntenic blocks’ synonymous substitution rate (Ks) were used to plot the dot.

### RNA-seq data analysis

**Figure 1:**
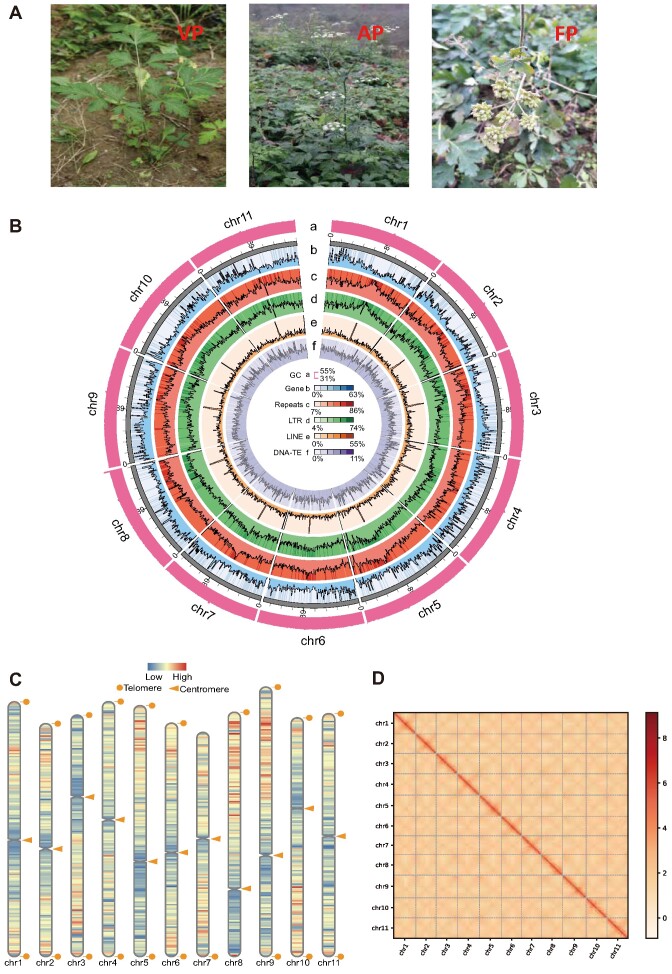
Overview of the *P. praeruptorum* and its T2T genome. (A) The morphological characteristics of *P. praeruptorum* in 3 developmental stages: VP (vegetative period), AP (anthesis period), and FP (fruit period). (B) The circos plot from the outer to the inner circle represents 11 T2T chromosomes (Chr01–Chr11). The distribution of genome features within 3-Mb windows is presented: a, GC contents; b, gene density; c, repeats density; d, LTR density; e, LINE density; f, DNA-TE density. (C) The identification of telomeres and centromeres of the 11 chromosomes. The orange circles represent telomeres on the assembled chromosomes. The high gene densities are displayed in red, and the low gene densities are displayed in blue. (D) Hi-C heatmap demonstrates the interactions between 11 chromosomes.

The experiment involved a total of 33 samples, including 3 different tissue types at different growth stages: vegetative growth stage (VP)—root, stem, leaf; flowering stage (AP)—root, stem, leaf, flower; and fruiting stage (FP)—root, stem, leaf, fruit (Fig. 1). Three biological replicates represented each tissue type. The low-quality raw reads of each sample were processed first using SOAPnuke (RRID:SCR_015025) (version 1.5.2) [53]. The clean reads were aligned to the newly assembled reference genome and gene sequence using HISAT (RRID:SCR_015530) (version 2.1.0) [42] and Bowtie2 (RRID:SCR_016368) (version 2.4.5) [54], respectively. The gene and transcript expression levels were quantified using RSEM (RRID:SCR_000262) (version 1.2.8) [55]. Differentially expressed genes (DEGs) were calculated by DESeq2 (RRID:SCR_015687) [56]. The coexpression network was constructed using the WGCNA (RRID:SCR_003302) (version 1.71) package in R [57]. The phenotypic data (the value of specialized metabolites in each tissue) were utilized and imported into the WGCNA framework, enabling the calculation of correlation-based associations between them. Subsequently, the adjacency matrix was transformed into a topological overlap matrix using WGCNA. The node and edge datasets were imported into cytoscape (RRID:SCR_003032) (version 3.10.0) [58] for the final figure. The genes (e.g., phenylalanine ammonia-lyase [PAL], cinnamate 4-hydroxylase [C4H], cinnamate 3-hydroxylase [C3H], CoA O-methyltransferase [COMT], 4-coumarate-CoA ligase [4CL], *p*-coumaroyl CoA 2′-hydroxylase [C2′H], coumarin synthase [COSY], shikimate hydroxycinnamoyl transferase [HCT], *p*-coumaroyl 5-O-quinate/shikimate 3′-hydroxylase [C3′H], caffeoyl-CoA O-methyltransferase [CCoAOMT], feruloyl-CoA 6′-hydroxylase [F6′H], glucose 6-phosphate [U-6-P], and glucose 6-phosphate [U-8-P]) involved in the coumarin biosynthesis in *P. praeruptorum* were identified by using the genes from *A. sinensis, A. thaliana, Zingiber officinale*, and *P. sativa* as a query. The genes with identity ≥80 and coverage ≥70% were selected. The heatmap of their expression patterns was displayed by pheatmap (RRID:SCR_016418) (version 1.0.8).

### Quantitative PCR

Quantitative PCR (qPCR) was employed to assess messenger RNA (mRNA) transcription levels for PpPT1/PpPT2/PpOC/PpDC. The assay utilized ensured unbiased amplification of the prevalent alleles at each locus, excluding amplification of all other loci. The primers used for amplification are listed in Supplementary Table 23. Reverse transcription was conducted using the HiScript Ill All-in-one RT SuperMix Perfect for qPCR (R333-01) from Vazyme. Amplification of candidate genes and the reference gene SAND complementary DNA (cDNA) was carried out using Pro Universal SYBR gPCR Master Mix (Vazyme) on a QuantStudio 3 Real-Time PCR System (Thermo Fisher Scientific). The average expression level of each gene was normalized to that of SAND and calculated using the 2^−∆∆Ct^ method, where Ct represents the threshold cycle [59]. In correlation analysis between the log_2_ fold change (log_2_FC) (RNA-seq) and log_2_FC (qPCR), the linear regression *r*^2^ (goodness of fit) is reported.

### Metabolic analysis

A total of 66 tissues were collected for metabolic analysis. Per the transcriptome samples, each tissue was represented by 6 biological replicates. The metabolite profiling from each tissue was performed using a nontargeted metabolomics approach, following the established protocol by Tohge and Fernie [60]. High-resolution mass spectrometry (HRMS) was conducted using an ultraperformance liquid chromatography (UPLC) system followed by the Q Exactive™ Plus Hybrid Quadrupole-Orbitrap™ mass spectrometer (MS). The base peak chromatogram (BPC) was utilized to represent a continuous depiction of the highest ion intensities recorded at each time point. All quality control samples were superimposed in positive and negative ion modes, demonstrating excellent stability and high-quality data obtained from the instrument detection process. The differential metabolites between the 2 biological groups were screened using univariate and multivariate analyses with a VIP (the Variable Importance in the Projection) ≧1, fold change ≧1.2 or ≦0.83, and *q* value <0.05.

### Analysis of coumarin biosynthesis-related cytochrome P450 gene family

The coumarin biosynthesis-related cytochrome P450 gene family with CYP71A and CYP82C was used as a query to blast the *P. praeruptorum* genomes with a similarity of 70% as the cutoff. A phylogenetic tree of these members was constructed using the adjacency method of RAxML (version 8.2.0) [50]. The chromosome location of these identified cytochrome P450 genes was labeled, and the gene structure was visualized using TBtools (RRID:SCR_023018) (version 2.019) [61].

MEME (RRID:SCR_001783) (version 5.5.5) was used to predict the motifs of these CYP genes, and the number was set to 10 [62]. The promoter region of each CYP gene with a length of 2,000 bp was obtained from the genome, and *cis*-acting regulatory elements were predicted according to the PlantCARE database [63]. The predicted *cis*-elements were divided into 7 functional categories: common, light, hormone, stress, development, other, and flavonoid biosynthesis. A statistical histogram of the number of *cis*-elements was generated.

## Results

### Genome assembly and genome annotation

The preliminary genomic information of *P. praeruptorum* (Fig. 1A) estimated a genome size of 1.78 Gb, and the heterozygosity rate was calculated to be 1.3% (Supplementary Fig. S1 and Supplementary Table S1). Further, we applied PacBio HiFi sequencing (72.6 Gb), ONT ultra-long DNA sequencing (349.7 Gb), and Hi-C sequencing (533 Gb) to yield highly accurate long-read sequencing datasets (Supplementary Table S2). After initial assembly by hifiasm, a 1.86-Gb size draft genome was generated with a GC content of approximately 35.5% (Supplementary Fig. S2, Supplementary Table S3). A chromosome-level genome assembly (hereafter named “chromosome genome”) with Hi-C alignment yielded a 1.8-Gb genome with contig N50 148.7 Mb. Then, the ONT ultra-long sequences were used to fill the gaps of the “chromosome genome,” and thereby a telomere-to-telomere level genome was generated consisting of 11 chromosomes (Fig. 1B and C). This nearly gapless genome consists of 253 contigs, N50 of 161 Mb, and a GC content of 35.5% (Supplementary Table S3). The telomeres and centromeres were identified and characterized (Supplementary Tables S4 and S5). The final HiC-heatmap of this T2T genome is presented in Fig. 1D. The short-read and long-read data were mapped to the newly assembled sequences using BWA [64] and minimap2 [27]; this allowed evaluation of the accuracy of assembly sequences. Respectively, the 99.73% mapping rate and 99.91% coverage rate with depth >4 showed a high consistency between assembly results and reads; it also plotted the GC content and depth distribution for analyzing the sequencing uniformity (Supplementary Fig. S3). Furthermore, the BUSCO analysis showed that the assembled genomes exhibit a completeness of more than 98.2% identified in the “eukaryote_odb10” database (Supplementary Table S6).

A total of 1.07 Gb of repeat sequences were detected, accounting for 59.67% of the assembled genome (Supplementary Table S7). This repeat content was less than the value (79.3%) predicted by the *k*-mer analysis (Supplementary Table S1). The most abundant transposable elements were long terminal repeats (LTRs), which account for 49.02% of the genome (Supplementary Table S8). A total of 247,398 and 164,100 protein-coding genes were *de novo* predicted using GlimmerHMM and AUGUSTUS, respectively (Supplementary Table S9). Nine well-assembled plant genomes in Apiales, including the Apiaceae species *A. sinensis, A. graveolens, C. sativum*, and *D. carota*, and *O. sinensis*, and the Araliaceae species *A. elata, E. senticosus, P. ginseng*, and *P. notoginseng*, were used for homologous prediction. The predicted genes were integrated into a nonredundant, more complete gene set with 53,756 protein-coding genes by MAKER2 [44] (Supplementary Figs. S4–S6). A final reliable set of 44,468 high-confidence genes was obtained using the in-house script (Supplementary Tables S9 and S10). The gene function of the protein-coding genes was defined by the following databases: NR (94.18%), SwissProt (56.85%), TrEMBL (93.83%), KOG (65.75%), TF (5.96%), InterPro (77.48%), GO (57.43%), KEGG_ALL (85.47%), KEGG_KO (32.73%), and Pfam (69.61%) (Supplementary Figs. S7–S9). A total of 95.4% of protein-coding genes were annotated (Supplementary Table S11). We also annotated the noncoding RNAs and acquired 181 miRNAs, 2,359 tRNAs, 6,879 rRNAs, and 8,823 snRNAs (Supplementary Table S12). Furthermore, BUSCO analysis showed that the annotated genes exhibit 97.3% completeness in the database (Supplementary Table S6). Compared with the newly published *P. praeruptorum* genome [18], the data from this study demonstrated high quality in terms of assembly and annotation (Supplementary Tables S13 and S14, Supplementary Fig. S10).

### Evolutionary analysis

**Figure 2:**
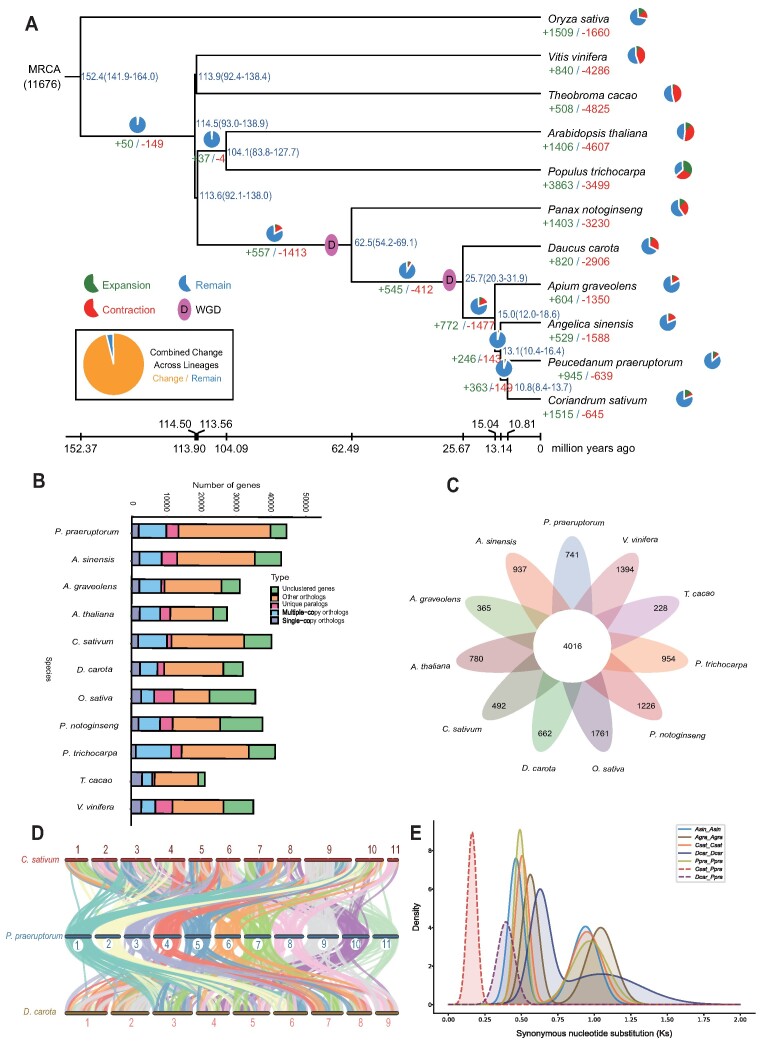
Comparative genomic analysis of the *P. praeruptorum* T2T genome. (A) The estimation of divergence time and gene family expansion/contraction. The numbers next to each branch node represent the estimated divergence time (million years ago, Mya), with the confidence range in brackets. The pie chart demonstrates the ratio of gene families with expansion (green), contraction (red), and stability (blue). (B) Number of homologous genes shared by different species. (C) The gene family clustering is demonstrated by the Petal Map. The middle circle is the number of gene families common to all species, and the edge is the number of gene families unique to each species. (D) The chromosomal collinearity among *C. sativum, P. praeruptorum*, and *D. carota*. (E) The Ks distribution map within and between species

A phylogenetic tree was constructed to estimate the divergence time of *P. praeruptorum* and 10 other representative plant species (Fig. 2A and Supplementary Fig. S11). *P. praeruptorum* belongs to the order of Apiales; it diverged from the other plant orders approximately 113.6 Mya. Within the Apiales, *P. praeruptorum* clustered with its relatives in the Apiaceae family, which diverged from the Araliaceae family member *P. notoginseng* about 62.5 Mya (Fig. 2A). In total, 725 gene family contractions and 913 gene family expansions were detected in *P. praeruptorum*. Functional enrichment analysis was performed for those expansion and contraction genes (Supplementary Figs. S12–S15). The genome of an organism is a dynamic landscape, shaped by evolutionary forces and environmental pressures. The expansion of these gene families contributes to the resilience and adaptability of organisms, allowing them to exploit diverse ecological niches. The top 3 expanded gene families were enriched in the GO terms of monooxygenase activity, oxidoreductase activity, and iron ion binding, respectively (Supplementary Fig. S14), and in the KEGG pathways of photosynthesis, spliceosome, and protein processing in endoplasmic reticulum (Supplementary Fig. S15). It is worth mentioning that the secretion of coumarins, phenolic secondary metabolites deriving from the general phenylpropanoid pathway, is a common approach induced by iron starvation and is thought to mobilize the recalcitrant iron pools. Interestingly, the expansion of gene families associated with monooxygenase activity, oxidoreductase activity, and iron ion binding signifies an enhanced capacity for *P. praeruptorum* to synthesize diverse coumarins, engage in crucial redox reactions, and efficiently manage iron acquisition in nature. In contrast, the top 2 contracted gene families were enriched in the GO terms of pathways of intramolecular transferase activity and hydrolase activity, as well as hydrolyzing O-glycosyl compounds (Supplementary Fig. S12), and in the KEGG pathways of galactose metabolism and sucrose metabolism (Supplementary Fig. S13).

The enrichment of single-copy and multiple-copy genes was analyzed in 11 plant species to investigate species-specific gene families, common gene families, homologous genes, and gene family clusters. (Fig. 2B). *P. praeruptorum* carries 44,468 genes, clustered into 19,402 gene families containing 489 single-copy gene families (Fig. 2B and Supplementary Table S15). Among the gene families, 4,016 common gene families were shared with other plant species, and 741 gene families were specific to *P. praeruptorum* (Fig. 2C). We further performed collinearity analysis between *P. praeruptorum, D. carota, C. sativum*, and *A. sinensis*. The results showed a few major chromosomal rearrangements occurred between those species (Fig. 2D and Supplementary Fig. S16). The distribution of synonymous substitutions per synonymous site (Ks) for Apiaceae plants was compared. Two major peaks were observed, consistent with the previous hypothesis that *P. praeruptorum* experienced 2 WGD events (Fig. 2E).

As the first species to be separated in our phylogeny in the Apiaceae, *D. carota* harbors 9 chromosomes in a haploid. In contrast, many other members (e.g., *A. graveolens, A. sinensis, C. sativum, P. praeruptorum, Thapsia garganica, Thapsia smittii*) all have 11 chromosomes in a haploid (Supplementary Fig. S17) [13, 65, 66]. The complete dotplot-based deconvolution into 11 reconstructed conserved ancestral regions (CARs) of the observed synteny and paralogy among *P. praeruptorum* and its Apiaceae siblings suggested the 11 proposed protochromosomes as the origin of Apiaceae. Our analysis is also consistent with the previous report that modern celery chromosomes are well represented by the Apiaceae protochromosomes (Supplementary Fig. S17) [13]. In addition, comparing *D. carota* with the other Apiaceae members with 11 chromosomes indicated that chromosome 10 and chromosome 6 experienced fission and fusion that reduced chromosome numbers in *D. carota* (Supplementary Fig. S17).

### Biosynthesis of coumarins

**Figure 3:**
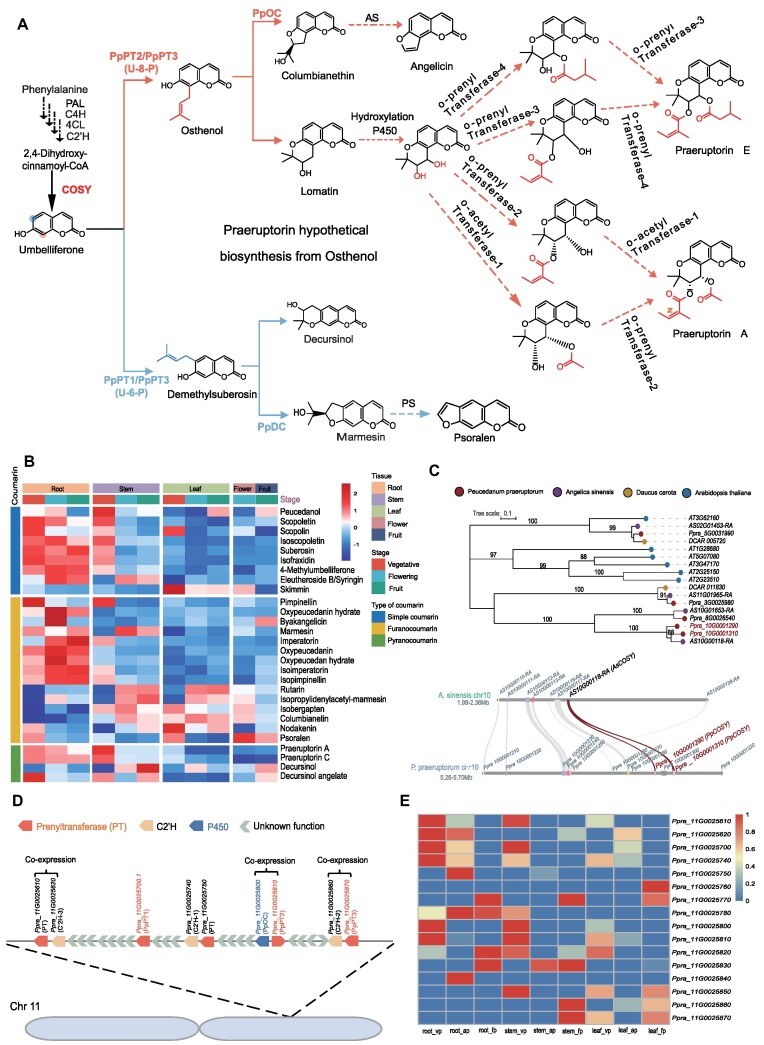
The coumarin biosynthesis pathway in *P. praeruptorum*. (A) The coumarin biosynthesis candidate gene identification in *P. praeruptorum*. (B) The distribution of coumarins in 5 different tissues and 3 different developmental periods. (C) The phylogenetic analysis of COSY and collinearity analysis between *A. sinensis* and *P. praeruptorum*. (D) Chromosomal mapping and gene cluster identification of coumarin biosynthetic genes: prenyltransferases (*PT*s), *PpOC*, and *p-*coumaroyl CoA 2′-hydroxylases (*C*′*2H*). (E) Expression profiling of candidate genes pairs in the coumarin synthesis pathway.

The biosynthesis of coumarins is initiated at the phenylpropanoid pathway, whereas L-phenylalanine is catalyzed by PAL to form cinnamic acid (Fig. 3A). The cinnamic acid is further converted to *p*-coumaric acid by C4H and transformed into *p*-coumaroyl CoA by a member of the 4CL family. The CoA-esters are subsequently hydroxylated at the position ortho to the aromatic ring aliphatic side chain through either C2′H. The coumarin core structure (e.g., umbelliferone in the roots) is catalyzed by coumarin synthase (COSY) [58]. For *P. praeruptorum*, the biosynthetic genes (*PAL, C4H, 4CL, C2*′*H*) in the initial steps of the phenylpropanoid pathway showed diverse spatial and temporal expression patterns since these compounds are common precursors for downstream anthocyanin, lignin, and flavonoid pathways [67] (Supplementary Fig. S19). The genes (*C2*′*H, COSY, U-8-P*, and *U-6-P*) involved in the formation of umbelliferone and its derivatives are mainly expressed in the roots and stems at the vegetative stage (Supplementary Tables S18 and S19). However, during the reproductive stage (e.g., anthesis and fruit), these transcripts showed reduced expression, which is also supported by the metabolomic data (Fig. 3B). The metabolomic analysis showed that the major forms of coumarins could be detected in the roots during the growth stages (Fig. 3B). This was consistent with previous reports where coumarins were secreted into the rhizosphere due to their allelopathic properties or were involved in iron acquisition [63]. A few coumarins such as skimmin, rutarin, isopropylidenylacetyl-marmesin, isobergapten, and decursinol were mainly observed in the leaves (Fig. 3B), but in general, the coumarins could be found in the roots and stems (Supplementary Table S20).

### Coumarin synthase (COSY)

Previously, biochemical and molecular experiments done in the *Ruta graveolens* and Arabidopsis suggested that the *trans–cis* isomerization and lactonization forming the coumarin core structure was a spontaneous reaction catalyzed by lights [68–71]. However, a BAHD family member named coumarin synthase (COSY) was cloned in Arabidopsis and was demonstrated to catalyze the reaction without light (Fig. 3). Since COSY is a key gene for the ring closing in coumarin skeleton, we specifically examine their expression and evolution in Apiaceae. COSY is mainly expressed in the roots, an organ away from lights, and is a conserved gene across many plant species [72]. We used 4 *A. sinesis* COSY (*AS10G01653, AS02G01453, AS10G00118*, and *AS11G01965*) as the query to identify COSY genes in 2 other Apiaceae plants (*P. praeruptorum* and *D. carota*) (Supplementary Table S21). Phylogenetic analysis of plant COSY enzymes showed that there are 5 major clades. Interestingly, there is a multicollinearity comparison between *P. praeruptorum* and *A. sinensis*, and there is 1 more copy of *COSY* in *P. praeruptorum* on chromosome 10, which occurred by tandem duplication after *P. praeruptorum* diverged from *A. sinensis* (Fig. 3C and Fig. 2A). The expression patterns of this pair were slightly different at developmental stages. The expression of *Ppra_10G0001290* was downregulated at the anthesis and fruiting periods (Fig. 3A). This suggests that these 2 genes may play different roles in coping with developmental needs. However, the detailed functions still need to be clarified.

The biosynthesis of complex coumarins following the umbelliferone has recently been described in *P. praeruptorum*. Prenylation of the umbelliferone carbon skeleton 6 or skeleton 8, followed by subsequent cyclization, are regarded as crucial steps to form furanocoumarins or pyranocoumarins. These steps play roles in determining the linear or the angular structures of either furanocoumarins or the pyranocoumarins [8]. Seven prenyltransferases (PTs) in total were identified based on the transcriptome and metabolome data analysis, and 6 of 7 prenyltransferases with catalytic activities of prenylating the umbelliferone were characterized (Supplementary Fig. S20). PpPT1 (ON934685) has a umbelliferone 6-prenyltransferase (U6P) activity, and PpPT2 (ON934686) has a umbelliferone 8-prenyltransferase (U8P) activity with a minor U6P activity. PpPT3 (ON934687) has both U6P and U8P activities, whereas the rest of the 3 homologs have weak U6P activities [9]. Additionally, 2 CYP P450 monooxygenases, PpDC (ON934691) and PpOC (ON934692), have been identified [9].

Genes that play role in the production of specific metabolites are frequently grouped in clusters to enable synchronized expression, a phenomenon common in plants and described for many different specialized metabolites [73–77]. Thus, we used the sequence information provided above and blasted the genome we had in hand. Interestingly, all 3 major PTs (PT1–3) are located on chromosome 11 (Fig 3D). Additionally, the *PpOC* (*ON934692*) is located on chromosome 11 and forms a functional gene pair with *PpPT2* (*ON934686*). We looked into the details of gene annotations in this region and identified 3 other gene pairs comprising *PT* and *C*′*2H*. Frequently, gene pairs usually undergo tight regulation together at the nucleosome level and are coexpressed together [78]. We checked the expression pattern between these gene pairs. Notably, the *PpOC* (*ON934692*) and *PpPT2* (*ON934686*) display similar expression patterns in a spatial and temporal manner (Fig. 3E). The expressions of *PT1/PT2/PpOC/PpDC* were double-checked with qPCR and showed high correlations with the transcriptome (Supplementary Fig. S19). The other 3 pairs also showed similar expression except for the gene pair of *Ppra_11G0025740* and *Ppra_11G0025750*. These 2 genes displayed similar root-specific expression but with opposite temporal expression patterns (Fig. 3E). Here, we demonstrated how a well-assembled genome could provide a roadmap to pathway elucidation. However, how these gene pairs function in the coumarin biosynthetic pathway requires further exploration.

### Cytochrome P450 genes in coumarin biosynthesis

**Figure 4:**
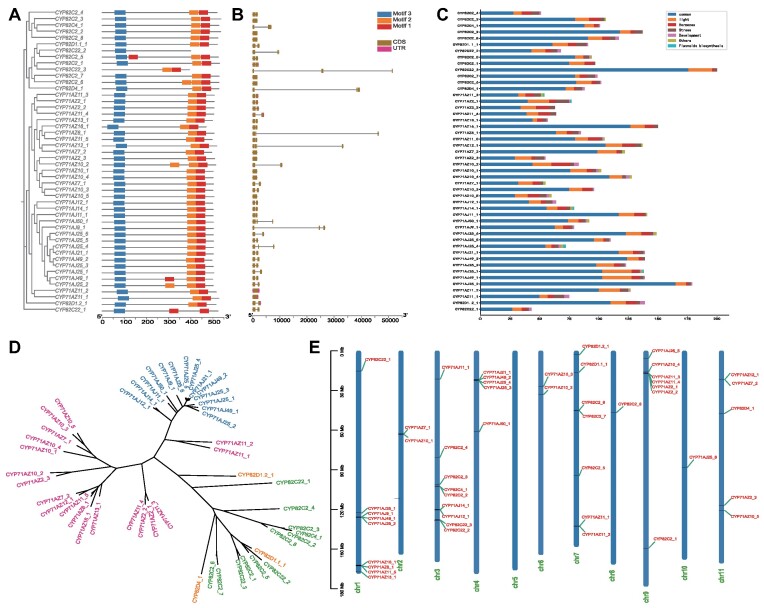
Cytochrome P450 genes related to coumarin biosynthesis in *P. praeruptorum*. (A) Motif analysis in *cis*-elements of the coumarin biosynthesis-related cytochrome P450 genes. (B) The gene structures of the coumarin biosynthesis-related cytochrome P450 genes. (C) The analysis of key *cis*-elements identified in the promoter regions of coumarin-related cytochrome P450 genes. (D) Phylogenetic tree of cytochrome P450 genes involved in coumarin biosynthesis. (E) The gene location of coumarin-related cytochrome P450 genes on the chromosomes.

Plant cytochrome P450s catalyze several regio- and stereo-specific hydroxylations that play important roles in general and specialized metabolite biosynthesis [79–81]. Based on the radioactive labeling of *Ammi majus* cell cultures, it is suggested that the cytochrome P450s are involved in coumarin biosynthesis [82]. Thus, to identify the functional genes, we initially screened all the genes within the CYP71 family in the *P. praeruptorum* genome. This approach was based on the hypothesis that the isopentene group cyclization mechanism shares similarities with the CYP71 menthofuran synthase derived from *Mentha piperita*. Moreover, several members of the CAYP71AJ subfamily have been cloned and characterized [83, ]. The *AmCYP71AJ1* cloned from *A. majus* (Apiaceae) is responsible for catalyzing the linear furanocoumarin formation [71]. In contrast, the *PsCYP71AJ4* from *Pastinaca sativa* is an angelicin synthase, which is an angular furanocoumarin and has been modeled as well [70, 84]. Additionally, we analyzed a total of 48 cytochrome P450 genes by homology comparison with CYP71AJ gene family members of *A. sinesis* (Fig. 4). The phylogenetic tree demonstrated that these genes were distributed into 3 major lineages (e.g., *PpCYP71AJ, PpCYP71AZ*, and *PpCYP82C*). Interestingly, the members of *PpCYP71AZ* and *PpCYP82C* are expanded significantly in *P. praeruptorum* when compared with other Apiaceae members (e.g., *A. sinensis, C. sativum*, and *P. notoginseng*) (Fig. 4D and Supplementary Table S22). These expanded genes may be responsible for catalyzing the successive regio- and stereo-specific hydroxylation in the complex coumarin biosynthesis. This result is consistent with the diverse coumarins detected in the *P. praeruptorum* (Fig. 3 and Supplementary Table S20). Based on the gene annotations, these CYP genes all shared similar gene structures, which contain 2 main CDS and 3 major motifs in their *cis*-elements (Fig. 4A and B). In addition to the common *cis*-elements, light, hormone, and stress-responsive elements were the 3 major types. This suggests that the whole gene duplication events are the major drive to expand this gene family. Several tandem and proximal duplications were observed except on chromosomes 5, 8, and 10. Interestingly, no *PpCYP71* or *PpCYP82* members can be identified on chromosome 5 (Fig. 4E).

### The systematic regulation of coumarin biosynthesis

**Figure 5:**
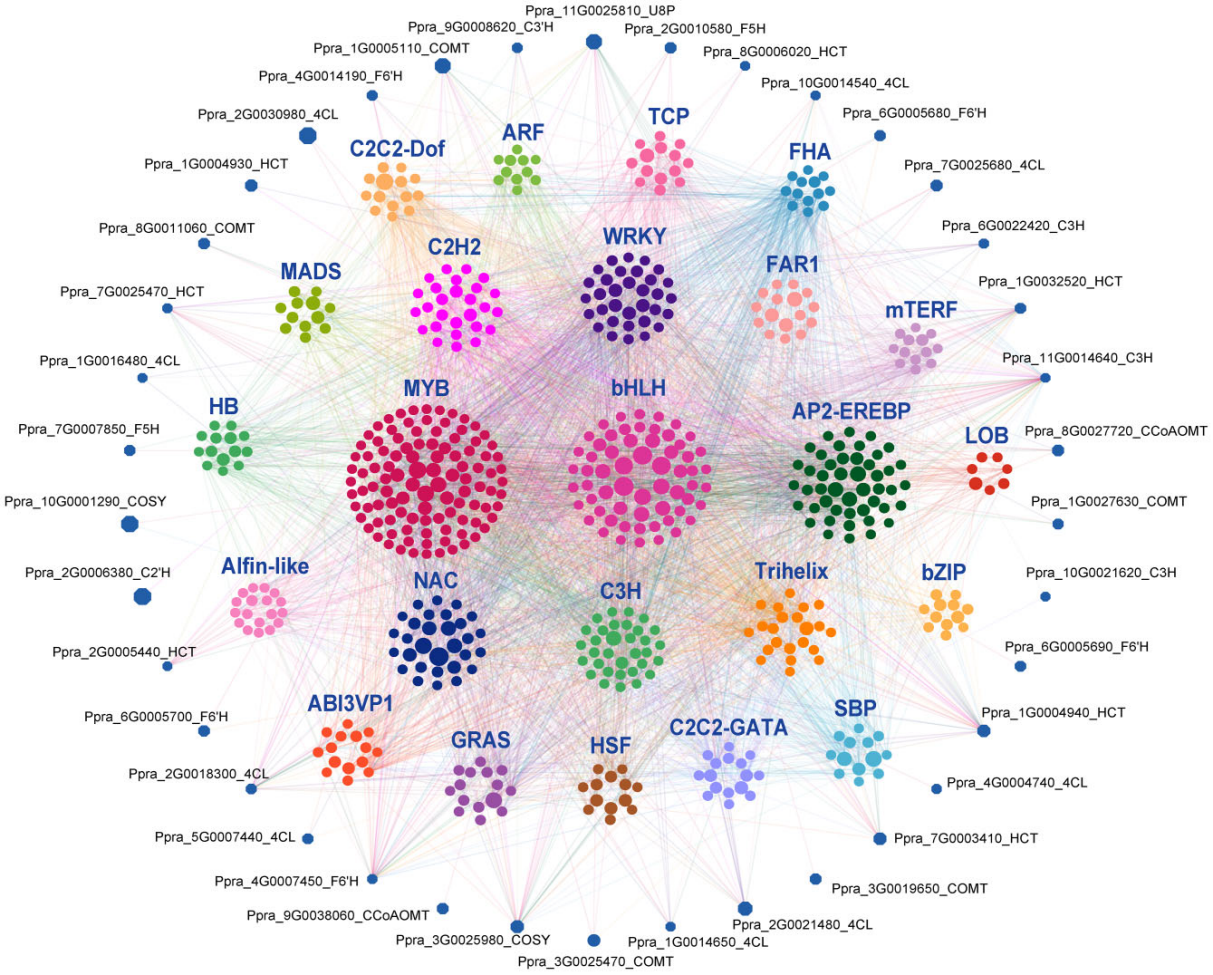
A coexpression network connecting structural genes in coumarin biosynthesis with transcription factors (TFs) represents the regulation of coumarin biosynthetic genes. The nodes represent structural genes in coumarin biosynthesis and transcription factors. The node size shows the expression changes of each gene (log_2_ fold change (root_fp/root_vp)). The numbers of the nodes demonstrate the number of TFs associated with the core biosynthetic genes.

Since the *cis*-elements of *PpCYP71AJ* members showed diverse regulatory elements, we developed a systematic view of the regulatory network of coumarin biosynthesis and the potential transcription factors (TFs) associated with these genes. A coexpression network connecting key node genes in coumarin biosynthesis with TFs was analyzed. The expression patterns of *C3H* (*Ppra_11G0014640*), *4CL* (*Ppra_2G0018300, Ppra_2G0018300*), *F6*′*H* (*Ppra_4G0007450*), and *COSY* (*Ppra_3G0025980*) were found to be highly related to the expression patterns of numerous TFs (Fig. 5). The MYB, bHLH, AP2-EREBP, and WRKY were the 4 major TFs regulating coumarin biosynthesis. The R2R3-MYB and bHLH were well known that they, together with WD40, form ternary complexes that positively or negatively regulate flavonoid biosynthesis genes [85, 86]. The simple coumarin scopolin was known to be accumulated under abiotic stress conditions [87]. Based on our results, the AP2-EREBP and WRKY were biotic and/or abiotic stress response–related TFs, which further reflected the biosynthesis of these diverse coumarins to cope with various stress conditions.

### Terpene biosynthesis

Terpenoids, a class of natural products that are rich in herbal plants, have been widely studied for their therapeutic efficacy, especially in Apiaceae [88–90]. The distribution of terpenoids was examined in the root/stem and leaf with 3 different developmental stages. These compounds are widely distributed in the various tissues in *P. praeruptorum* (Supplementary Fig. S18C). To identify the key TPS genes involved in the production of major terpenoids, we conducted a comprehensive analysis of the *P. praeruptorum* genome. The full-length *PpTPS* genes were obtained through genome scanning (Supplementary Fig. S18). A total of 48 *PpTPS* genes were identified and categorized into 6 subfamilies (*PpTPS-a, b, c, e, f*, and *g*) based on phylogenetic analysis, following the previously established nomenclature (Supplementary Table S16) [91]. It is worth mentioning that the *TPS-a* and *TPS-b* subfamilies have the largest number of members (Supplementary Fig. S18B). Specifically, the *PpTPS-b* subfamily, which encodes angiosperm-specific monoterpene synthases, was substantially expanded with 24 members (Supplementary Fig. S18B, Supplementary Table S17). Most of the TPSs are expressed during the vegetative stages in the root, the stem, and the leaf (Supplementary Fig. S18B). By analyzing their chromosomal localization, the distribution of *PpTPS* genes is across all 11 chromosomes. Chr04 hosts the greatest abundance of *TPS* genes, amounting to a total of 12 genes. Among them, 10 genes have formed pairs through tandem duplication events (Supplementary Fig. S18D).

## Discussion

*P. praeruptorum* is a valuable Chinese medicinal plant commonly used to treat coughing and as an antimucus agent. Among the bioactive compounds, coumarins show high bioactivity to reduce multidrug resistance in cancer cells with low toxicity [92]. Thus, *P. praeruptorum* is considered a great resource for isolating these compounds. High-quality reference genomes could help uncover important traits and elucidate key catalytic enzymes for synthetic biology. A chromosome-level genome assembly of *Artemisia annua* revealed the artemisinin content is correlated to the copy number of amorpha-4,11-diene synthase genes, as one representative example of how genomic information can help to improve plant-specific metabolism [93]. Here, we present the first T2T genome of *P. praeruptorum*. Comparing the distribution of synonymous substitutions per synonymous site (Ks) in *P. praeruptorum* and the other Apiaceae species shows that the Apiaceae members experienced 2 WGD events. This is consistent with the previously published data and may be a distinctive genomic signature of the Apiaceae family [11, 16, 94]. A chromosomal collinear analysis compared *P. praeruptorum, D. carota*, and *A. sinensis* to reconstruct plant chromosome evolution. Several chromosomal rearrangements have occurred to reshape the genome landscape of *P. praeruptorum* and are disclosed here (Fig. 2D and Supplementary Fig. S16).

The molecular basis of coumarin biosynthesis and their distribution have been described previously [4]. Most coumarins we evaluated here were also detected in the roots, which is consistent with the expression patterns of its biosynthetic genes. However, we also detected some of the coumarins solely accumulated in the aerial parts, which had not been disclosed in the past (Fig. 3B).

P450s are a well-established enzyme superfamily present across various organisms. Their primary function involves catalyzing monooxygenation/oxidation reactions, making them valuable tools for constructing intricate molecules [79, 95]. These enzymes play essential roles in complex metabolic networks, serving as major contributors to phytochemical diversification and aiding in adaptation to fluctuating environmental conditions [79, 96]. In the late 1980s, Hamerski and Mattern identified that a P450 in *Ammi majus* served as the marmesin synthase [97]. In *P. praeruptorum*, 2 P450s (e.g., PpOC and PpDC) belonging to CYP71 or CYP71-clan families have been identified [98]. In addition, the physical clustering of homologous P450s, often observed in recently tandem-duplicated P450s, indicates active evolutionary dynamics favoring the acquisition of new activities. It also suggests that some of the clustered genes may function in the same pathway [79, 96]. This clustering phenomenon has been previously identified in genes associated with furanocoumarin biosynthesis, as seen with CYP71AJ3 and CYP71AJ4 in *Pastinaca sativa* [99]. With this T2T genome, we recognized *PpPT2* and *PpOC* are clustered as functional gene pairs and 2 other annotated *PT* and *C2*′*H* gene pairs on chromosome 11 (Fig. 3D). With the elucidation of PpOC that catalyzes the formation of lomatin, the enzyme that is involved in the hydroxylation of C-4′ skeleton to form a khellactone is still elusive. Since several hydroxylases form functional gene pairs with the PTs and these genes are coexpressed, this gives a hotspot for mining the candidate enzymes for C-4′ skeleton hydroxylation in this region. However, this hypothesis needs to be further confirmed biochemically in the future.

## Supplementary Material

giae025_supplement

giae025_GIGA_D_23_00282_Original_Submission

giae025_GIGA_D_23_00282_Revision_1

giae025_GIGA_D_23_00282_Revision_2

giae025_Response_to_Reviewer_Comments_Original_Submission

giae025_Response_to_Reviewer_Comments_Revision_1

giae025_Reviewer_1_Report_Original_Submission Wei Sun -- 11/27/2023

giae025_Reviewer_1_Report_Revision_1 Wei Sun -- 3/7/2024

giae025_Reviewer_2_Report_Original_Submission Li Wang -- 11/28/2023

giae025_Reviewer_2_Report_Revision_1 Li Wang -- 3/15/2024

## Data Availability

The genome sequencing data, including PacBio HiFi, ONT Ultra-long, DNBseq short reads, Hi-C data, and transcriptome data, have been deposited into the NCBI database and are available via the BioProject accession number PRJNA1011536. The genome assemblies and gene annotations have been deposited at Figshare [100]. All additional supporting data are available in the *GigaScience* repository, GigaDB [101].

## Additional Files

**Supplementary Fig. S1**. The *k*-mer depth distribution for *Peucedanum praeruptorum* Dunn genome size evaluation.

**Supplementary Fig. S2**. The pipeline for the assembly of the T2T genome of *Peucedanum praeruptorum* Dunn.

**Supplementary Fig. S3**. Statistical graph of correlation analysis between GC content and depth (short and long reads).

**Supplementary Fig. S4**. The gene structure prediction results and gene set statistics (comparison with gene elements of closely related species).

**Supplementary Fig. S5**. The cumulative distribution statistics graph of gene set element length (comparison with gene elements of closely related species).

**Supplementary Fig. S6**. Venn diagram of homologous gene families of *Peucedanum praeruptorum* Dunn in the genome.

**Supplementary Fig. S7**. GO enrichment annotation of *Peucedanum praeruptorum* Dunn.

**Supplementary Fig. S8**. KEGG enrichment annotation of *Peucedanum praeruptorum* Dunn.

**Supplementary Fig. S9**. Venn diagram of functional annotation of *Peucedanum praeruptorum* Dunn in different databases.

**Supplementary Fig. S10**. The syntenic comparison with the newly published genome of *Peucedanum praeruptorum* Dunn.

**Supplementary Fig. S11**. The phylogenetic tree of the 11 species genomes with 489 single-copy genes.

**Supplementary Fig. S12**. GO enrichment results of *Peucedanum praeruptorum* Dunn contraction gene family.

**Supplementary Fig. S13**. KEGG enrichment results of *Peucedanum praeruptorum* Dunn contraction gene family.

**Supplementary Fig. S14**. GO enrichment results of *Peucedanum praeruptorum* Dunn expansion gene family.

**Supplementary Fig. S15**. KEGG enrichment results of *Peucedanum praeruptorum* Dunn expansion gene family.

**Supplementary Fig. S16**. The gene synthetic compaction among *Angelica sinensis, Peucedanum praeruptorum* Dunn, and *Daucus carota*.

**Supplementary Fig. S17**. Inference of polyploidization and speciation history in Apiaceae.

**Supplementary Fig. S18**. Gene family identification and analysis of the terpene biosynthetic pathway.

**Supplementary Fig. S19**. Gene family identification and analysis of the coumarin biosynthetic pathway.

**Supplementary Fig. S20**. The phylogenetic tree of different species genomes with PT genes.

**Supplementary Table S1**. Statistical results of DNBSEQ platform sequencing data for genome survey.

**Supplementary Table S2**. Summary statistics for PacBio HiFi data, ONT ultra-long, and Hi-C data.

**Supplementary Table S3**. Statistics of genome assembly result of *Peucedanum praeruptorum* Dunn species.

**Supplementary Table S4**. Statistics of the T2T assembled chromosomes of *Peucedanum praeruptorum* Dunn.

**Supplementary Table S5**. Telomere and centromere sequence identification.

**Supplementary Table S6**. Completeness estimation of *Peucedanum praeruptorum* Dunn genome with BUSCO analysis.

**Supplementary Table S7**. The statistical results of the repeat sequence.

**Supplementary Table S8**. Repeat sequence classification result statistics.

**Supplementary Table S9**. Basic statistical results of gene annotation prediction.

**Supplementary Table S10**. Statistics of genes annotated in the *Peucedanum praeruptorum* Dunn genome.

**Supplementary Table S11**. The statistical results of gene function annotation.

**Supplementary Table S12**. Statistics of noncoding RNA annotation results.

**Supplementary Table S13**. The comparison of assembly and annotation with the newly published genome of *Peucedanum praeruptorum* Dunn.

**Supplementary Table S14**. The comparison of chromosomes with the newly published genome of *Peucedanum praeruptorum* Dunn.

**Supplementary Table S15**. Summary of gene family clustering.

**Supplementary Table S16**. Gene family identification and the phylogenetic analysis of the terpene biosynthetic pathway.

**Supplementary Table S17**. Expression profiles of TPS gene family in different tissues.

**Supplementary Table S18**. The genes associated with the coumarin metabolic pathway and the expression levels of those genes in various tissues and different growth stages.

**Supplementary Table S19**. The statistical results of the PT gene in the 4 species were utilized for analysis.

**Supplementary Table S20**. The coumarin compounds data obtained through HPLC-MS analysis in different tissues.

**Supplementary Table S21**. The statistical results of the COSY genes in 3 species were utilized for analysis.

**Supplementary Table S22**. Phylogenomic analysis of cytochrome P450 multigene family.

**Supplementary Table S23**. The primers used in this study.

## Abbreviations

BLAST: Basic Local Alignment Search Tool; BUSCO: Benchmarking Universal Single-Copy Orthologs; CDS: coding sequence; Chr: chromosome; DUP: duplication; Gb: gigabase; GO: Gene Ontology; HPLC: high-performance liquid chromatography; KEGG: Kyoto Encyclopedia of Genes and Genomes; LINE: long interspersed nucleotide element; LTR: long terminal repeat; Mb: megabase; TD: tandem duplication; TE: transposable element; WGD: whole-genome duplication.

## References

[1] Song Y, Jing W, Yan R et al. Research progress of the studies on the roots of *Peucedanum praeruptorum* dunn (Peucedani radix). Pak J Pharm Sci. 2015;28:71–81. 25553708

[2] Seigler DS . Coumarins. In: Plant Secondary Metabolism. New York, NY: Springer US, 1998.. 10.1007/978-1-4615-4913-0_9.

[3] Author A, Berenbaum MR. Chemical Mediation of Coevolution: Phylogenetic Evidence for. Source: Annals of the Missouri Botanical Garden. Missouri, United States:Missouri Botanical Garden Press, 2001:45–59. 10.2307/2666131.

[4] Robe K, Izquierdo E, Vignols F, et al. The coumarins: secondary metabolites playing a primary role in plant nutrition and health. Trends Plant Sci. 2021;26:248–59. 10.1016/j.tplants.2020.10.008. 33246890

[5] Bourgaud F, Hehn A, Larbat R et al. Biosynthesis of coumarins in plants: a major pathway still to be unravelled for cytochrome P450 enzymes. Phytochem Rev. 2006;5:293–308. 10.1007/s11101-006-9040-2.

[6] Rodrigues JL, Rodrigues LR. Biosynthesis and heterologous production of furanocoumarins: perspectives and current challenges. Nat Prod Rep. 2021;38:869–79. 10.1039/D0NP00074D. 33174568

[7] Del Río JA, Díaz L, García-Bernal D, et al. Furanocoumarins: biomolecules of therapeutic interest. In: Atta-ur-Rahman, Studies in Natural Products Chemistry, vol. 43, Amsterdam, The Netherlands: Elsevier, 10.1016/B978-0-444-63430-6.00005-9.

[8] Karamat F, Olry A, Munakata R, et al. A coumarin-specific prenyltransferase catalyzes the crucial biosynthetic reaction for furanocoumarin formation in parsley. Plant J. 2014;77:627–38. 10.1111/tpj.12409. 24354545

[9] Zhao Y, He Y, Han L, et al. Two types of coumarins-specific enzymes complete the last missing steps in pyran- and furanocoumarins biosynthesis. Acta Pharmaceutica Sinica B. 2024;14:869–80. 10.1016/j.apsb.2023.10.016. 38322336 PMC10840424

[10] Chu S, Chen L, Xie H, et al. Comparative analysis and chemical profiling of different forms of Peucedani radix. J Pharm Biomed Anal. 2020;189:113410. 10.1016/j.jpba.2020.113410. 32574998

[11] Wang Y-H, Liu P-Z, Liu H et al. Telomere-to-telomere carrot (*Daucus carota*) genome assembly reveals carotenoid characteristics. Hortic Res. 2023;10:1–12. 10.1093/hr/uhad103. PMC10541555 37786729

[12] Song X, Wang J, Li N et al. Deciphering the high-quality genome sequence of coriander that causes controversial feelings. Plant Biotechnol J. 2020;18:1444–56. 10.1111/pbi.13310. 31799788 PMC7206992

[13] Song X, Sun P, Yuan J et al. The celery genome sequence reveals sequential paleo-polyploidizations, karyotype evolution and resistance gene reduction in apiales. Plant Biotechnol J. 2021;19:731–44. 10.1111/pbi.13499. 33095976 PMC8051603

[14] Li MY, Feng K, Hou XL et al. The genome sequence of celery (Apium graveolens L.), an important leaf vegetable crop rich in apigenin in the Apiaceae family. Hortic Res. 2020;7:1–10. 10.1038/s41438-019-0235-2. 31934340 PMC6944684

[15] Li S, Chiu TY, Jin X, et al. Integrating genomic and multiomic data for Angelica sinensis provides insights into the evolution and biosynthesis of pharmaceutically bioactive compounds. Commun Biol. 2023;6:1–20. 10.1038/s42003-023-05569-5. 38001348 PMC10674023

[16] Han X, Li C, Sun S, et al. The chromosome-level genome of female ginseng (*Angelica sinensis*) provides insights into molecular mechanisms and evolution of coumarin biosynthesis. Plant J. 2022;112:1224–37. 10.1111/tpj.16007. 36259135

[17] Zhang Q, Li M, Chen X, et al. Chromosome-level genome assembly of bupleurum chinense DC provides insights into the saikosaponin biosynthesis. Front Genet. 2022;13:1–11. 10.3389/fgene.2022.878431. PMC9008701 35432473

[18] Song C, Zhang Y, Manzoor MA et al. A chromosome-scale genome of *Peucedanum praeruptorum* provide insights into Apioideae evolution and medicinal ingredient biosynthesis. Int J Biol Macromol. 2024;255:128218. 10.1016/j.ijbiomac.2023.128218. 37992933

[19] Chin C-S, Alexander DH, Marks P, et al. Nonhybrid, finished microbial genome assemblies from long-read SMRT sequencing data. Nat Methods. 2013;10:563–69. 10.1038/nmeth.2474. 23644548

[20] Durand NC, Robinson JT, Shamim MS et al. Juicebox provides a visualization system for hi-C contact maps with unlimited zoom. Cell Syst. 2016;3:99–101. 10.1016/j.cels.2015.07.012. 27467250 PMC5596920

[21] Marçais G, Kingsford C. A fast, lock-free approach for efficient parallel counting of occurrences of k-mers. Bioinformatics. 2011;27:764–70. 10.1093/bioinformatics/btr011. 21217122 PMC3051319

[22] Vurture GW, Sedlazeck FJ, Nattestad M, et al. GenomeScope: fast reference-free genome profiling from short reads. Bioinformatics. 2017;33:2202–4. 10.1093/bioinformatics/btx153. 28369201 PMC5870704

[23] Cheng H, Concepcion GT, Feng X et al. Haplotype-resolved de novo assembly using phased assembly graphs with hifiasm. Nat Methods. 2021;18:170–75. 10.1038/s41592-020-01056-5. 33526886 PMC7961889

[24] Roach MJ, Schmidt SA, Borneman AR. Purge Haplotigs: allelic contig reassignment for third-gen diploid genome assemblies. BMC Bioinf. 2018;19:1–10. 10.1186/s12859-018-2485-7. PMC6267036 30497373

[25] Dudchenko O, Batra SS, Omer AD, et al. De novo assembly of the Aedes aegypti genome using Hi-C yields chromosome-length scaffolds. Science. 2017;356:92–95. 10.1126/science.aal3327. 28336562 PMC5635820

[26] Robinson JT, Turner D, Durand NC et al. Juicebox.Js provides a cloud-based visualization system for Hi-C data. Cell Syst. 2018;6:256–58.. 10.1016/j.cels.2018.01.001. 29428417 PMC6047755

[27] Li H . Minimap2: pairwise alignment for nucleotide sequences. Bioinformatics. 2018;34:3094–100. 10.1093/bioinformatics/bty191. 29750242 PMC6137996

[28] Xu M, Guo L, Gu S, et al. TGS-GapCloser: a fast and accurate gap closer for large genomes with low coverage of error-prone long reads. Gigascience. 2020;9:1–11. 10.1093/gigascience/giaa094. PMC7476103 32893860

[29] Wang Y, Zhao Y, Bollas A, et al. Nanopore sequencing technology, bioinformatics and applications. Nat Biotechnol. 2021;39(11):1348–65. 10.1038/s41587-021-01108-x. 34750572 PMC8988251

[30] Camacho C, Coulouris G, Avagyan V, et al. BLAST+: architecture and applications. BMC Bioinf. 2009;10:1–9. 10.1186/1471-2105-10-421. PMC2803857 20003500

[31] Walker BJ, Abeel T, Shea T et al. Pilon: an integrated tool for comprehensive microbial variant detection and genome assembly improvement. PLoS One. 2014;9:e112963 10.1371/journal.pone.0112963. 25409509 PMC4237348

[32] Manni M, Berkeley MR, Seppey M, et al. BUSCO update: novel and streamlined workflows along with broader and deeper phylogenetic coverage for scoring of eukaryotic, prokaryotic, and viral genomes. Mol Biol Evol. 2021;38:4647–54. 10.1093/molbev/msab199. 34320186 PMC8476166

[33] Benson G . Tandem repeats finder: a program to analyze DNA sequences. Nucleic Acids Res. 1999;27:573–80. 10.1093/nar/27.2.573. 9862982 PMC148217

[34] Saha S, Bridges S, Magbanua ZV, et al. Empirical comparison of ab initio repeat finding programs. Nucleic Acids Res. 2008;36:2284–94. 10.1093/nar/gkn064. 18287116 PMC2367713

[35] Bao W, Kojima KK, Kohany O. Repbase Update, a database of repetitive elements in eukaryotic genomes. Mobile DNA. 2015;6:1–6. 10.1186/s13100-015-0041-9. PMC4455052 26045719

[36] Flynn JM, Hubley R, Goubert C, et al. RepeatModeler2 for automated genomic discovery of transposable element families. Proc Natl Acad Sci USA. 2020;117:9451–57. 10.1073/pnas.1921046117. 32300014 PMC7196820

[37] Xu Z, Wang H. LTR_FINDER: an efficient tool for the prediction of full-length LTR retrotransposons. Nucleic Acids Res. 2007;35:W265–68. 10.1093/nar/gkm286. 17485477 PMC1933203

[38] Slater G, Birney E. Automated generation of heuristics for biological sequence comparison. BMC Bioinf. 2005;6:1–11. 10.1186/1471-2105-6-31. PMC553969 15713233

[39] Shumate A, Salzberg SL. Liftoff: accurate mapping of gene annotations. Bioinformatics. 2021;37:1639–43. 10.1093/bioinformatics/btaa1016. 33320174 PMC8289374

[40] Stanke M, Schöffmann O, Morgenstern B, et al. Gene prediction in eukaryotes with a generalized hidden Markov model that uses hints from external sources. BMC Bioinf. 2006;7:1–11. 10.1186/1471-2105-7-62. PMC1409804 16469098

[41] Korf I . Gene finding in novel genomes. BMC Bioinf. 2004;5:1–9. 10.1186/1471-2105-5-59. PMC421630 15144565

[42] Kim D, Langmead B, Salzberg SL. HISAT: a fast spliced aligner with low memory requirements. Nat Methods. 2015;12:357–60. 10.1038/nmeth.3317. 25751142 PMC4655817

[43] Kovaka S, Zimin AV, Pertea GM, et al. Transcriptome assembly from long-read RNA-seq alignments with StringTie2. Genome Biol. 2019;20:278. 10.1186/s13059-019-1910-1. 31842956 PMC6912988

[44] Holt C, Yandell M. MAKER2: an annotation pipeline and genome-database management tool for second-generation genome projects. BMC Bioinf. 2011;12:419. 10.1186/1471-2105-12-491. PMC3280279 22192575

[45] Chan PP, Lin BY, Mak AJ et al. TRNAscan-SE 2.0: improved detection and functional classification of transfer RNA genes. Nucleic Acids Res. 2021;49:9077–96. 10.1093/nar/gkab688. 34417604 PMC8450103

[46] Kalvari I, Nawrocki EP, Ontiveros-Palacios N et al. Rfam 14: expanded coverage of metagenomic, viral and microRNA families. Nucleic Acids Res. 2021;49:D192–D200. 10.1093/nar/gkaa1047. 33211869 PMC7779021

[47] Li L, Stoeckert CJ, Roos DS. OrthoMCL: identification of ortholog groups for eukaryotic genomes. Genome Res. 2003;13:2178–89. 10.1101/gr.1224503. 12952885 PMC403725

[48] Katoh K, Standley DM. MAFFT multiple sequence alignment software version 7: improvements in performance and usability. Mol Biol Evol. 2013;30:772–80. 10.1093/molbev/mst010. 23329690 PMC3603318

[49] Castresana J . Selection of conserved blocks from multiple alignments for their use in phylogenetic analysis. Mol Biol Evol. 2000;17:540–52. 10.1093/oxfordjournals.molbev.a026334. 10742046

[50] Stamatakis A . RAxML version 8: a tool for phylogenetic analysis and post-analysis of large phylogenies. Bioinformatics. 2014;30:1312–13. 10.1093/bioinformatics/btu033. 24451623 PMC3998144

[51] Yang Z . PAML 4: phylogenetic analysis by Maximum likelihood. Mol Biol Evol. 2007;24:1586–91. 10.1093/molbev/msm088. 17483113

[52] Sun P, Jiao B, Yang Y et al. WGDI: a user-friendly toolkit for evolutionary analyses of whole-genome duplications and ancestral karyotypes. Mol Plant. 2022;15:1841–51. 10.1016/j.molp.2022.10.018. 36307977

[53] Chen Y, Chen Y, Shi C et al. SOAPnuke: a MapReduce acceleration-supported software for integrated quality control and preprocessing of high-throughput sequencing data. Gigascience. 2018;7:1–6. 10.1093/gigascience/gix120. PMC5788068 29220494

[54] Langmead B, Salzberg SL. Fast gapped-read alignment with Bowtie 2. Nat Methods. 2012;9:357–59. 10.1038/nmeth.1923. 22388286 PMC3322381

[55] Li B, Dewey CN. RSEM: accurate transcript quantification from RNA-seq data with or without a reference genome. BMC Bioinf. 2011;12:323. 10.1186/1471-2105-12-323. PMC3163565 21816040

[56] Love MI, Huber W, Anders S. Moderated estimation of fold change and dispersion for RNA-seq data with DESeq2. Genome Biol. 2014;15:550. 10.1186/s13059-014-0550-8. 25516281 PMC4302049

[57] Langfelder P, Horvath S. WGCNA: an R package for weighted correlation network analysis. BMC Bioinf. 2008;9:559. 10.1186/1471-2105-9-559. PMC2631488 19114008

[58] Shannon P, Markiel A, Ozier O et al. Cytoscape: a software environment for integrated models of biomolecular interaction networks. Genome Res. 2003;13:2498–504. 10.1101/gr.1239303. 14597658 PMC403769

[59] Livak KJ, Schmittgen TD. Analysis of relative gene expression data using real-time quantitative PCR and the 2-ΔΔCT method. Methods. 2001;25:402–8. 10.1006/meth.2001.1262. 11846609

[60] Tohge T, Fernie AR. Combining genetic diversity, informatics and metabolomics to facilitate annotation of plant gene function. Nat Protoc. 2010;5:1210–27. 10.1038/nprot.2010.82. 20539294

[61] Chen C, Chen H, Zhang Y et al. TBtools: an integrative toolkit developed for interactive analyses of big biological data. Mol Plant. 2020;13:1194–202. 10.1016/j.molp.2020.06.009. 32585190

[62] Bailey TL, Boden M, Buske FA, et al. MEME SUITE: tools for motif discovery and searching. Nucleic Acids Res. 2009;37:W202–8. 10.1093/nar/gkp335. 19458158 PMC2703892

[63] Lescot M . PlantCARE, a database of plant cis-acting regulatory elements and a portal to tools for in silico analysis of promoter sequences. Nucleic Acids Res. 2002;30:325–27. 10.1093/nar/30.1.325. 11752327 PMC99092

[64] Jung Y, Han D. BWA-MEME: BWA-MEM emulated with a machine learning approach. Bioinformatics. 2022;38:2404–13. 10.1093/bioinformatics/btac137. 35253835

[65] Rasmussen SK, Avato P. Characterization of chromosomes and genome organization of Thapsia garganica L. by localizations of rRNA genes using fluorescent in situ hybridization. Hereditas. 1998;129:231–39. 10.1111/j.1601-5223.1998.t01-1-00231.x. 10319718

[66] Weitzel C, Rønsted N, Spalik K et al. Resurrecting deadly carrots: towards a revision of *Thapsia* (Apiaceae) based on phylogenetic analysis of nrITS sequences and chemical profiles. Bot J Linn Soc. 2014;174:620–36. 10.1111/boj.12144.

[67] Vogt T . Phenylpropanoid biosynthesis. Mol Plant. 2010;3:2–20. 10.1093/mp/ssp106. 20035037

[68] Karamat F, Olry A, Doerper S et al. CYP98A22, a phenolic ester 3′-hydroxylase specialized in the synthesis of chlorogenic acid, as a new tool for enhancing the furanocoumarin concentration in Ruta graveolens. BMC Plant Biol. 2012;12:152. 10.1186/1471-2229-12-152. 22931486 PMC3493272

[69] Kai K, Mizutani M, Kawamura N, et al. Scopoletin is biosynthesized via *ortho*-hydroxylation of feruloyl CoA by a 2-oxoglutarate-dependent dioxygenase in *Arabidopsis thaliana*. Plant J. 2008;55:989–99. 10.1111/j.1365-313X.2008.03568.x. 18547395

[70] Vialart G, Hehn A, Olry A et al. A 2-oxoglutarate-dependent dioxygenase from Ruta graveolens L. exhibits p-coumaroyl CoA 2′-hydroxylase activity (C2′H): a missing step in the synthesis of umbelliferone in plants. Plant J. 2012;70:460–70. 10.1111/j.1365-313X.2011.04879.x. 22168819

[71] Matsumoto S, Mizutani M, Sakata K, et al. Molecular cloning and functional analysis of the ortho-hydroxylases of p-coumaroyl coenzyme A/feruloyl coenzyme A involved in formation of umbelliferone and scopoletin in sweet potato, ipomoea batatas (L.) Lam. Phytochemistry. 2012;74:49–57. 10.1016/j.phytochem.2011.11.009. 22169019

[72] Vanholme R, Sundin L, Seetso KC et al. COSY catalyses trans–cis isomerization and lactonization in the biosynthesis of coumarins. Nat Plants. 2019;5:1066–75. 10.1038/s41477-019-0510-0. 31501530

[73] Mao L, Kawaide H, Higuchi T, et al. Genomic evidence for convergent evolution of gene clusters for momilactone biosynthesis in land plants. Proc Natl Acad Sci USA. 2020;117:12472–80. 10.1073/pnas.1914373117. 32409606 PMC7275736

[74] Wu YS, Hillwig ML, Wang Q, et al. Parsing a multifunctional biosynthetic gene cluster from rice: biochemical characterization of CYP71Z6 & 7. FEBS Lett. 2011.; 585:3446–3451. 10.1016/j.febslet.2011.09.038. 21985968 PMC3227696

[75] Wang Q, Hillwig ML, Okada K et al. Characterization of CYP76M5–8 indicates metabolic plasticity within a plant biosynthetic gene cluster. J Biol Chem. 2012;287:6159–68. 10.1074/jbc.M111.305599. 22215681 PMC3307284

[76] Bryson AE, Lanier ER, Lau KH et al. Uncovering a miltiradiene biosynthetic gene cluster in the Lamiaceae reveals a dynamic evolutionary trajectory. Nat Commun. 2023;14:343. 10.1038/s41467-023-35845-1. 36670101 PMC9860074

[77] Takos AM, Knudsen C, Lai D et al. Genomic clustering of cyanogenic glucoside biosynthetic genes aids their identification in *Lotus japonicus* and suggests the repeated evolution of this chemical defence pathway. Plant J. 2011;68:273–86. 10.1111/j.1365-313X.2011.04685.x. 21707799

[78] Soler-Oliva ME, Guerrero-Martínez JA, Bachetti V, et al. Analysis of the relationship between coexpression domains and chromatin 3D organization. PLoS Comput Biol. 2017;13:e1005708 10.1371/journal.pcbi.1005708. 28902867 PMC5612749

[79] Nelson D, Werck-Reichhart D. A P450-centric view of plant evolution. Plant J. 2011;66:194–211. 10.1111/j.1365-313X.2011.04529.x. 21443632

[80] Weitzel C, Simonsen HT. Cytochrome P450-enzymes involved in the biosynthesis of mono- and sesquiterpenes. Phytochem Rev. 2015;14:7–24. 10.1007/s11101-013-9280-x.

[81] Hamberger B, Bak S. Plant P450s as versatile drivers for evolution of species-specific chemical diversity. Phil Trans R Soc B. 2013;368:20120426 10.1098/rstb.2012.0426. 23297350 PMC3538417

[82] Hamerski D, Schmitt D, Matern U. Induction of two prenyltransferases for the accumulation of coumarin phytoalexins in elicitor-treated Ammi majus cell suspension cultures. Phytochemistry. 1990;29:1131–35. 10.1016/0031-9422(90)85417-E. 1366425

[83] Li M-Y, Feng K, Hou X-L et al. The genome sequence of celery (Apium graveolens L.), an important leaf vegetable crop rich in apigenin in the Apiaceae family. Hortic Res. 2020;7:1–10. 10.1038/s41438-019-0235-2. 31934340 PMC6944684

[84] Krieger C, Kamo T, Bourgaud F et al. Evolution of substrate recognition sites (SRSs) in cytochromes P450 from Apiaceae exemplified by the CYP71AJ subfamily. BMC Evol Biol. 2015;15:1–14. 10.1186/s12862-015-0396-z. 26111527 PMC4482195

[85] Xu W, Dubos C, Lepiniec L. Transcriptional control of flavonoid biosynthesis by MYB–bHLH–WDR complexes. Trends Plant Sci. 2015;20:176–85. 10.1016/j.tplants.2014.12.001. 25577424

[86] Sun B, Zhu Z, Cao P, et al. Purple foliage coloration in tea (Camellia sinensis L.) arises from activation of the R2R3-MYB transcription factor CsAN1. Sci Rep. 2016;6:32534. 10.1038/srep32534. 27581206 PMC5007479

[87] Döll S, Kuhlmann M, Rutten T, et al. Accumulation of the coumarin scopolin under abiotic stress conditions is mediated by the *Arabidopsis thaliana* THO/TREX complex. Plant J. 2018;93:431–44. 10.1111/tpj.13797. 29222952

[88] Simonsen HT, Weitzel C, Christensen SB. Guaianolide sesquiterpenoids: pharmacology and biosynthesis. In: Ramawat KG, Merillon JM, eds. Natural Products: Phytochemistry, Botany and Metabolism of Alkaloids, Phenolics and Terpenes. Berlin: Springer-Verlag; 2013.

[89] Christensen SB, Simonsen HT, Engedal N, et al. From plant to patient: thapsigargin, a tool for understanding natural product chemistry, total syntheses, biosynthesis, taxonomy, ATPases, cell death, and drug development. In: Kinghorn AD, Falk H, Gibbons S, Asakawa Y, Liu J-K, Dirsch VM, eds. Progress in the Chemistry of Organic Natural Products 115. Cham: Springer International Publishing; 2021. 10.1007/978-3-030-64853-4. 33797641

[90] Drew DP, Krichau N, Reichwald K et al. Guaianolides in Apiaceae: perspectives on pharmacology and biosynthesis. Phytochem Rev. 2009;8:581–99. 10.1007/s11101-009-9130-z.

[91] Chen F, Tholl D, Bohlmann J, et al. The family of terpene synthases in plants: a mid-size family of genes for specialized metabolism that is highly diversified throughout the kingdom. Plant J. 2011;66:212–29. 10.1111/j.1365-313X.2011.04520.x. 21443633

[92] Song C, Li X, Jia B, et al. Comparative transcriptomics unveil the crucial genes involved in coumarin biosynthesis in *Peucedanum praeruptorum* Dunn. Front Plant Sci. 2022;13:1–14. 10.3389/fpls.2022.899819. PMC9152428 35656010

[93] Liao B, Shen X, Xiang L et al. Allele-aware chromosome-level genome assembly of Artemisia annua reveals the correlation between ADS expansion and artemisinin yield. Mol Plant. 2022;15:1310–28. 10.1016/j.molp.2022.05.013. 35655434

[94] Liu JX, Liu H, Tao JP et al. High-quality genome sequence reveals a young polyploidization and provides insights into cellulose and lignin biosynthesis in water dropwort (*Oenanthe sinensis*). Ind Crops Prod. 2023;193:116203. 10.1016/j.indcrop.2022.116203.

[95] Nelson DR . Cytochrome P450 diversity in the tree of life. Biochim Biophys Acta Proteins Proteom. 2018;1866:141–54. 10.1016/j.bbapap.2017.05.003. 28502748 PMC5681887

[96] Mizutani M, Ohta D. Diversification of P450 genes during land plant evolution. Annu Rev Plant Biol. 2010;61:291–315. 10.1146/annurev-arplant-042809-112305. 20192745

[97] Hamerski D, Matern U. Elicitor-induced biosynthesis of psoralens in *Ammi majus* L. suspension cultures. Eur J Biochem. 1988;171:369–75. 10.1111/j.1432-1033.1988.tb13800.x. 2828055

[98] Jian X, Zhao Y, Wang Z, et al. Two CYP71AJ enzymes function as psoralen synthase and angelicin synthase in the biosynthesis of furanocoumarins in *Peucedanum praeruptorum* Dunn. Plant Mol Biol. 2020;104:327–37. 10.1007/s11103-020-01045-4. 32761540

[99] Roselli S, Olry A, Vautrin S, et al. A bacterial artificial chromosome (BAC) genomic approach reveals partial clustering of the furanocoumarin pathway genes in parsnip. Plant J. 2017;89:1119–32. 10.1111/tpj.13450. 27943460

[100] Bai M . Genome and gene of Peucedanum praeruptorum. Figshare Dataset. 2024-04-25. 10.6084/m9.figshare.25249453.v1.

[101] Bai M, Jiang S, Chu S et al. Supporting data for “The Telomere-to-Telomere (T2T) Genome of *Peucedanum praeruptorum* Dunn Provides Insights into the Genome Evolution and Coumarin Biosynthesis.”. GigaScience Database. 2024. 10.5524/102520. PMC11152176 38837945

