# Supplementary material for: The telomere-to-telomere (T2T) genome of Peucedanum praeruptorum Dunn provides insights into the genome evolution and coumarin biosynthesis
Source: Gigascience. 2024 Jun 5;13:giae025. doi: 10.1093/gigascience/giae025 (PMC11152176; doi:10.1093/gigascience/giae025)

Supplementary materials

[Figure S1: The K-mer depth distribution for *Peucedanum praeruptorum* Dunn genome size evaluation. 2](#_Toc158209118)

[Figure S2: The pipeline for the assembly of the T2T genome of *Peucedanum praeruptorum* Dunn. 2](#_Toc158209119)

[Figure S3: Statistical graph of correlation analysis between GC content and Depth (short reads and long reads). 3](#_Toc158209120)

[Figure S4: The Gene structure prediction results and gene set statistics (comparison with gene elements of closely related species). 3](#_Toc158209121)

[Figure S5: The cumulative distribution statistics graph of gene set element length (comparison with gene elements of closely related species). 4](#_Toc158209122)

[Figure S6: Venn diagram of homologous gene families of *Peucedanum praeruptorum* Dunn in the genome. 4](#_Toc158209123)

[Figure S7: GO enrichment annotation of *Peucedanum praeruptorum* Dunn. 5](#_Toc158209124)

[Figure S8: Kyoto Encyclopedia of Genes and Genomes (KEGG) enrichment annotation of *Peucedanum praeruptorum* Dunn. 5](#_Toc158209125)

[Figure S9: Venn diagram of functional annotation of *Peucedanum praeruptorum* Dunn in different databases. 6](#_Toc158209126)

[Figure S10: The syntenic comparison with the newly published genome of *Peucedanum praeruptorum* Dunn. 7](#_Toc158209127)

[Figure S11: The phylogenetic tree of the 11 species genomes with 489 single copy genes. 8](#_Toc158209128)

[Figure S12: GO enrichment results of *Peucedanum praeruptorum* Dunn contraction gene family. 8](#_Toc158209129)

[Figure S13: KEGG enrichment results of *Peucedanum praeruptorum* Dunn contraction gene family. 9](#_Toc158209130)

[Figure S14: GO enrichment results of *Peucedanum praeruptorum* Dunn expansion gene family. 9](#_Toc158209131)

[Figure S15: KEGG enrichment results of *Peucedanum praeruptorum* Dunn expansion gene family. 10](#_Toc158209132)

[Figure S16: The gene syntheny compaction among the *Angelica sinensis, Peucedanum praeruptorum* Dunn and *Daucus carota.* 10](#_Toc158209133)

[Figure S17: Inference of polyploidization and speciation history in Apiaceae. 11](#_Toc158209134)

[Figure S18: Gene family identification and analysis of the terpene biosynthetic pathway. 12](#_Toc158209135)

[Figure S19: Gene family identification and analysis of the coumarin biosynthetic pathway. 13](#_Toc158209136)

[Figure S20: The phylogenetic tree of different species genomes with PT genes. 14](#_Toc158209137)

# Figure S1: The K-mer depth distribution for *Peucedanum praeruptorum* Dunn genome size evaluation.


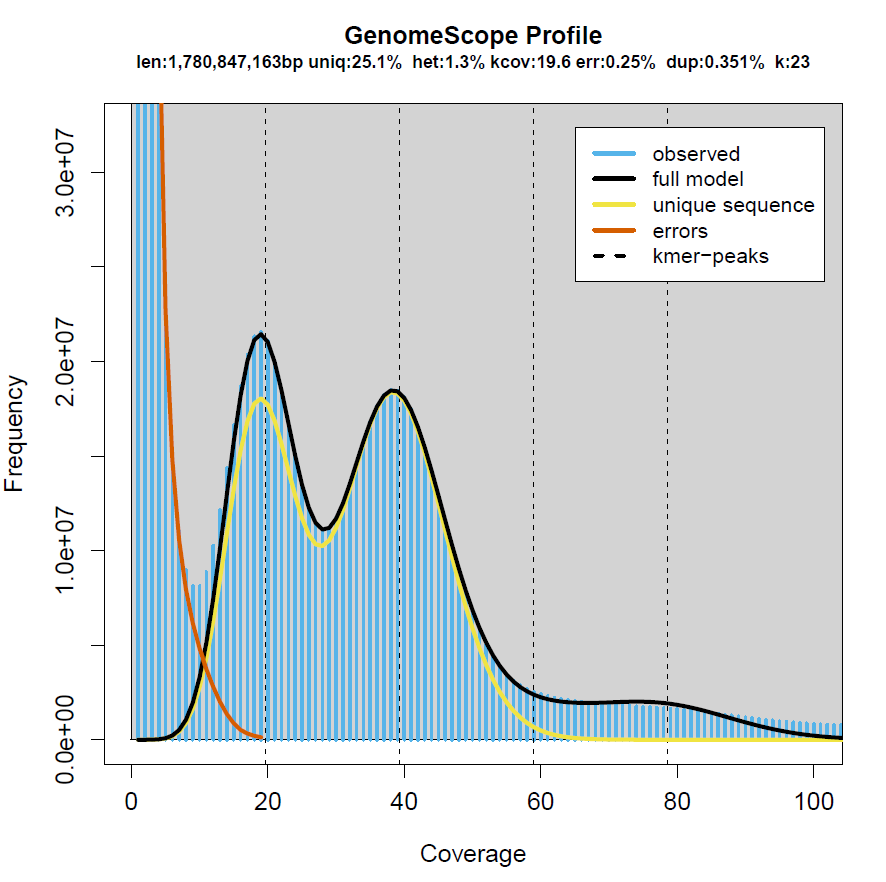


The X-axis is the coverage (X). The y-axis is the frequency of 23-mer.

Note: het: heterozygosity; kcov: kmer mean peak; uniq: non-repetitive kmer; observed: the kmer actual distribution by jellyfish analysis; full model: theoretical kmer distribution; unique sequence: non-repetitive kmer; errors: wrong kmer, usually the lower kmer；kmer-peaks: the position of the kmer peak.

# Figure S2: The pipeline for the assembly of the T2T genome of *Peucedanum praeruptorum* Dunn.


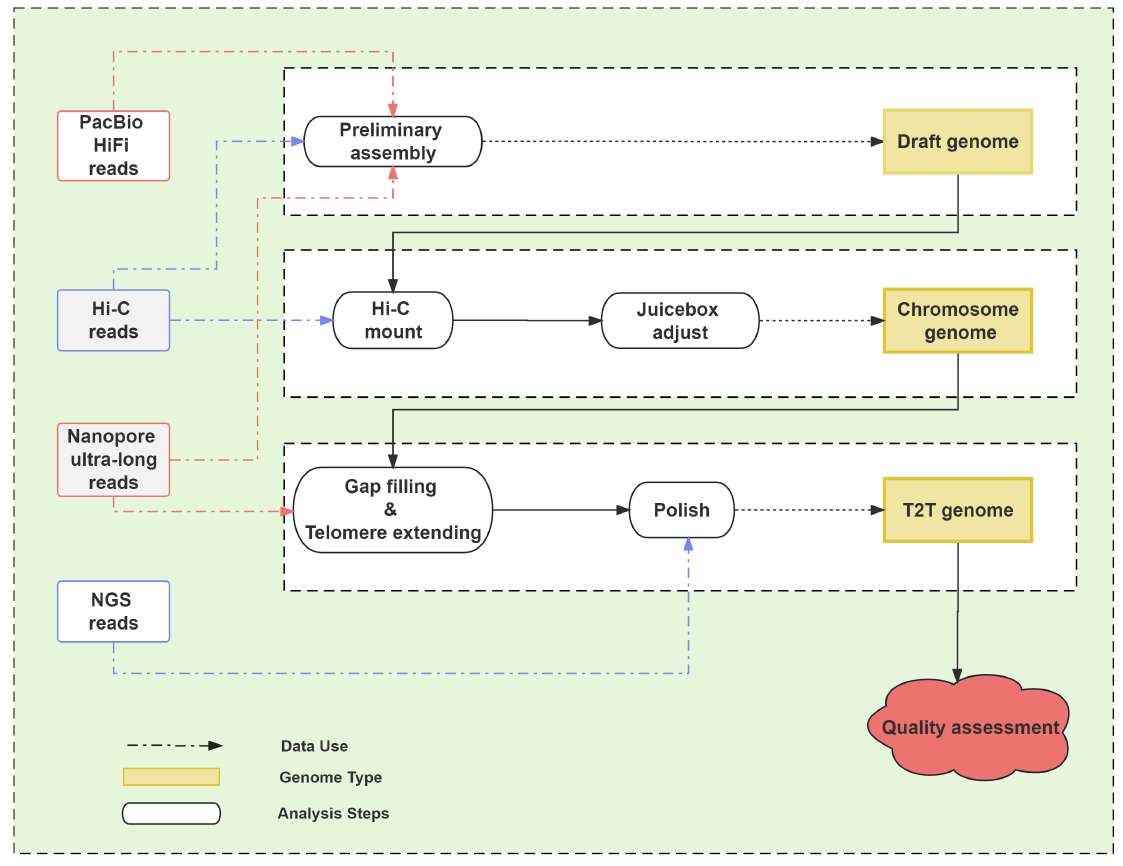


# Figure S3: Statistical graph of correlation analysis between GC content and Depth (short reads and long reads).


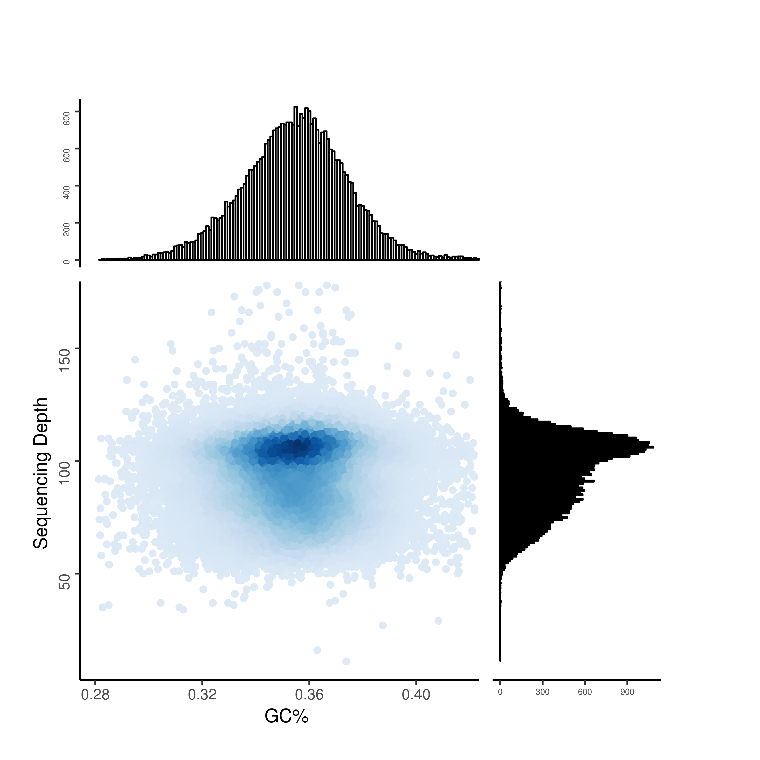

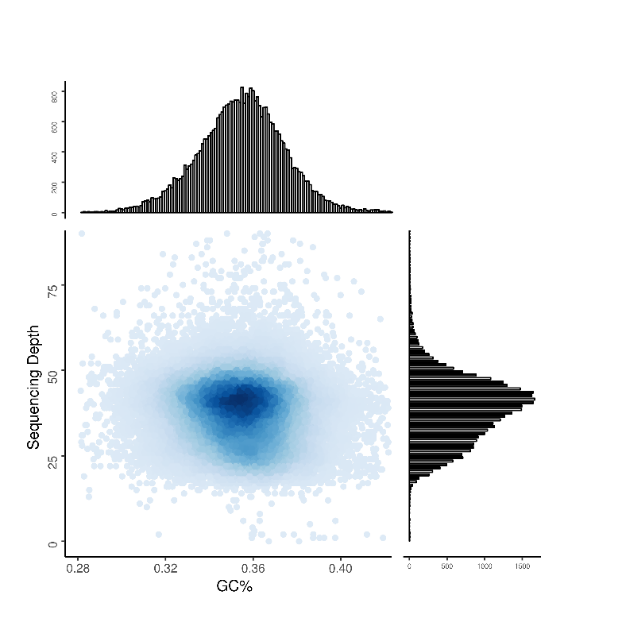


# Figure S4: The Gene structure prediction results and gene set statistics (comparison with gene elements of closely related species).


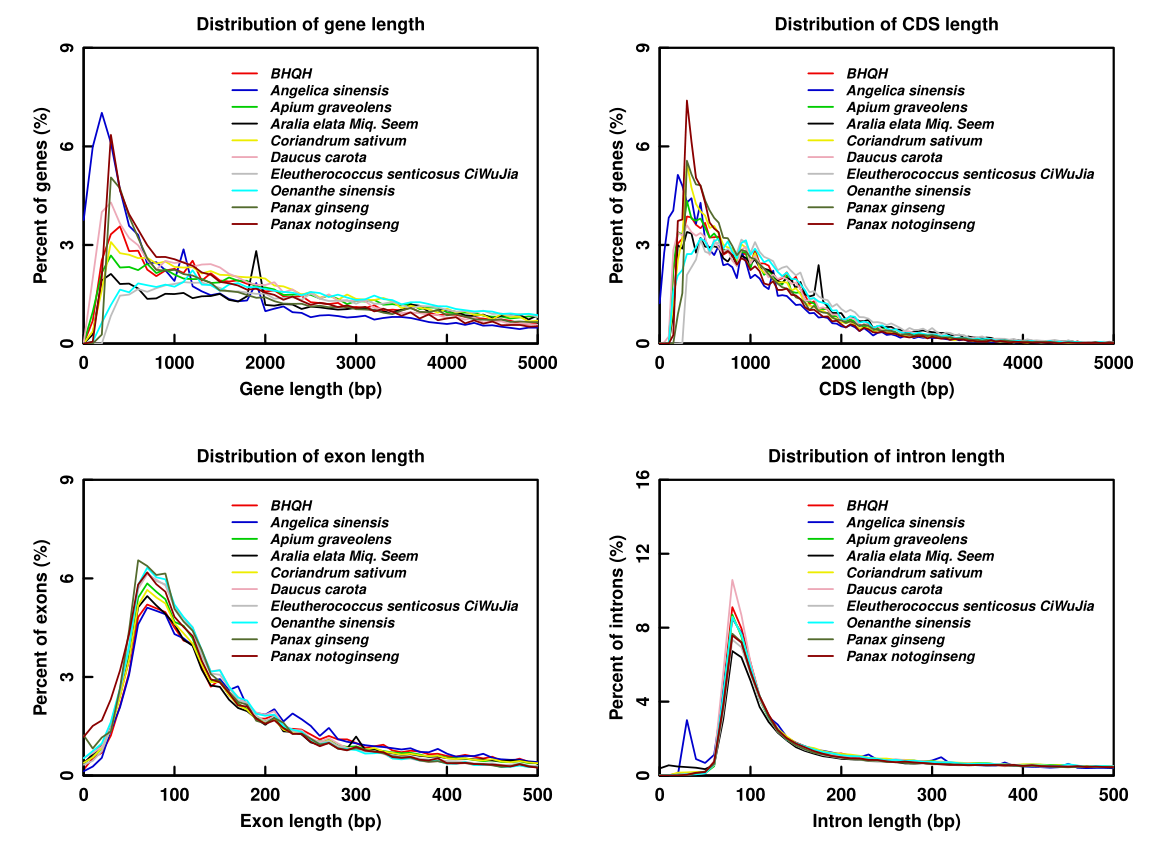


# Figure S5: The cumulative distribution statistics graph of gene set element length (comparison with gene elements of closely related species).

­
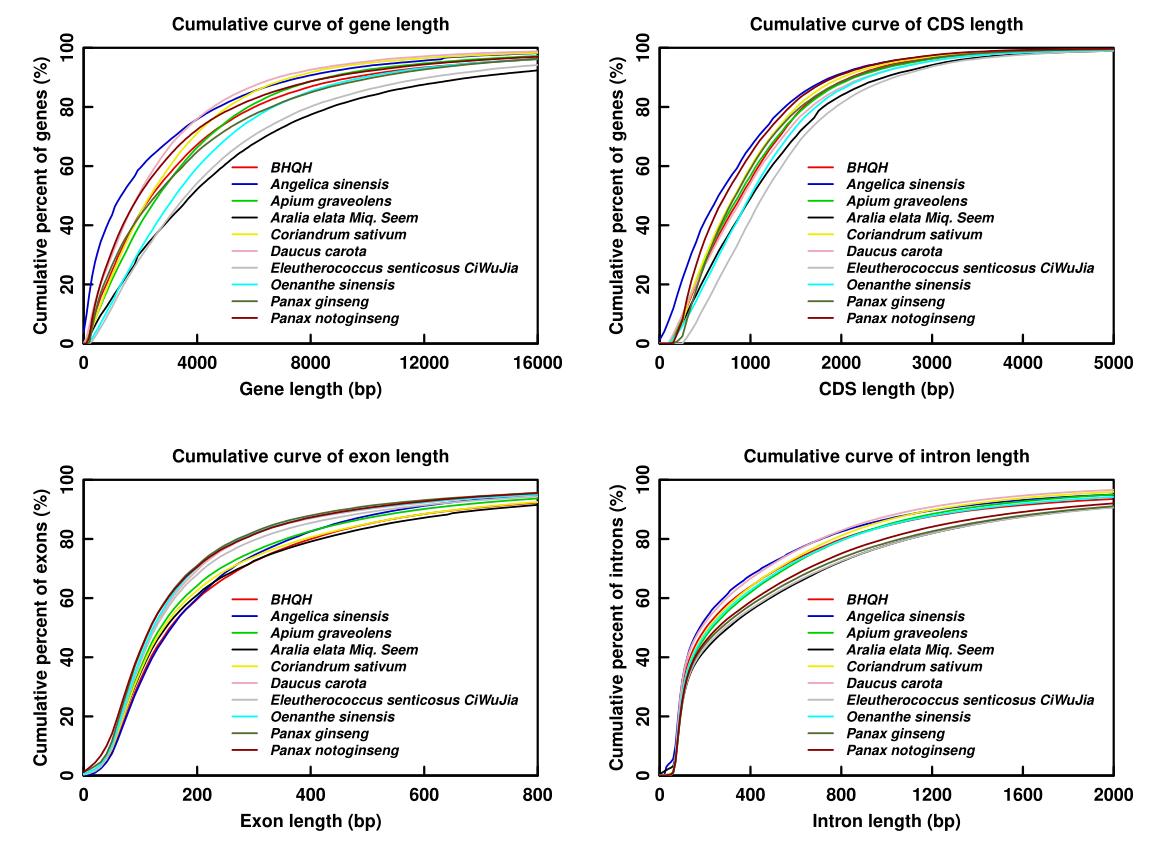


# Figure S
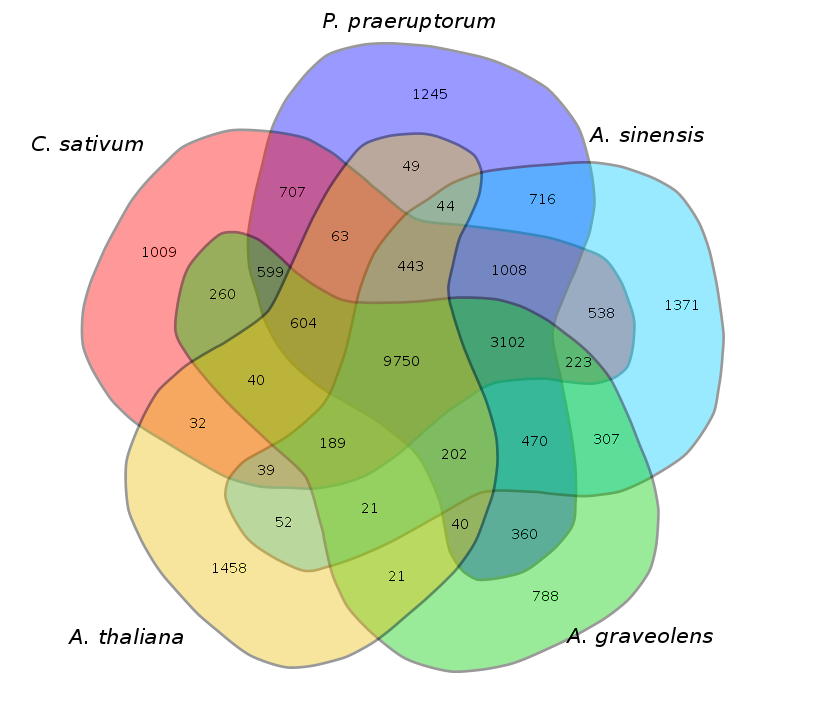
6: Venn diagram of homologous gene families of *Peucedanum praeruptorum* Dunn in the genome.

#
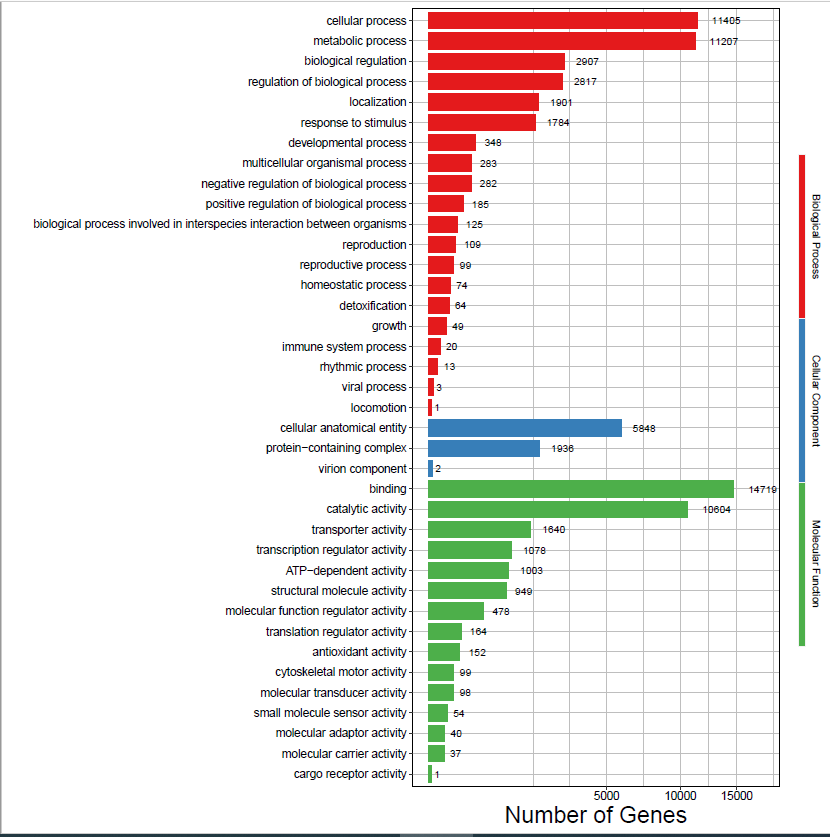
Figure S7: GO enrichment annotation of *Peucedanum praeruptorum* Dunn.

# Figure S8: Kyoto Encyclopedia of Genes and Genomes (KEGG) enrichment annotation of *Peucedanum praeruptorum* Dunn.


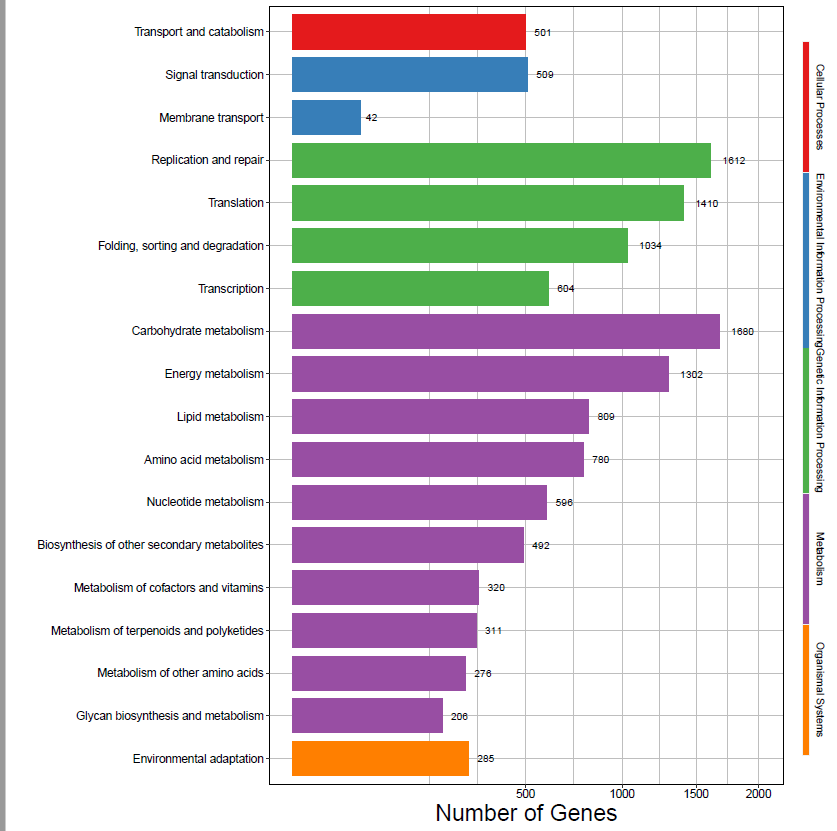


# Figure S9: Venn diagram of functional annotation of *Peucedanum praeruptorum* Dunn in different databases.


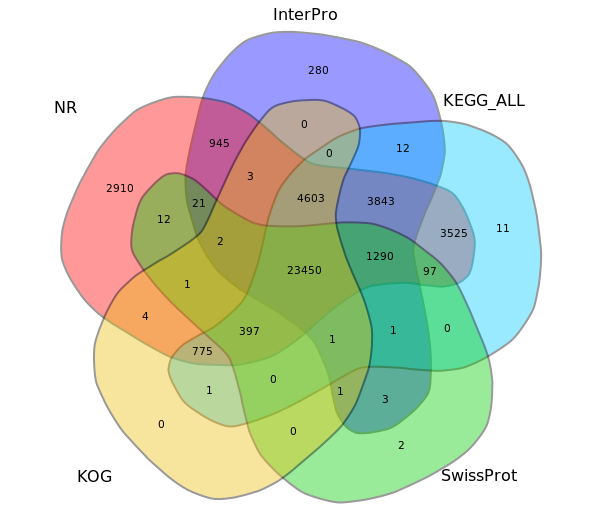


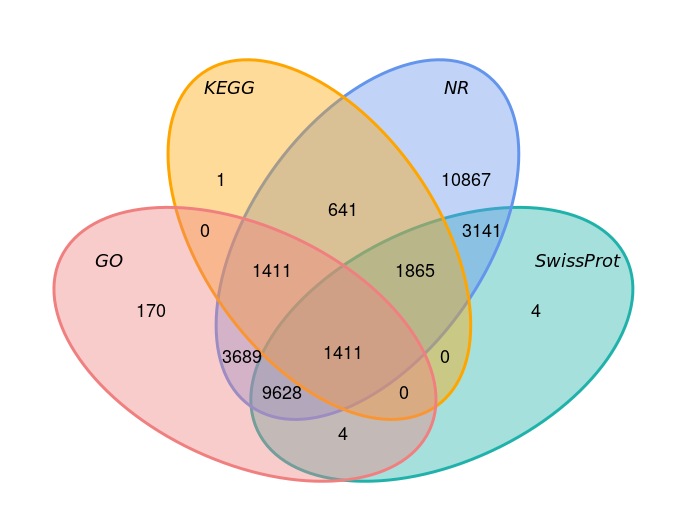


# Figure S10: The syntenic comparison with the newly published genome of *Peucedanum praeruptorum* Dunn.


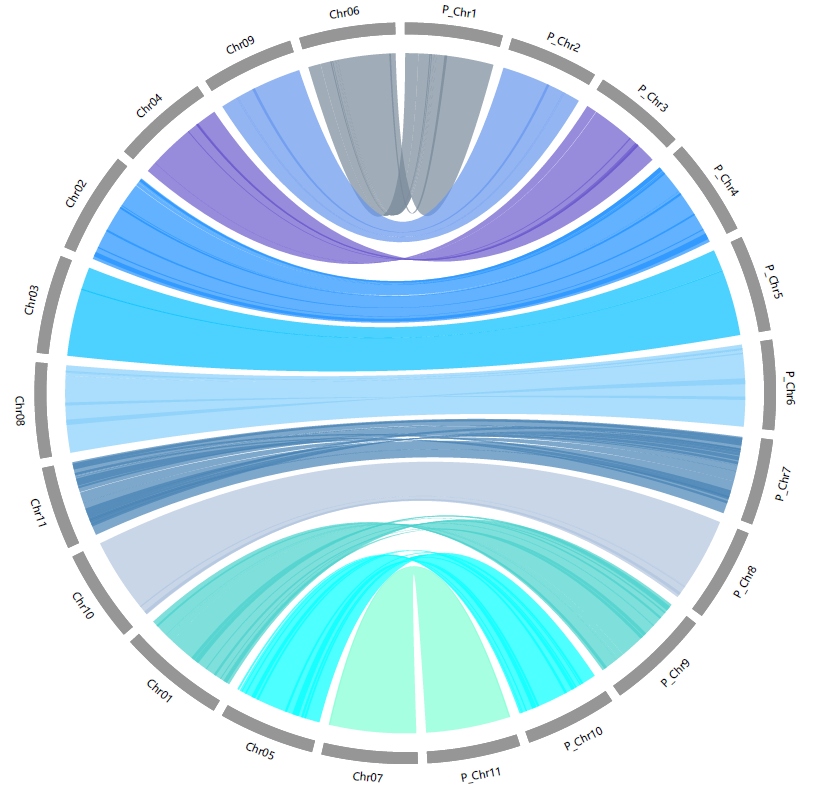


1. Chromosome collinearity analysis with the published genome of *Peucedanum praeruptorum* Dunn (Chen Song et al. 2023)


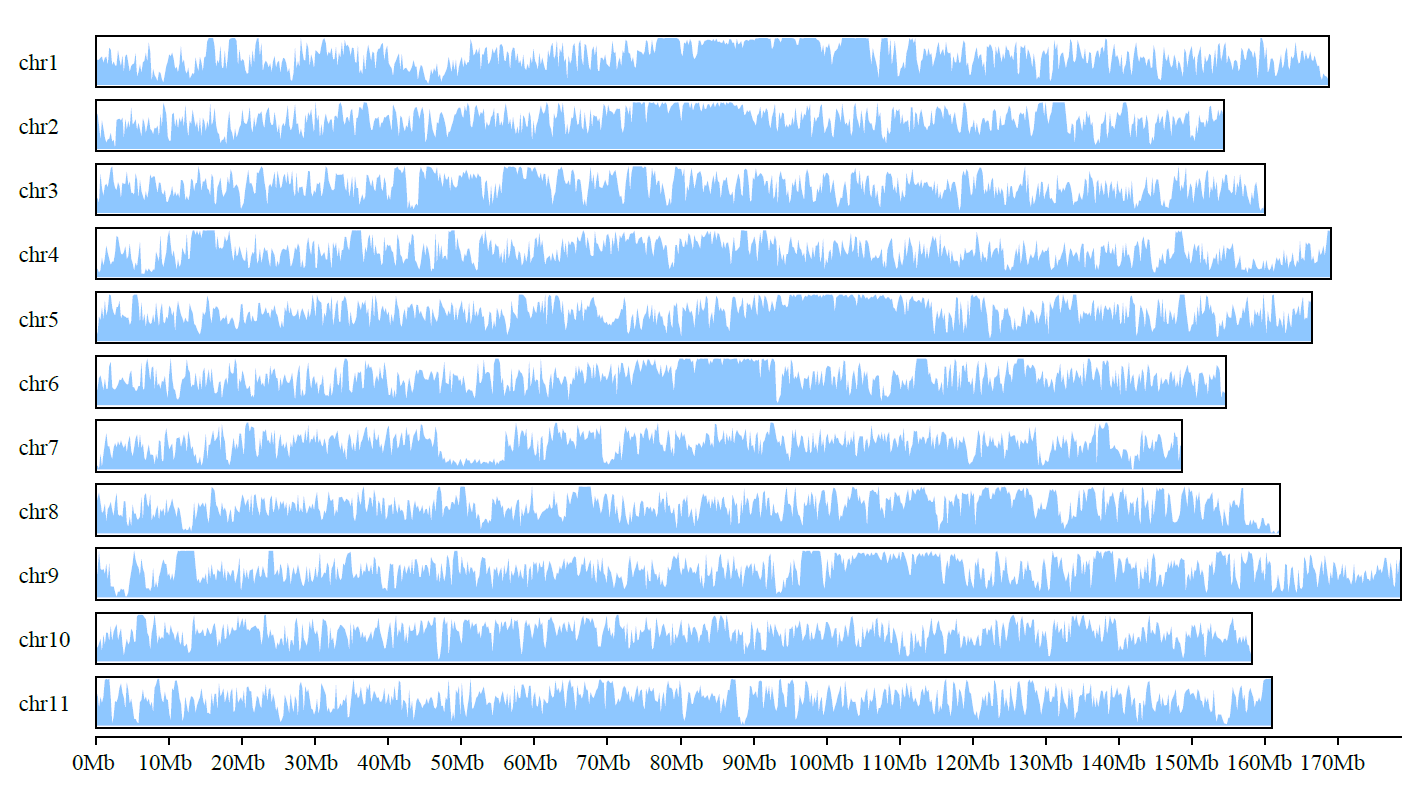


1. Alignment with the published genome of *Peucedanum praeruptorum* Dunn (Chen Song et al. 2023)

# Figure S11: The phylogenetic tree of the 11 species genomes with 489 single copy genes.


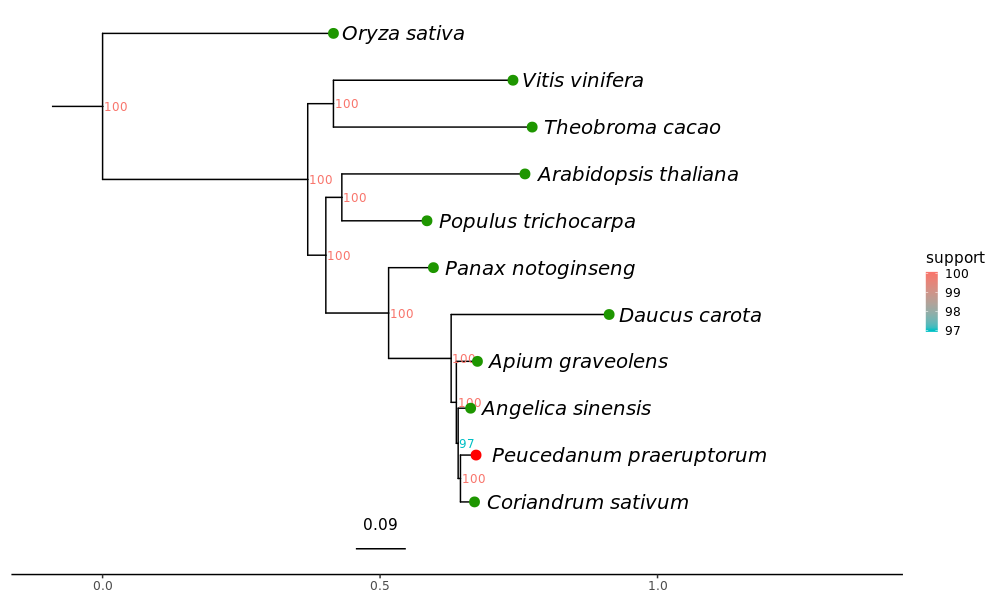


# Figure S12: GO enrichment results of *Peucedanum praeruptorum* Dunn contraction gene family.


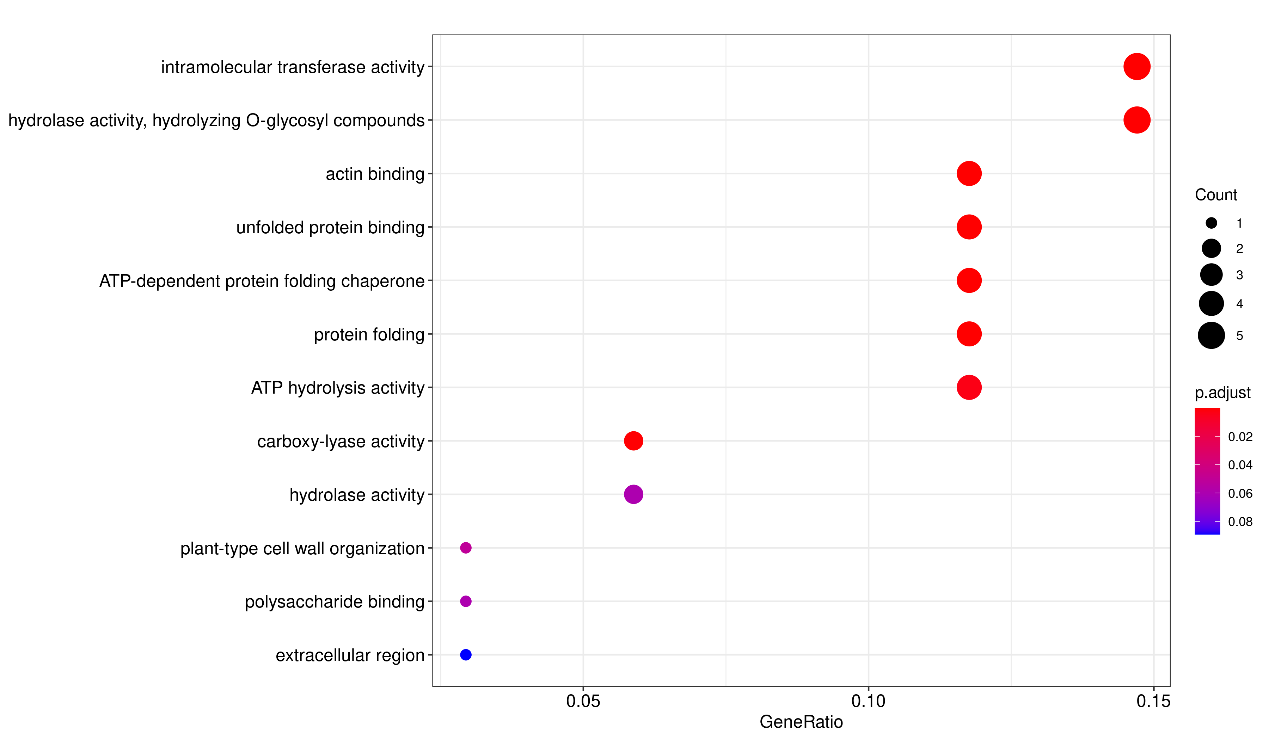


# Figure S13: KEGG enrichment results of *Peucedanum praeruptorum* Dunn contraction gene family.


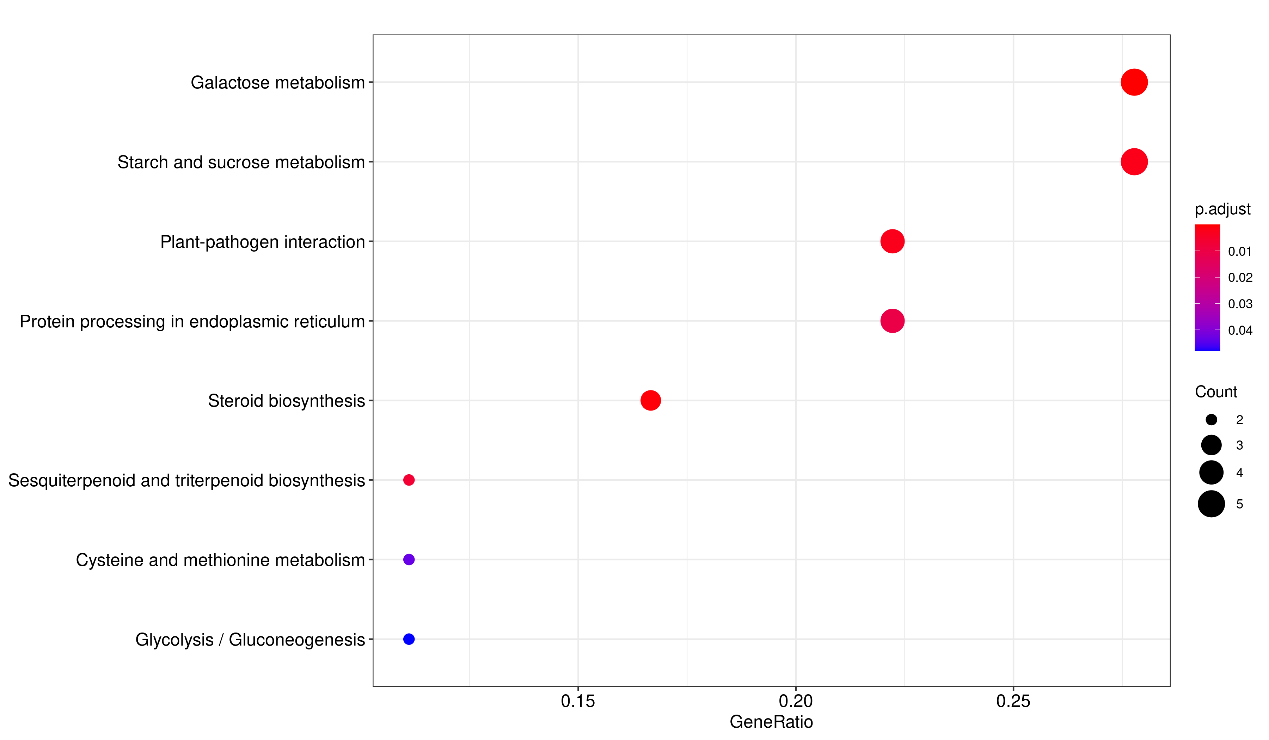


# Figure S14: GO enrichment results of *Peucedanum praeruptorum* Dunn expansion gene family.


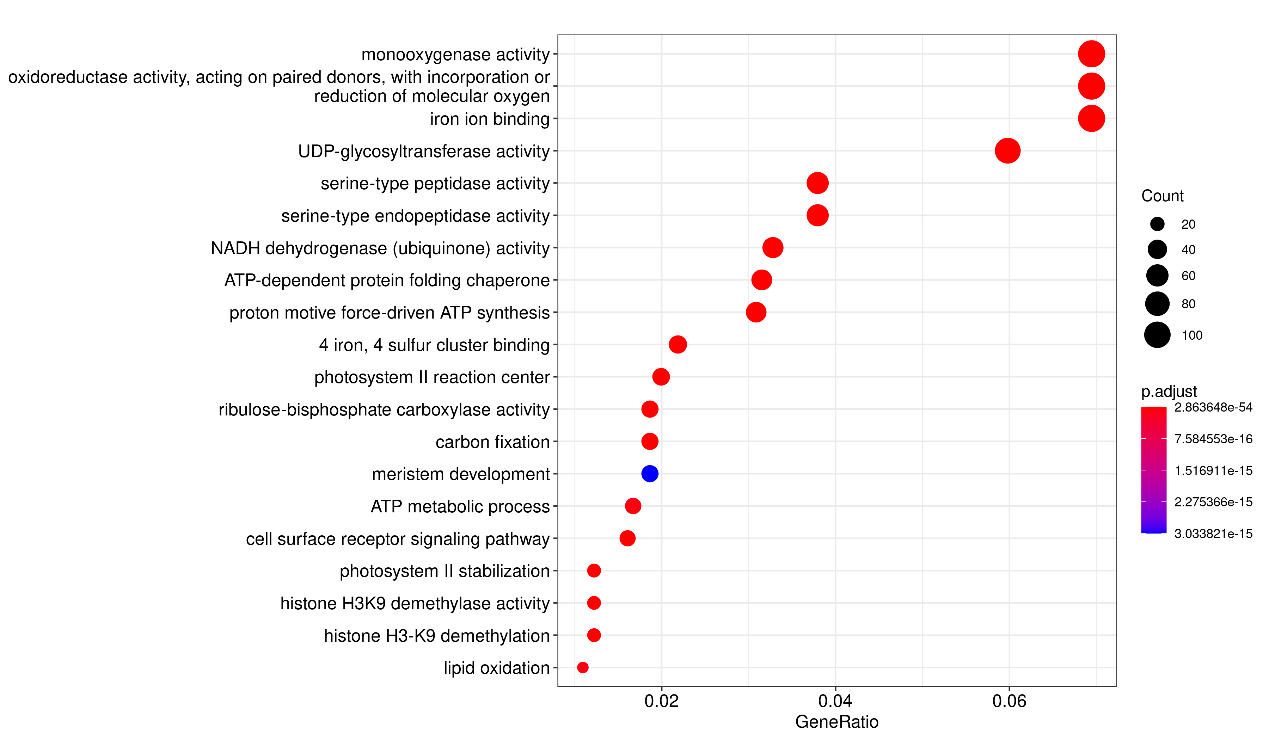


# Figure S15: KEGG enrichment results of *Peucedanum praeruptorum* Dunn expansion gene family.


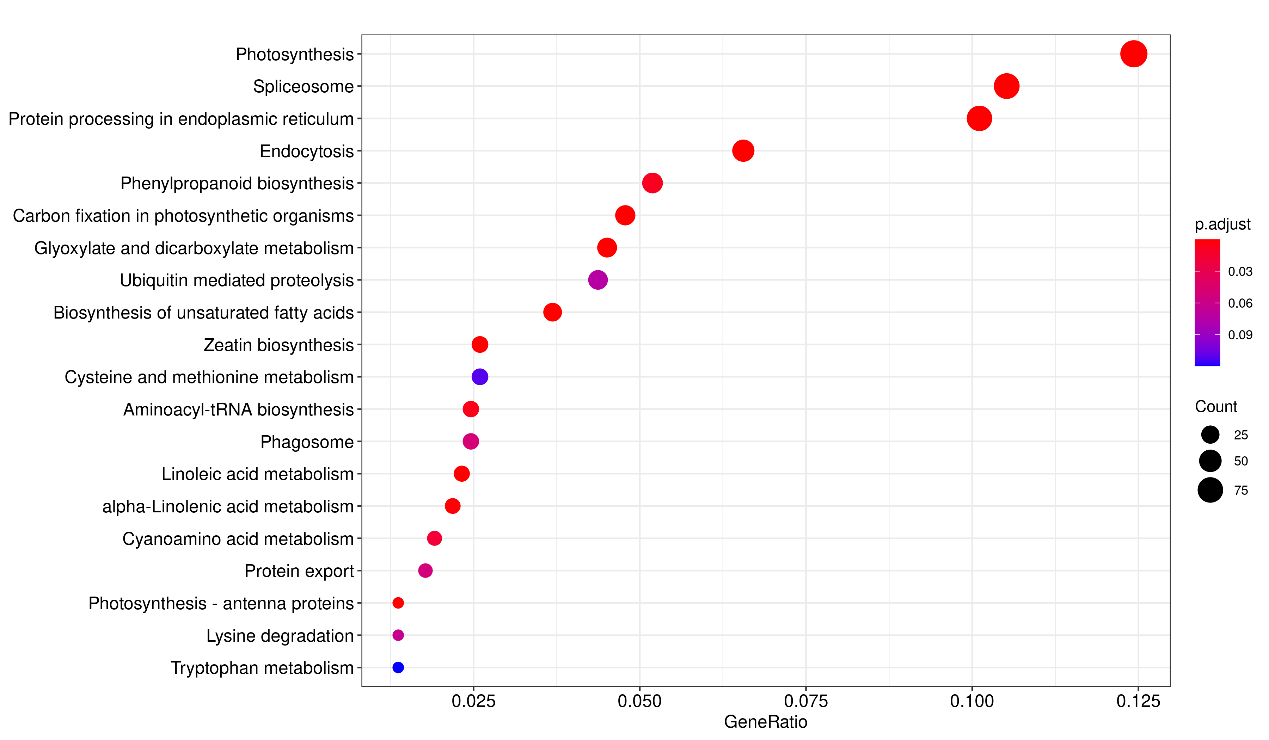


# Figure S16: The gene syntheny compaction among the *Angelica sinensis,* [*Peucedanum praeruptorum* Dunn](javascript:;) and *Daucus carota.*


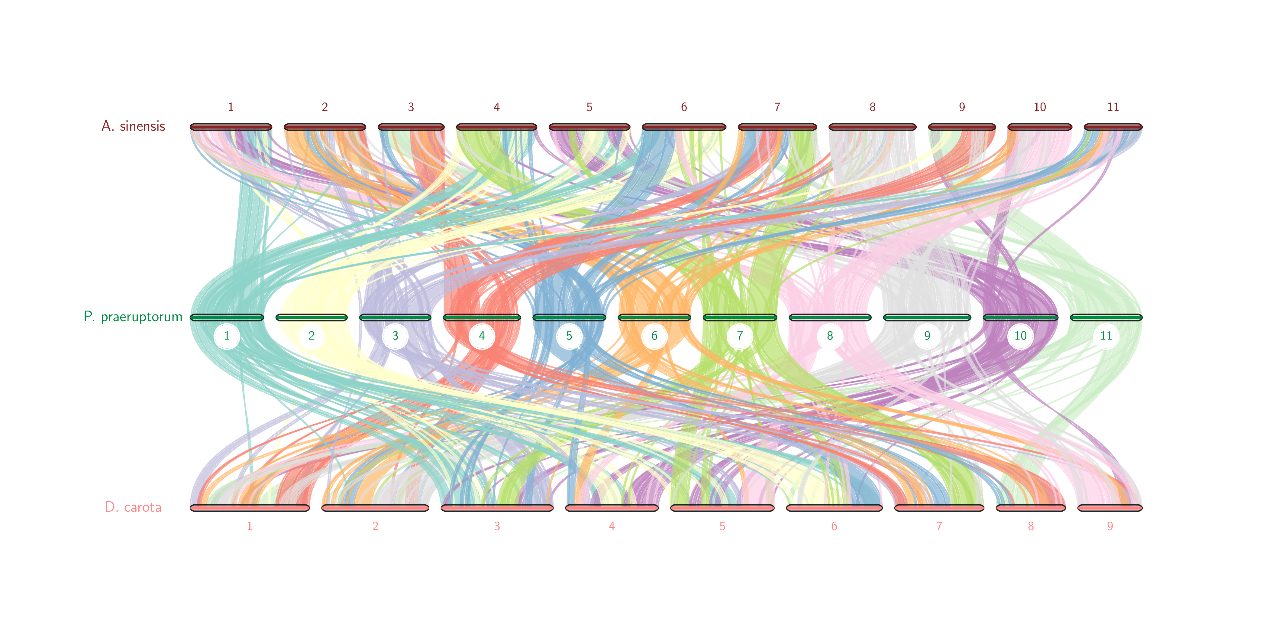


# Figure S17: Inference of polyploidization and speciation history in Apiaceae.


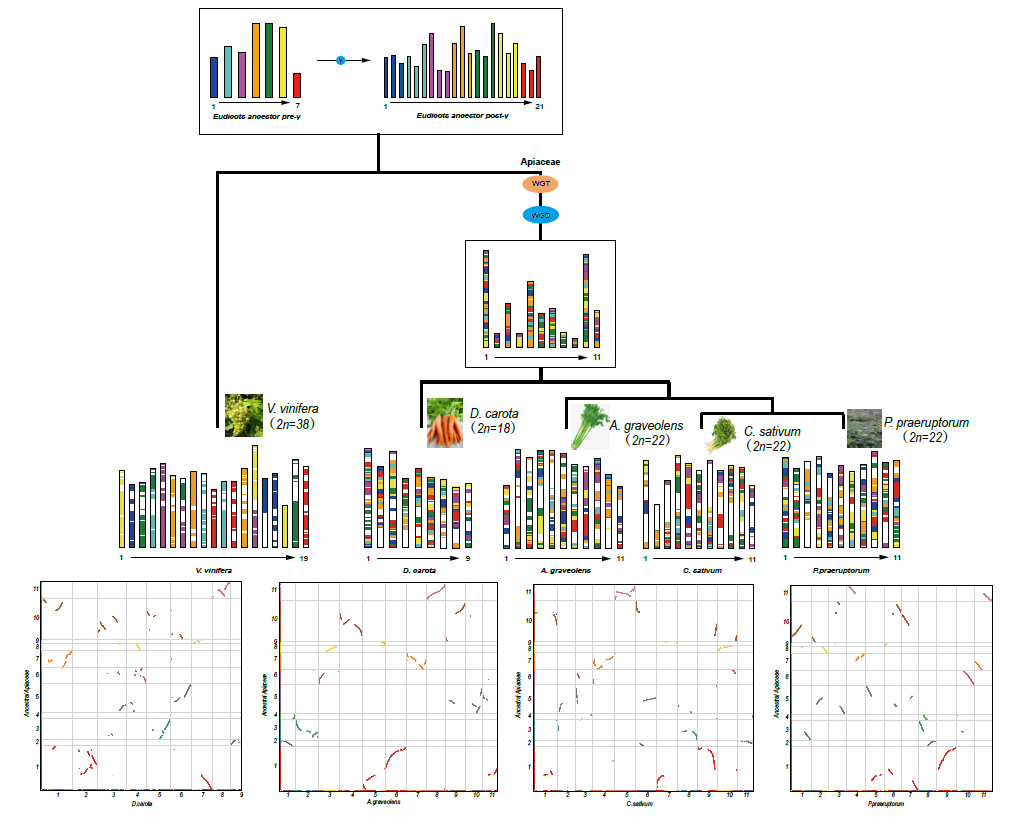


# Figure S18: Gene family identification and analysis of the terpene biosynthetic pathway.

(A) Phylogenetic analysis of Terpen synthase in *Peucedanum praeruptorum*, *Angelica sinensis*, *Daucus carota*, *Populus trichocarpa*, *Arabidopsis thaliana*. (B) Expression profiles of TPS gene family in different tissues. (C) The distribution of Terpenes compouds in the different tissues and three different developmental periods. (D) Distribution of the Terpen synthase genes on P. praeruptorum chromosomes. The scale indicates a megabase (Mb).


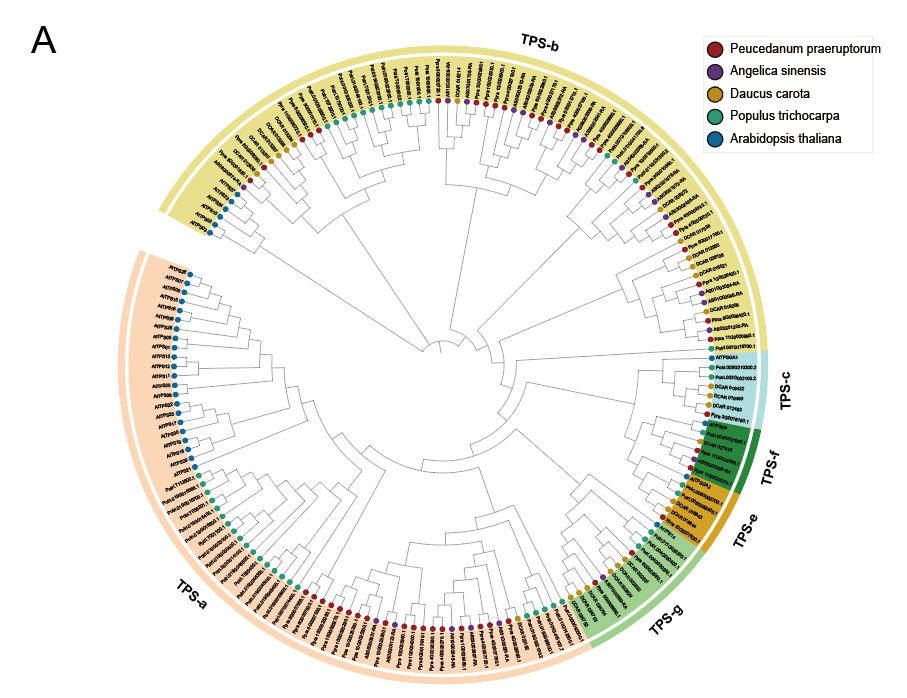


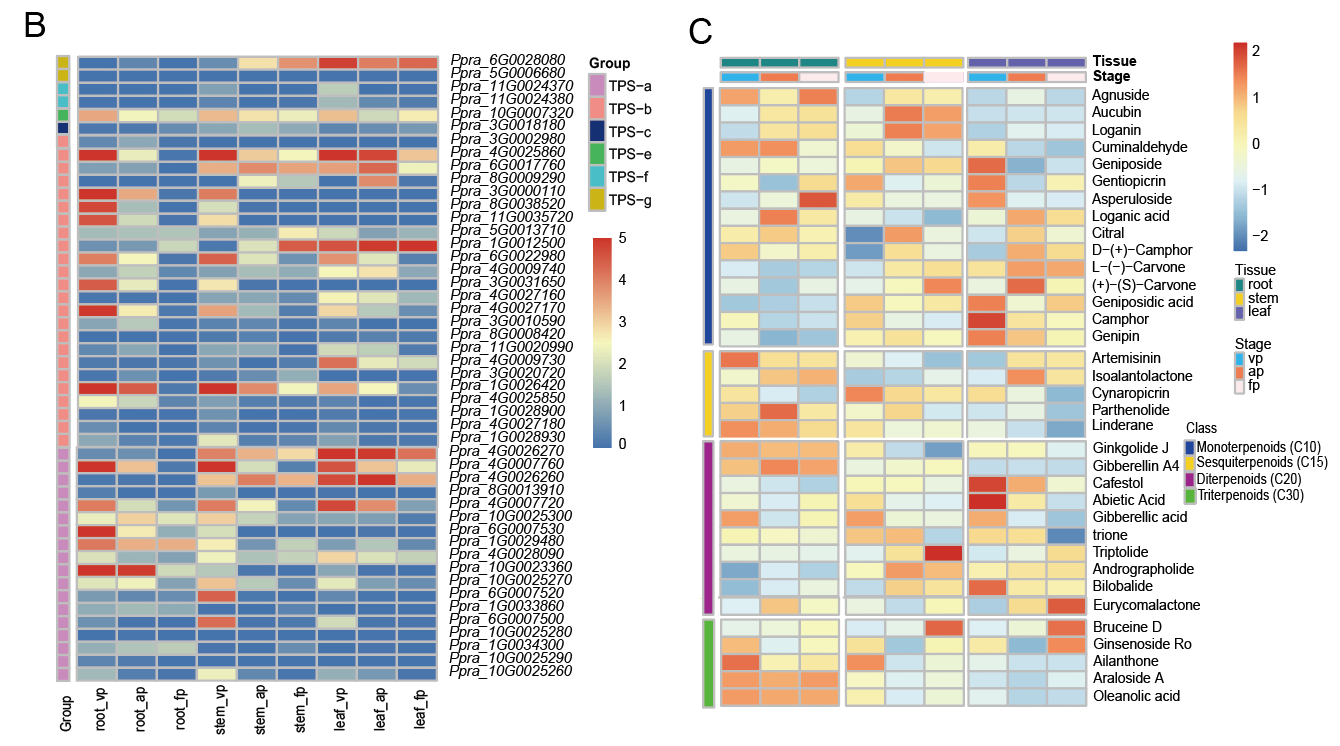


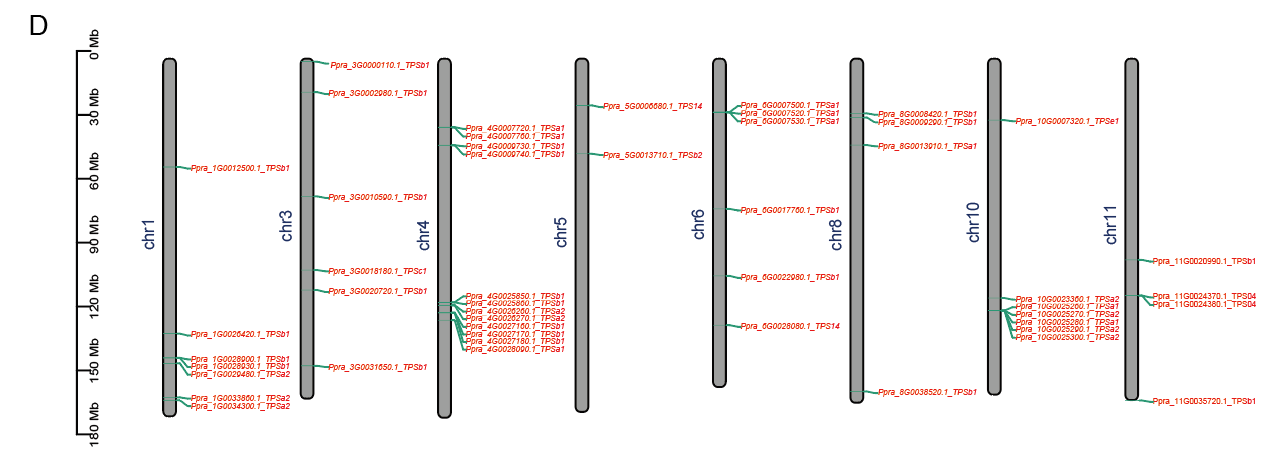


# Figure S19: Gene family identification and analysis of the coumarin biosynthetic pathway.


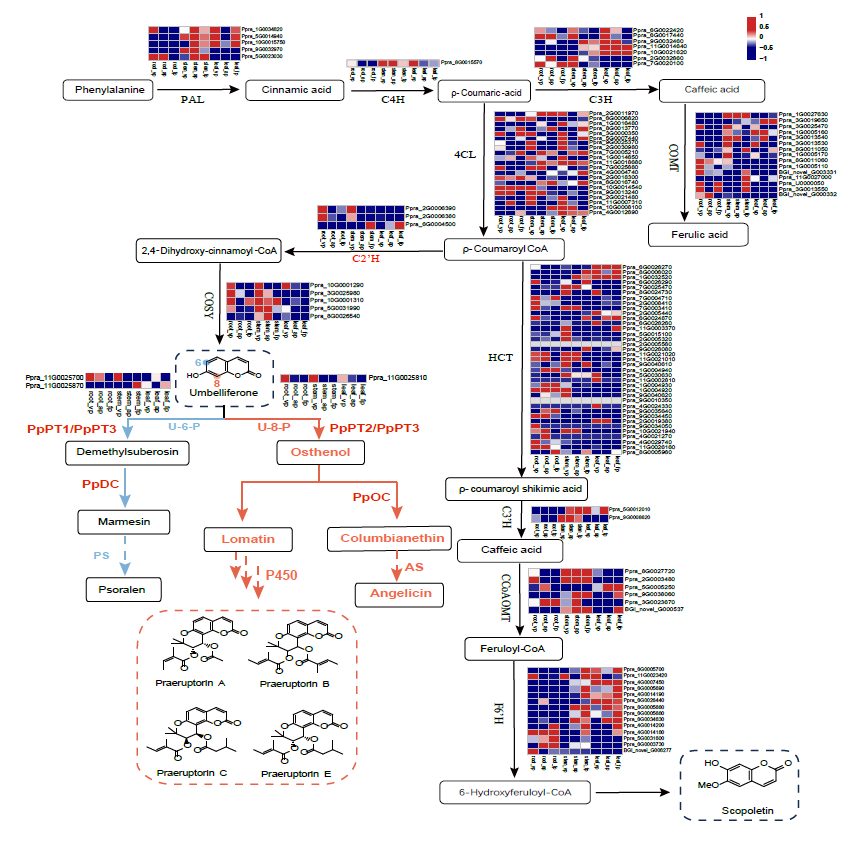
**
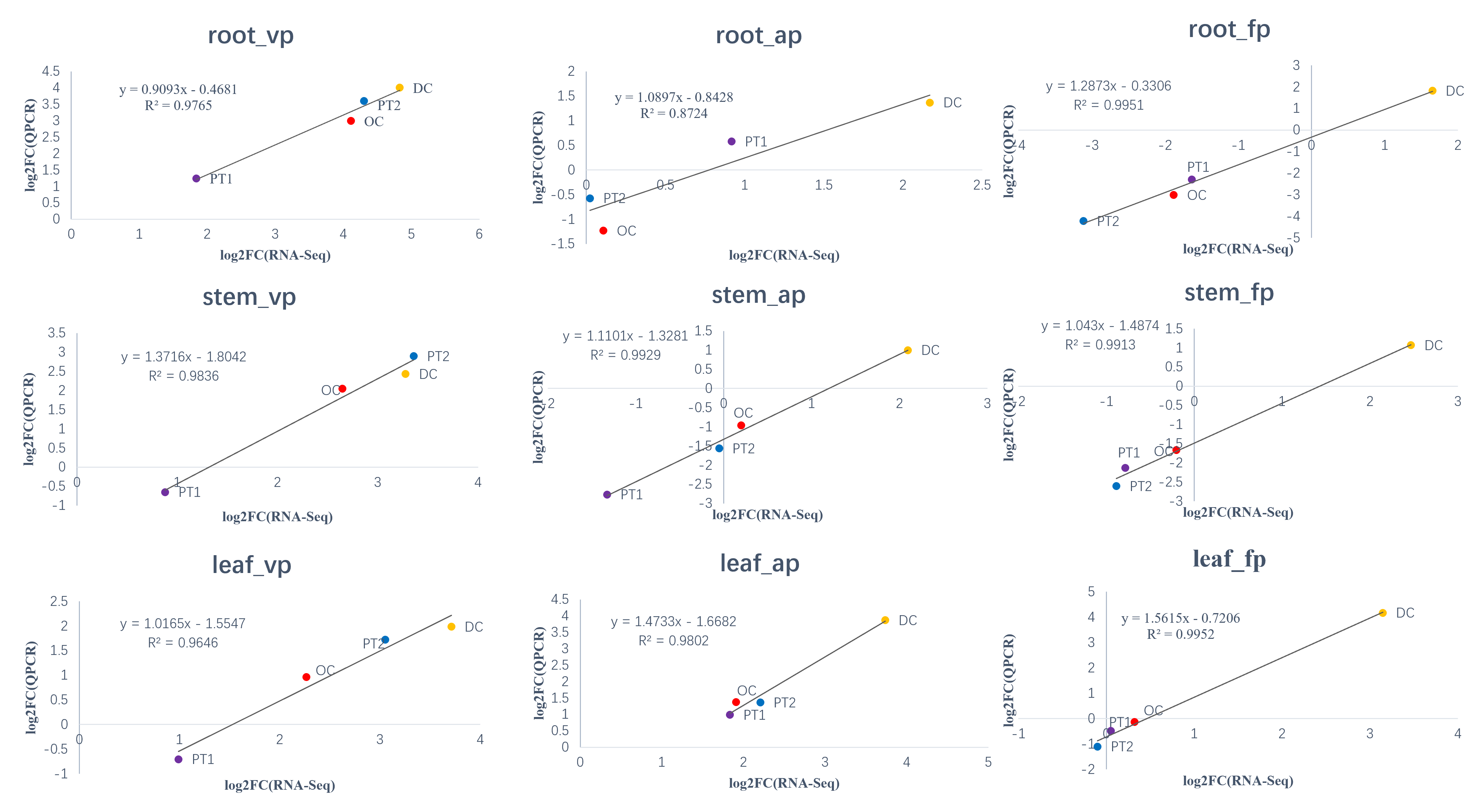
**

# Figure S20: The phylogenetic tree of different species genomes with PT genes.


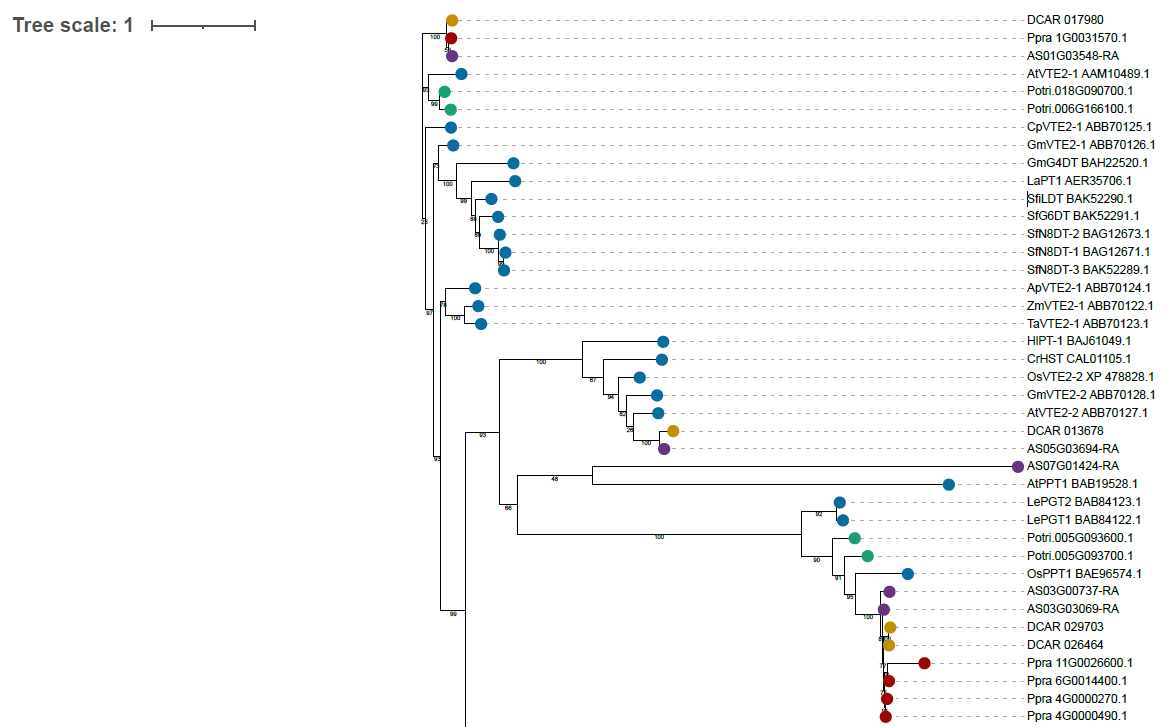

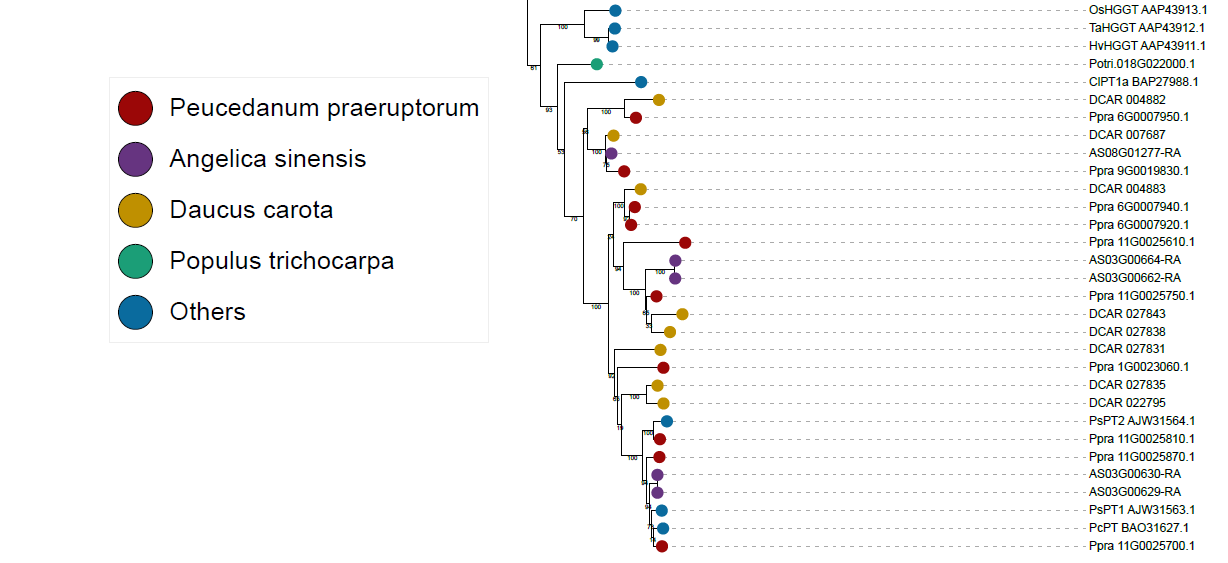

Supplement: giae025_supplement [file giae025_supplement.zip › Supplement Figures Peucedanum praeruptorum Dunn T2T-0217.docx]
