# Supplementary material for: The telomere-to-telomere (T2T) genome of Peucedanum praeruptorum Dunn provides insights into the genome evolution and coumarin biosynthesis
Source: Gigascience. 2024 Jun 5;13:giae025. doi: 10.1093/gigascience/giae025 (PMC11152176; doi:10.1093/gigascience/giae025)
Supplement: giae025_GIGA_D_23_00282_Original_Submission [file giae025_giga_d_23_00282_original_submission.pdf]

GigaScience

The telomere-to-telomere (T2T) genome of Peucedanum praeruptorum Dunn provides insights into the genome evolution and coumarin biosynthesis

--Manuscript Draft--

Manuscript Number:	GIGA-D-23-00282
Full Title:	The telomere-to-telomere (T2T) genome of Peucedanum praeruptorum Dunn provides insights into the genome evolution and coumarin biosynthesis
Article Type:	Research
Funding Information:	Key Technologies Research and Development Program (2022YFD1201600) — Dr Shancen Zhao
Abstract:	Background<br><br>Peucedanum praeruptorum Dunn. (Apiaceae) has been used in traditional Chinese medicine for a long time. Various coumarins including the major root constituents Praeruptorin (A-D) are the active constituents of the dried roots of P. praeruptorum. Previous transcriptomic and metabolomic studies attempted to elucidate the distribution and biosynthetic network for these medicinal valuable compounds. However, the lack of a high-quality reference genome impedes in-depth understanding of genetic traits and thus development of better breeding strategies.<br><br>Results<br><br>A telomere-to-telomere genome was assembled by the combination of PacBio HiFi, ONT ultra-long, and Hi-C data. The final genome assembly was approximately 1.798 Gb, assigned to 11 chromosomes, and genome completeness > 98%. Comparative genomic analysis suggested that P. praeruptorum experienced two WGD events as the ones in the Apiaceae family. By the transcriptomic and metabolomic analysis of coumarin metabolic pathway, we presented the spatial and temporal distribution of coumarins as well as the expression patterns of key genes for its biosynthesis. Notably, the COSY genes and cytochrome P450 genes showed tandem duplications on several chromosomes which may be responsible for the high accumulations of coumarins.<br><br>Conclusions<br><br>A T2T genome for P. praeruptorum was obtained which provides molecular insights into the chromosomal distribution of the coumarin biosynthetic genes. This high-quality genome serves as an important resource for designing engineering strategies for improving the production of these valuable compounds.
Corresponding Author:	Henrik Toft Simonsen<br>Jean Monnet University: Universite Jean Monnet Saint-Etienne<br>SAINT-ÉTIENNE, FRANCE
Corresponding Author Secondary Information:	
Corresponding Author's Institution:	Jean Monnet University: Universite Jean Monnet Saint-Etienne
Corresponding Author's Secondary Institution:	
First Author:	Mingzhou Bai
First Author Secondary Information:	
Order of Authors:	Mingzhou Bai
	Sanjie Jiang
	Shanshan Chu

Powered by Editorial Manager® and ProduXion Manager® from Aries Systems Corporation

	Yangyang Yu
	Dai Shan
	Chun Liu
	Weisong Xu
	Zhanlong Mei
	Liang Zong
	Qun Liu
	Jianbo Jian
	Chi Zhang
	Shancen Zhao
	Tsan-Yu Chiu
	Henrik Toft Simonsen
**Order of Authors Secondary Information:**	
**Additional Information:**	
**Question**	**Response**
Are you submitting this manuscript to a special series or article collection?	No
**Experimental design and statistics**<br><br>Full details of the experimental design and statistical methods used should be given in the Methods section, as detailed in our Minimum Standards Reporting Checklist. Information essential to interpreting the data presented should be made available in the figure legends.<br><br>Have you included all the information requested in your manuscript?	Yes
**Resources**<br><br>A description of all resources used, including antibodies, cell lines, animals and software tools, with enough information to allow them to be uniquely identified, should be included in the Methods section. Authors are strongly encouraged to cite Research Resource Identifiers (RRIDs) for antibodies, model organisms and tools, where possible.	Yes

Powered by Editorial Manager® and ProduXion Manager® from Aries Systems Corporation

Have you included the information requested as detailed in our Minimum Standards Reporting Checklist?	
**Availability of data and materials**<br><br>All datasets and code on which the conclusions of the paper rely must be either included in your submission or deposited in publicly available repositories (where available and ethically appropriate), referencing such data using a unique identifier in the references and in the “Availability of Data and Materials” section of your manuscript.<br><br>Have you have met the above requirement as detailed in our Minimum Standards Reporting Checklist?	Yes

Powered by Editorial Manager® and ProduXion Manager® from Aries Systems Corporation

Manuscript

Click here to access/download;Manuscript;Peucedanum praeruptorum Dunn.docx

The telomere-to-telomere (T2T) genome of *Peucedanum praeruptorum* Dunn provides insights into the genome evolution and coumarin biosynthesis

Mingzhou Bai[1,2,+], Sanjie Jiang[2,+], Shanshan Chu[3,4,+], Yangyang Yu[2], Dai Shan[2], Chun Liu[5], Weisong Xu[2], Zhanlong Mei[2], Liang Zong[6], Qun Liu[6], Jianbo Jian[1,2], Chi Zhang[2], Shancen Zhao[2], Tsan-Yu Chiu[2,*], Henrik Toft Simonsen[7,*]

[1] DTU Bioengineering, Technical University of Denmark, Kongens Lyngby 2800, Denmark, mingbai@dtu.dk

[2] BGI-Genomics, BGI-Shenzhen, Shenzhen 518000, China.

[3] School of Pharmacy, Anhui University of Chinese Medicine, Hefei 230000, China.

[4] Anhui Province Key Laboratory of Research & Development of Chinese Medicine, Hefei 230000, China.

[5] College of Tropical Crops, Hainan University, Haikou 570228, China

[6] Wuhan BGI Technology Service Co., Ltd. BGI-Wuhan, Wuhan 430000, China

[7] Laboratoire Biotechnologies Végétales Plantes aromatiques et médicinales, Université Jean Monnet, St. Étienne 42023, France.

* Corresponding author: henrik.toft.simonsen@univ-st-etienne.fr

[+] Contributed equally to the paper.

Abstract (250 words)

Background

Peucedanum praeruptorum Dunn. (Apiaceae) has been used in traditional Chinese medicine for a long time. Various coumarins including the major root constituents Praeruptorin (A-D) are the active constituents of the dried roots of *P. praeruptorum*. Previous transcriptomic and metabolomic studies attempted to elucidate the distribution and biosynthetic network for these medicinal valuable compounds. However, the lack of a high-quality reference genome impedes in-depth understanding of genetic traits and thus development of better breeding strategies.

Results

A telomere-to-telomere genome was assembled by the combination of PacBio HiFi, ONT ultra-long, and Hi-C data. The final genome assembly was approximately 1.798 Gb, assigned to 11 chromosomes, and genome completeness > 98%. Comparative genomic analysis suggested that *P. praeruptorum* experienced two WGD events as the ones in the Apiaceae family. By the

transcriptomic and metabolomic analysis of coumarin metabolic pathway, we presented the spatial and temporal distribution of coumarins as well as the expression patterns of key genes for its biosynthesis. Notably, the COSY genes and cytochrome P450 genes showed tandem duplications on several chromosomes which may be responsible for the high accumulations of coumarins.

Conclusions

A T2T genome for *P. praeruptorum* was obtained which provides molecular insights into the chromosomal distribution of the coumarin biosynthetic genes. This high-quality genome serves as an important resource for designing engineering strategies for improving the production of these valuable compounds.

Keywords

Peucedanum praeruptorum, T2T genome, coumarin biosynthesis

Background

Peucedanum praeruptorum Dunn. (Apiaceae) is a traditional Chinese medicinal plant with abundant contents of furano- and pyranocoumarins [1]. The dried root of *P. praeruptorum* has for centuries been used for the treatment of coughs with thick sputum and dyspnea, the active constituents were shown to be numerous coumarins including Praeruptorin A and Praeruptorin B) [2]. Thus, the roots' potential medicinal effects were related to the amount of furano- and pyranocoumarins. Pyranocoumarins isolated from the roots of *P. praeruptorum* have been found with multidrug resistance reversal activities and anti-inflammatory activities [3], treating headaches, coughing, and vomiting [4].

The genetic background of *P. praeruptorum* has not been well described. A recent study applied comparative transcriptomics to identify the crucial genes involved in coumarin biosynthesis in *P. praeruptorum*. This study found that the content of coumarins declines rapidly when *P. praeruptorum* reaches the bolting stage, and non-bolting has always been a decisive factor for harvesting the *P. praeruptorum* materials [1]. Several previous publications have shown that coevolution between plant and their predators is a matter of arms race. The biosynthesis of specialized metabolites in plants produces a vast array of defensive allelochemicals to cope with herbivores and pathogens [1]. On their side, many insect herbivores have evolved the ability to metabolize these defence compounds, thus have adapted to overcome much of the toxicity [3,4].

Furanocoumarins are well described in the plant kingdom [5]. Two kinds of furanocoumarins are described, which differ by the position of a furan group grafted on a coumarin core molecule either at position C6-C7 for linear molecules or C7-C8 for the angular one. Due to their chemical structure, the linear molecules are highly toxic. Although angular molecules are less toxic, the resistance spectrum is expanded when the plants simultaneously produce both linear and angular structures. These metabolites have been reported in presence predominantly in four plant families: Rutaceae, Moraceae, Fabaceae, and Apiaceae [6]. Apiaceae comprises all three possible evolutionary stages related to the occurrence of furanocoumarins in higher plants. Several species of Apiaceae are unable to synthesize these compounds, while others can synthesize only linear furanocoumarins. The remaining species, which belong to the subfamily Apioideae, contain both linear and angular molecules [7,8]. The subfamily Apioideae is the largest of the four subfamilies in Apiaceae, consisting of over 2900 species and more than 400 genera [9–12]. This diversity of species provides an excellent model to study the evolution of furanocoumarin formation in plants. By comparing the distribution and synthesis of these

components across different species of Apioideae, researchers can gain insight into how and why these compounds evolved [13].

The furanocoumarin biosynthesis is not yet fully resolved, while significant advances have been made in understanding the biosynthesis of coumarins and some of its derivatives, the full elucidation of furanocoumarin biosynthesis remains a complex and ongoing research interest. The same is true for pyranocoumarins with even less knowledge of their biosynthesis [14]. The intricate structures and unknown pathways of furano- and pyranocoumarins biosynthesis necessitate further research to fully unravel their biosynthesis mechanisms and potentially harness their applications.

The reduction of sequencing costs opens up great opportunities for genomics. The Omics datasets would benefit the elucidation of the genetic basis of its important traits such as increasing yields of certain bioactive compounds by integrating genome editing, genome wide selection and marker-assisted breeding technologies. Here, we report a telomere-to-telomere (T2T) genome of *P. praeruptorum* along with identifications of genes involved in the biosynthesis of the medically important coumarins. The genetic basis of these key traits in *P. praeruptorum* would provide a clear roadmap for future breeding and synthetic biology applications.

Methods

Plant materials and DNA/RNA isolation

The individual plants of *Peucedanum praeruptorum Dunn.* (Apiaceae) were collected between April 2022 and November 2022 in collaboration with and on the premises of the Anhui University of Chinese Medicine Garden in Heifei city, Anhui Province, China. The plant growth site was situated amidst the Huai River and the Yangtze River, commonly referred to as the Jianghuai area. Fresh, young, and healthy leaves were harvested for the extraction of high-molecular-weight genomic DNA using a modified cetyltri-methylammonium bromide (CTAB) method and nuclei method respectively for short reads and long reads (PacBio and Nanopore ultra-long) sequencing respectively. Samples from leaves, stems, roots, flowers, and fruit tissues at three different growth stages were utilized for RNA extraction employing a RNeasy PowerWater Kit (Qiagen, Carlsbad, CA, USA).

Library preparation and sequencing

The quality control and quantity assessment of the isolated DNA was conducted using a NanoDrop 2000 (Thermo Scientific, CA, USA) and a Qubit 2.0 Fluorometer (Life Technologies, CA, USA), respectively. Following purification with the Qiagen genomic kit (Qiagen, 13343), approximately 5 μg of *P. praeruptorum* DNA was utilized for constructing short DNA insert size (~350 bp) libraries using the MGIEasy Universal DNA Library Prep Kit and generating 20 kb PacBio HiFi sequencing libraries with the SMRTbell Prep Kit 2.0. Subsequently, short libraries were sequenced on an MGI-T7 sequencing platform with 150 bp paired-end reads. The SageHLS HMW library system (Sage Science, USA) was utilized to select approximately 10 μg of gDNA with a size of about 100 kb for the construction of an ultra-long Nanopore library using the ONT 1D Sequencing Kit (SQK-LSK109). PacBio HiFi sequencing and ultra-long ONT libraries were performed on the PacBio SequeII platform and Nanopore PromethION sequence, respectively. SMRT cell subread was generated and processed using the CCS algorithm of SMRTLink (v8.0.0) [15,16]. The MGIEasy RNA Directional Library Prep Kit (MGI) was utilized to construct RNA libraries, with approximately 1-2 μg of total RNA from each tissue sample employed. Subsequently, all libraries were subjected to sequencing on an MGISEQ-2000 platform, generating 150 bp paired-end reads.

The Hi-C library was prepared to facilitate the anchoring of assembled contigs to chromosomes through the following steps. The fresh young leaves were cross-linked using formaldehyde (Sigma), followed by resuspension in lysis buffer. Chromatins were fragmented using MboI (NEB) restriction endonucleases. Biotin labelling was performed, and crosslinking was achieved using T4 DNA Ligase (ENZYMATICS). The captured fragments were isolated using Streptavidin-coated magnetic beads (ThermoFisher SCIENTIFIC). An "A" base was added at the 3'-end of each strand using the KAPA HYPER PREP KIT (KAPA). After purification, the Hi-C library was sequenced with PE150 in the MGI-T7 sequencing platform.

Genome survey and De novo assembly

Before the long reads (PacBio and ONT) sequencing, the pilot genome survey was performed to decide the cost-effective strategies. With the 150-bp short reads, Jellyfish [17] and Genomescope 1.0 was used to predict the genomic characteristics [18]. The genome size and heterozygosity rate of the *P. praeruptorum* were determined through Kmer analysis.

With the HiFi long sequence reads, the initial *P. praeruptorum* contig assembly was performed by v0.7 with default parameters [19]. Then, the Hi-C reads were mapped the newly assembled initial contig sequences by Juicer v1.6 [16]. The contigs were anchored to the chromosome-level using 3D-DNA [20]. The interaction ma generated by Juicer was manual checking and the visual refinement by Juicebox Assembly Tools [16]. Following correction, the interaction map of the entire genome would exhibit robust inter-chromosome interactions, with stronger interactions observed between closer linear distances. The ultra-long ONT reads were aligned to the chromosome-level genome sequences using minimap2 [21] and facilitated the gap filing by TGS-GapCloser (v 1.0.1) [21] with the parameter "--min_match 2000". The gap-free genome sequence was obtained and error correction was performed on short reads using pilon [22] with default parameters. The completeness of the new genome and gene set was assessed using Benchmarking Universal Single-Copy Orthologs (BUSCO version 5.1.2) with the embryophyta_odb10 database, which comprises 1,614 conserved core eukaryotic genes [23].

Genome annotation

The newly gap-free assembled genomes of *P. praeruptorum* were utilized for the annotation of repetitive elements and genes. The annotation of repetitive sequences is performed using a combination of the following methods: (1) de novo prediction based on features of repeated sequences utilizing TRF 4.09 software [24]; (2) homology-based prediction method employing RepeatMasker software based on repeat database (http://www.girinst.org/repbase) [25]; (3) Ab Initio method: Construction of a custom library for repetitive sequence features using RepeatModeler v2.0 [26] (http://www.repeatmasker.org/RepeatModeler/) and LTR_FINDER v1.07 [27] (http://tlife.fudan.edu.cn/ltr_finder/). Then, de novo predictions were performed through RepeatMasker 3.3.0 [28].

The prediction of gene set was conducted by integrating three methods: (1) Homologous prediction based on homologs from nine closely related species (*Angelica sinensis*, *Apium graveolens*, *Aralia elata Miq*, *Coriandrum sativum*, *Daucus carota*, *Eleutherococcus senticosus*, *Oenanthe sinensis*, *Panax ginseng*, *Panax notoginseng*) using as Exonerate 2.2.0 [29] and Liftoff 1.6.3 [30]; (2) De novo prediction based on ab initio approaches, including AUGUSTUS v3.2.3 [31] and SNAP [32]; and (3) Transcriptome-based prediction was performed using RNAseq data. A total of 198Gb of the 33 samples of newly sequenced RNAseq data were mapped to the newly assembled genome sequences respectively using hista2.2.1 [33]. Then, stringtie2.1.6 [34]was employed for transcript identification and utilized

for transcript-assisted annotation purposes. Finally, the gene set of *P. praeruptorum* was integrated with three types of evidence through implementation of the MAKER pipeline (v3.31.8) [35].

Gene family and phylogenomic analysis

The genome sequences of *P. praeruptorum* and ten representative plants (*Angelica sinensis, Apium graveolens, Arabidopsis thaliana, Coriandrum sativum, Daucus carota, Oryza sativa, Panax notoginseng, Populus trichocarpa, Theobroma cacao, Vitis vinifera*) were utilized for gene family clustering and phylogenetic analysis. The gene sets of the 11 species included in the analysis were processed. In cases where multiple transcripts of a gene (resulting from variable splicing) existed in annotation files, only the longest transcript was retained. Genes encoding proteins with fewer than 30 amino acids or genes containing internal stop codons were excluded. The protein sequence similarity among all species was determined using an all-vs-all blastp (evalue 1e-5) approach, followed by gene family clustering using OrthoMCL Software-v2.0.9 L [36]. A total of 489 single-copy orthologous genes were identified and multi-sequence alignment of coding sequences were aligned using MAFFT v7.487 software [37]. Then, the gap positions were eliminated using Gblocks v0.91b software [38], followed by the construction of a phylogenetic tree using RaxML (version8.2.12) [39] with parameter (-fa -N 100 -m GTRGAMMA). Finally, the tree was rooted using TreeBest (https://github.com/Ensembl/treebest). The divergence time of 11 target plant species were inferred using MCMCtree of PAML (version 4.9j) [40], incorporating two calibrated divergence time intervals: Oryza sativa - *Vitis vinifera* (163.5-142.1 Mya) and *Panax notoginseng* - *Daucus carota* (69.0-54.3 Mya). The gene family expansion and contraction of 11 species were identified using the CAFE v4.2 pipeline [41] and the gene families were subjected to KEGG and GO enrichment analysis to elucidate their functional roles. The collinearity of genome and whole genome duplication (WGD) events were analyzed by WGDI pipeline[42]. Firstly, the protein sequences of *P. praeruptorum* were compared to those of three other species (*Angelica sinensis, Coriandrum sativum* and *Daucus carota)* through all-vs-all BLASTP analysis with an e-value threshold set at 1e-5. Subsequently, gene location information and chromosome length data were extracted. The synonymous substitution rate (Ks) of syntenic blocks were used to plot the dot.

RNA-Seq Data Analysis

The experiment involved a total of 33 samples, including three different tissue types at different growth stages: Vegetative growth stage (VP) - root, stem, leaf; Flowering stage (AP) - root, stem, leaf, flower; Fruiting stage (FP) - root, stem, leaf, fruit. Each tissue type was represented by three biological replicates. The low-quality raw reads of each sample were processed firstly using SOAPnuke (version 1 .5.2) [43]. The clean reads were aligned to the newly assembled references genome and gene sequence using HISAT v2.1.0 [33] and Bowtie2 [44], respectively. The gene and transcript expression levels were quantified using RSEM v1.2.8 [45]. Differentially expressed genes (DEGs) were calculated by DESeq2 [46]. The co-expression network was constructed using the WGCNA (v1.71) package in R [47]. The phenotypic data (the value of specialized metabolites in each tissue) were utilized and imported into the WGCNA framework, enabling calculation of correlation-based associations between them. Subsequently, the adjacency matrix was transformed into a topological overlap matrix using WGCNA. The node datasets and edgedatasets were imported in to cytoscape (v3.10.0) for the final figure. The genes (e.g. PAL, C4H, C3H, COMT, 4CL, C2'H, COSY, HCT, C3'H, CCoAOMT, F6'H, U-6-P, U-8-P) involved in the coumarin biosynthesis in *P. praeruptorum* were identified by using the genes from *A. Sinensis*, *A. thaliana*、*Z. officinale*、*P. sativa* as query. The genes with identity >=80 and coverage>=70% were selected. The heatmap of their expression patterns were displayed by pheatmap (v1.0.8) in R (https://CRAN.R-project.org/package=pheatmap).

Metabolic analysis

A total of 66 tissues were collected for metabolic analysis in accordance with the transcriptome samples, each tissue was represented by 6 biological replicates. The metabolite profiling from each tissue was performed using a nontargeted metabolomics approach, following the established protocol by Tohge and Fernie (2010) [48]. High-resolution mass spectrometry (HRMS) was conducted using an ultraperformance liquid chromatography (UPLC) system followed by The Q Exactive™ Plus Hybrid Quadrupole-Orbitrap™ Mass Spectrometer (MS). The base peak chromatogram (BPC) was utilized to represent a continuous depiction of the highest ion intensities recorded at each time point. All quality control samples were superimposed in both positive and negative ion modes, demonstrating excellent stability and high-quality data obtained from the instrument detection process. The differential metabolites

between the two biological groups were screened using univariate and multivariate analyses with a VIP⩾1, Fold Change⩾1.2 or ⩽0.83, and q-value < 0.05.

Analysis of coumarin biosynthesis-related cytochrome P450 gene family

The coumarin biosynthesis related cytochrome P450 gene family with CYP71A and CYP82C as query to blast the *P. praeruptorum* genomes with the similarity of 70% as cutoff. A phylogenetic tree these members was constructed using the adjacency method of the MEGA software [49]. The chromosome location of these identified cytochrome P450 genes was labeled, and the gene structure was visualized using TBtools software [50].

MEME online software was used to predict the motifs of these CYP genes, and the number was set to 10 [51]. The promoter region of each CYP gene with a length of 2000 bp was obtained from the genome, and cis-acting regulatory elements were predicted according to the PlantCARE database [52]. The predicted cis-elements were divided into seven functional categories: common, light, hormones, stress, development, others and flavonoids biosynthesis. A statistical histogram of the number of cis-elements was generated.

Results

Genome assembly

The preliminary genomic information of *P. praeruptorum* (Figure 1A) estimated a genome size of 1.78 Gb and the heterozygosity rate was calculated to be 1.3 % (Supplementary Figure S1 and Data Table S1). Further, we applied the PacBio HiFi sequencing to yield 726 Mb highly accurate long-read sequencing datasets (Supplementary Data Table S2). After initial assembly, a 1.874 Gb size draft genome was generated with a GC content of approximately 35.5%. A polished genome assembled with HIC technologies yielded a 1.87 Gb genome with 714 contigs and N50 reaching 159.879 Mb (Supplementary Data Table S3). In order to the missing parts of the genome, 350 Mb ONT Ultra-Long DNA sequencing data further improved the quality of the genome to 365 contigs (Supplementary Data Table S3). At last, combined with PacBio HiFi sequencing, ONT Ultra-Long DNA sequencing, HI-C assembly, and short reads correction, a telomere-to-telomere level genome with zero gaps in each chromosome was generated and anchored to 11 chromosomes (Figure 1B and 1C; Supplementary Data Table S4). This gapless genome consists of 253 contigs, N50 of 161.107 Mb, and with GC content 35.5% (Supplementary Data Table S4 & S5). The final HiC- heat map of this T2T genome is

presented in Figure 1D. To evaluate the accuracy of assembly sequences, the short-read and long-read data were mapped to the newly assembled sequences using BWA [53] and minimap2 [21]. Respectively, the 99.73% mapping rate and 99.91% coverage rate with depth >4 showed the high consistency between assembly results and reads, and also plot the GC content and depth distribution for analyzing the sequencing uniformity (Supplementary Figure S2). Furthermore, the Benchmarking Universal Single-Copy Orthology (BUSCO) analysis showed that the assembled genomes exhibit a completeness of more than 98.2% identified in the "eukaryote_odb10" database using BUSCO version 5.1.2 (Supplementary Data Table S6) [54].

Genome annotation

A total of 1.07 Gb of repeat sequences were detected, accounting for 59.67% of the assembled genome (Supplementary Data Table S8). This repeat content was less than the value (79.3%) predicted by k-mer analysis (Supplementary Table S1). The most abundant transposable elements were long terminal repeats (LTR), which account for 49.02% of the genome (Supplementary Data Table S9). A total of 247,398 and 164,100 protein coding genes were *de novo* predicted using the GlimmerHMM [55] and AUGUSTUS [56], respectively (Supplementary Data Table S10). Nine well-assembled plant genomes in Apiales, including *Panax ginseng*, *Angelica sinensis*, *Oenanthe sinensis*, *Eleutherococcus senticosus*, *Daucus carota*, *Apium graveolens*, *Aralia elata Miq*, *Coriandrum sativum*, and *Panax notoginseng* were used for homologous prediction. The predicted genes were integrated into a non-redundant and more complete gene set with 53,756 protein coding genes by MAKER2 [57] (Supplementary Figure S6, S7 & S8). A final reliable set of 44,468 high-confidence genes was obtained using the in-house script (Supplementary Data Table S10). The gene function of the protein-coding genes was defined by the following databases: NR (94.18%), SwissProt (56.85%), TrEMBL (93.83%), KOG (65.75%), TF (5.96%), InterPro (77.48%), GO (57.43%), KEGG_ALL (85.47%), KEGG_KO (32.73%) and Pfam (69.61%) (Supplementary Figure S3, S4 & S5). A total of 95.4 % protein-coding genes were annotated (Supplementary Data Table S11). We also annotated the non-coding RNAs and acquired 181 miRNAs, 2359 tRNAs, 6879 rRNAs, and 8823 snRNAs (Supplementary Data Table S12).

Evolutionary analysis

A phylogenetic tree was constructed to estimate the divergence time of *P. praeruptorum* and ten other representative plant species (Figure 2A and Supplementary Figure S9). *P.*

praeruptorum belongs to the Order of Apiales, they diverged from the other orders approximately 113.6 million years ago (Mya). Within the Apiales, *P. praeruptorum* clustered with its relatives in the Apiaceae family, which diverged from the Araliaceae family member *Panax notoginseng* about 62.5 Mya (Figure 2A). In total, 725 gene family contractions and 913 gene family expansions were detected in *P. praeruptorum.* Functional enrichment analysis was performed for those expansion and contraction genes (Supplementary Figure S10-S13). To investigate the species-specific and common gene families, homologous genes, gene family clusters, the enrichment of single copy genes and multiple copy genes were analysed in these 11 plant species (Figure 2B). *P. praeruptorum* carries a total of 44,468 genes which were clustered into 19402 gene families containing 489 single copy gene families (Figure 2B & Supplementary Data Table S13). Among the gene families, 4016 common gene families were shared with other plant species and 741 gene families were specific to *P. praeruptorum* (Figure 2C). We further performed collinearity analysis between *P. praeruptorum, D. carota, C. sativum* and *A. sinensis* the results showed that a few major chromosomal rearrangements occurred between those species (Figure 2D & Supplementary Figure S14). The distribution of synonymous substitutions per synonymous site (*Ks*) for Apiaceae plants were compared. Two major peaks were observed in the species analysed. This is consistent with the previous hypothesis that the Apiaceae members experienced two WGD events (Figure 2E).

As the first species to be separated out in our phylogeny in the Apiaceae, *D. carota* habours 9 chromosomes (haploid state). In contrast, many other members (e.g. *A. graveolens*, *A. sinensis*, *P. praeruptorum* and *C. sativum*) all have 11 chromosomes (haploid state) (Supplementary Figure S15) [58]The complete dot-plot based deconvolution into 11 reconstructed Conserved Ancestral Regions (CARs) of the observed synteny and paralogy among *P. Praeruptoru* and its Apiaceae siblings, suggested that the 11 proposed protochromosomes as the origin of Apiaceae. Our analysis is also consistent with the previous report that the modern celery chromosomes are well represented by the Apiaceae proto-chromosomes (Supplementary Figure S15)[58]. In addition, comparing *D. carota* with the other Apiaceae members with 11 chromosomes indicated that chromosome 10 and chromosome 6 experienced fission and fusion that led to the reduction of chromosome numbers in *D. carota* (Supplementary Figure S15).

Biosynthesis of coumarins

The well-known bioactive compounds Praeruptorin A-E in *P. praeruptorum* are pyranocumarins that have a pyran ring attached to the coumarin nucleus. In order to unravel

the distribution and biosynthesis of coumarin-related compounds in *P. praeruptorum*, we investigated its gene expression and metabolites at two key developmental stages: the vegetative growth stage, and the reproductive growth stage (e.g. flowering and fruit stage). Three different types of tissues: stems, leaves, and roots were collected. Notably, during the reproductive stage, the flowers and fruits were also collected (Figure 3).

The biosynthesis of coumarin is initiated at the phenylpropanoids pathway whereas the L-phenylalanine is catalyzed by PAL to form cinnamic acid. The cinnamic acid is further converted to *p*-coumaric acid by C4H and transformed into *p*-coumaroyl CoA by a member of the 4CL family. The CoA-esters are subsequently hydroxylated at the position ortho to the aromatic ring aliphatic side chain through either C2'H or F6'H. The coumarin core structure (e.g. Umbelliferone or Scopoletin) in the roots is catalysed by Coumarin synthase (COSY) [59]. Plant aromatic prenyltransferases are a group of enzymes that attach a prenyl group to aromatic compounds. Among these, umbelliferone dimethylallyltransferase (UDT) plays an important role in determining the structural diversification of coumarin-derived compounds. The UDT performed specific prenylation at the either C6 or C8 position of umbelliferone (Figure 3A), which led to linear or angular furanocoumarins or pyranocoumarins, respectively [60–62]. For *P. praeruptorum* the biosynthetic genes (PAL, C4H ,4CL, C2'H and F6'H) in the initial steps of phenylpropanoid pathway showed diverse spatial and temporal expression patterns since these compounds were common precursors for downstream anthocyanins, lignin and flavonoids pathways [63] (Figure 3A). While, the genes (C2'H, COSY, U-8-P and U-6-P) involved in the formation of umbelliferone and its derivatives are mainly expressed in the roots and stems at the vegetative stage (Supplementary Data Table S14 & S15). However, during the reproductive stage (e.g. anthesis and fruit), these transcripts showed reduced expression, which is in accordance with the metabolomic data.

The metabolomic analysis showed that the major forms of coumarins could be detected in the roots during the growth stages (Figure 3B). This was in consistent with the previous reports that coumarins were secreted into the rhizosphere for its allelopathic properties or to be involved in iron acquisition [64]. A few coumarins such as skimmin, rutarin, isopropylidenylacetyl-marmesin, isobergapten and decursinol were mainly observed in the leaves (Figure 3B), but in general the coumarins could be found in the roots and stems (Supplementary Data Table S16).

Coumarin Synthase (COSY)

Previously biochemical and molecular experiments done in the *Ruta graveolens* and Arabidopsis suggested that the trans–cis isomerization and lactonization forming the coumarin core structure was a spontaneous reaction and was catalyzed by lights [65–68]. However, a BAHD family member named COUMARIN SYNTHASE (COSY) was cloned in Arabidopsis and was demonstrated to catalyze the reaction without lights. The COSY is mainly expressed in the roots, an organ away from lights and is a conserved gene across many plant species [59] We used four *A. sinesis* COSY (AS10G01653; AS02G01453; AS10G00118 and AS11G01965) as the query to identify COSY genes in two other Apiaceae plants (e.g. *P. praeruptorum* and *D. carota*). Phylogenetic analysis of plant COSY enzymes showed that there are five major clades. Interestingly to note the multicollinearity comparison between *P. praeruptorum* and *A. Sinensis*, there was one more copy of COSY in *P. praeruptorum* on chromosome 10 which occurred by tandem duplication after they diverged from *A. Sinensis* (Figure 3C and Figure 2A). The expression patterns of this pair were slightly different at developmental stages. The expression of Ppra_10G0001290 was down regulated at the anthesis and the fruiting periods (Figure 3A). This suggests that these two genes may play different roles in coping with developmental needs. However, the detailed functions still need to be clarified.

Cytochrome P450 genes in coumarin biosynthesis

Plant Cytochrome P450s catalyze several regio- and stereo- specific hydroxylation that plays important roles in the general and specialized metabolites biosynthesis [69]. Based on the radioactive labeling of *Ammi majus* cell cultures, it suggested that the cytochrome P450s are involved in the coumarins biosynthesis[60]. Several members of the AYP71AJ subfamily have been cloned and characterized[13,70]. The AmCYP71AJ1 cloned from *A. majus* (Apiaceae) is responsible for catalyzing the linear furanocoumarins formation[71]. In contrast, the PsCYP71AJ4 from *Pastinaca sativa* is an angelicin synthase which is an angular furanocoumarin [70]. Further, we analyzed a total 48 cytochrome P450 genes by homology comparison with CYP71AJ gene family members of *A. sinesis* (Figure. 4). The phylogenetic tree demonstrated that these genes were distributed into three major lineages (e.g. PpCYP71AJ, PpCYP71AZ and PpCYP82C). Interesting to note that the members of PpCYP71AZ and PpCYP82C are expanded in *P. praeruptorum* compared with other Apiaceae members (e.g. *A. sinensis*, *C. sativum*, and *P. notoginseng*) (Figure 4D & Supplementary Data Table S18). These

expanded genes may be responsible for catalyzing the successive regio- and stereo- specific hydroxylation in the complex coumarins biosynthesis. This result is also consistent with the diverse coumarins detected in the *P. praeruptorum* (Figure 3; Supplementary Data Table S16). Based on the gene annotations, these CYP genes all shared similar gene structures which contain two main CDS and three major motifs in their cis-elements (Figure 4A and 4B). In addition to the common cis-elements, the light, hormones, and stress responsive elements were the three major types, which also suggested that the whole gene duplications are the major drive to expand this gene family. Several tandem and proximal duplications were observed except on chromosome 5, 8 and 10. Interestingly, no PpCYP71 nor PpCYP82 members can be identified on chromosome 5 (Figure 4E).

The systematic regulation of coumarin biosynthesis

Since the cis-elements of PpCYP71AJ members showed diverse regulatory elements, we developed a systematic view of the regulatory network of coumarin biosynthesis and the potential transcription factors (TFs) that are associated with these genes. A co-expression network connecting key node genes in coumarin biosynthesis with TFs were analyzed. The expression patterns of C3H (Ppra_11G0014640), 4CL (Ppra_2G0018300; Ppra_2G0018300), F6'H (Ppra_4G0007450), and COSY (Ppra_3G0025980) were found to be highly related to the expression patterns of numerous TFs (Figure 5). The MYB, bHLH, AP2-EREBP and WRKY were the four major TFs regulating coumarin biosynthesis. The R2R3-MYB and bHLH were well known that they together with WD40 form ternary complexes that positively or negatively regulate flavonoid biosynthesis genes [72,73]. The simple coumarin scopolin was known to be accumulated under abiotic stress conditions [74]. Based on our results, the AP2-EREBP and WRKY were biotic and/or abiotic stress response related TFs which further reflected the biosynthesis of these diverse coumarins to cope with various stresses conditions.

Discussions

P. praeruptorum is a valuable Chinese medicinal plant that is commonly used to treat coughing and as anti-mucus agent. Among the bioactive compounds, coumarins show high bioactivity to reduce multi-drug resistance in cancer cells with low toxicity [1]. Thus, *P. praeruptorum* is considered as a great resource for isolating these compounds. High quality reference genome could help uncover important traits and elucidate key catalytic enzymes for synthetic biology. A chromosome-level genome assembly of *Artemisia annua* revealed the artemisinin content is

correlated to the copy number of amorpha-4,11-diene synthase genes, as one representative example on how genomic information can help to improve plant specific metabolism[75]. Here, we present the first T2T genome of *P. praeruptorum*. Comparing the distribution of synonymous substitutions per synonymous site (Ks) in *P. praeruptorum* and the other Apiaceae species shows that the Apiaceae members experienced two WGD events. This is consistent with the previously published data and it may be a distinctive genomic signature of Apiaceae family [76–78]. In order to reconstruct plant chromosome evolution, the chromosomal collinear analysis was done comparing *P. praeruptorum*, *D. carota* and *A. sinensis*. Several chromosomal rearrangements have occurred to reshape the genome landscape of *P. praeruptorum* and are disclosed here (Figure 2D & Supplementary Figure S14).

The molecular basis of coumarin biosynthesis and their distribution have been described previously [64]. Most coumarins we evaluated here were also detected in the roots, which is consistent with the expression patterns of its biosynthetic genes. However, we also detected some of the coumarins are solely accumulated in the aerial parts which had not been disclosed in the past (Figure 3B). With this T2T genome, we characterized the cis-elements that are responsible for temporal and spatial regulation of these coumarin biosynthetic genes and potentially their transcription factors in the future. Likewise, the full-length genome allows us to focus on specific plant parts to discover the remaining genes in the Praeruptorins biosynthesis, it will also enable us to investigate whether the genes in the furano- and pyranocoumarin biosynthesis are clustered, like the COSY and CYP71AJ genes.

Data Availability

The genome sequencing data, including PacBio HiFi, ONT Ultra-long, DNBseq short reads, Hi-C data, and Transcriptome data have been deposited into the NCBI (https://www.ncbi.nlm.nih.gov/) database, the BioProject accession number PRJNA1011536.

Additional Files

Supplementary Figure S1: The K-mer depth distribution for *Peucedanum praeruptorum* Dunn genome size evaluation.

Supplementary Figure S2: Statistical graph of correlation analysis between GC content and Depth (short reads and long reads).

Supplementary Figure S3: GO enrichment annotation of *Peucedanum praeruptorum* Dunn.

Supplementary Figure S4: Kyoto Encyclopedia of Genes and Genomes (KEGG) enrichment annotation of *Peucedanum praeruptorum* Dunn.

Supplementary Figure S5: Venn diagram of functional annotation of *Peucedanum praeruptorum* Dunn in different databases.

Supplementary Figure S6: The Gene structure prediction results and gene set statistics (comparison with gene elements of closely related species).

Supplementary Figure S7: The cumulative distribution statistics graph of gene set element length (comparison with gene elements of closely related species).

Supplementary Figure S8: Venn diagram of homologous gene families of *Peucedanum praeruptorum* Dunn in the genome.

Supplementary Figure S9: The phylogenetic tree of the 11 species genomes with 489 single copy genes.

Supplementary Figure S10: GO enrichment results of *Peucedanum praeruptorum* Dunn contraction gene family.

Supplementary Figure S11： KEGG enrichment results of *Peucedanum praeruptorum* Dunn contraction gene family.

Supplementary Figure S12: GO enrichment results of *Peucedanum praeruptorum* Dunn expansion gene family.

Supplementary Figure S13: KEGG enrichment results of *Peucedanum praeruptorum* Dunn expansion gene family.

Supplementary Figure S14: The gene syntheny compaction among the *Angelica sinensis, Peucedanum praeruptorum* Dunn and *Daucus carota*.

Supplementary Figure S15: Inference of polyploidization and speciation history in Apiaceae.

Supplementary Figure S16: The phylogenetic tree of different species genomes with PT genes.

Supplementary Table S1: Statistical results of DNBSEQ platform sequencing data for genome survey.

Supplementary Table S2: Summary statistics for Pacbio HiFi data, ONT Ultra-long, and Hi-C data.

Supplementary Table S3: Statistics of genome assembly result of *Peucedanum praeruptorum* Dunn species.

Supplementary Table S4: Statistics of the T2T assembled chromosomes of *Peucedanum praeruptorum* Dunn.

Supplementary Table S5: Telomere sequence identification results

Supplementary Table S6: Completeness estimation of *Peucedanum praeruptorum* Dunn genome with Benchmarking Universal Single-Copy Orthologs (BUSCO) analysis.

Supplementary Table S7: Statistics of genes annotated in the *Peucedanum praeruptorum* Dunn genome.

Supplementary Table S8: The statistical results of repeat sequence.

Supplementary Table S9: Repeat sequence classification result statistics.

Supplementary Table S10: Basic statistical results of gene annotation prediction.

Supplementary Table S11: The statistical results of gene function annotation.

Supplementary Table S12: Statistics of non-coding RNA annotation results.

Supplementary Table S13: Summary of gene family clustering.

Supplementary Table S14: The genes associated with the coumarin metabolic pathway and the expression levels of those genes in various tissues and different growth stages.

Supplementary Table S15: The statistical results of PT gene in the four species were utilized for analysis.

Supplementary Table S16: The coumarin compounds data obtained through HPLC-MS analysis in different tissues.

Supplementary Table S17: The statistical results of the COSY genes in three species were utilized for analysis.

Supplementary Table S18: Phylogenomic analysis of cytochrome P450 multigene family.

List of abbreviations

BLAST: Basic Local Alignment Search Tool; Mb: megabase; Gb: gigabase; GO: Gene Ontology; KEGG: Kyoto Encyclopedia of Genes and Genomes; BUSCO: Benchmarking Universal Single-Copy Orthologs; DUP: duplication; CDS: coding sequence; LINE: long interspersed nucleotide element; Chr: Chromosome; TE: transposable element; LTR: long terminal repeat; HPLC: high-performance liquid chromatography; TD: tandem duplication; WGD: whole genome duplication.

Ethics approval and consent to participate

No ethical approval/permission is required to obtain the materials and perform the research in this study.

Competing interests

The authors declare that they have no competing interests.

Funding

This work was supported by the National Key Research and Development Program of China (Grant No.2022YFD1201600).

Authors' contributions

MB., SJ., and SC.: designed the research. MB., TYC.: performed analyses in the manuscript and prepared the manuscript. SC., LZ., QL., and ZM.: sampled and conducted the experiments. MB., YY., DS., CL., and WX.: analyzed the data. SJ., CZ., JJ., and SZ.: revised the manuscript and editing. HTS.: initiated the writing. SZ., TYC., and HTS.: conceived and supervised the project. All authors read and contributed to the final manuscript.

Figure Legends

Figure 1. Overview of the *P. praeruptorum* and its T2T genome.

A. The morphological characteristics of *P. praeruptorum* in three developmental stages. VP: Vegetative Period; AP: Anthesis Period; FP: Fruit Period. **B.** The circos plot from the outer to the inner circle represents eleven T2T chromosomes (Chr01-Chr11). The distribution of genome features within 3Mb windows are presented: a, GC contents; b, gene density; c, repeats density; d, LTR density; e, LINE density; f, DNA-TE density. **C.** The identifications of telomeres of the eleven chromosomes. The orange circles represent telomeres on the assembled chromosomes. The high gene densities are displayed in red and the low gene densities are displayed in blue. **D**. Hi-C heatmap demonstrated the interactions between eleven chromosomes.

Figure 2. Comparative genomic analysis of the *P. praeruptorum* T2T genome.

A. The estimation of divergence time and gene family expansion/contraction. The numbers next to each branch node represent the estimated divergence time (million years ago, Mya),

with confidence range in brackets. The pie chart demonstrated the ratio of gene families with expansion (green), contraction (red) and stable (blue). **B.** Number of homologous genes shared by different species. **C.** The gene family clustering demonstrated by Petal Map. The middle circle is the number of gene families common to all species, and the edge is the number of gene families unique to each species. **D.** The chromosomal collinearity among *C. sativum, P. praeruptorum* and *D. carota*. **E.** The Ks distribution map within and between species

Figure 3. The coumarins biosynthesis pathway in *P. praeruptorum.*
A. The coumarin biosynthesis candidate genes identification in *P. praeruptorum*. The gene expressions were measures in 3 different tissues (root, stem and leaf) and 3 different developmental periods (Vegetative period, VP; Anthesis period, AP and Fruiting period, FP). **B.** The distribution of coumarins in 5 different tissues and 3 different developmental periods. **C.** The phylogenetic analysis of COSY and collinearity analysis between *A. sinensis* and *P. praeruptorum***.**

Figure 4. Cytochrome P450 genes related to coumarins biosynthesis in *P. praeruptorum*.
A. Motif analysis in cis-elements of the coumarins biosynthesis related cytochrome P450 genes. **B.** The gene structures of the coumarins biosynthesis related cytochrome P450 genes. **C.** The analysis of key cis-elements identified in the promoter regions of coumarin-related cytochrome P450 genes. **D.** Phylogenetic tree of cytochrome P450 genes involved in the coumarin biosynthesis. **E.** The gene location of coumarin-related cytochrome P450 genes on the chromosomes.

Figure 5. A co-expression network connecting structural genes in coumarin biosynthesis with transcription factors (TFs) represents the regulation of coumarin biosynthetic genes.
The nodes represent structural genes in coumarin biosynthesis and transcription factors. The expression changes of each gene (log2FoldChange (root_fp/root_vp)) is shown by the node size. The number of TF associated with the core biosynthetic genes are demonstrated by the numbers of the nodes.

Reference

1. Song C, Li X, Jia B, Liu L, Wei P, Manzoor MA, et al. Comparative Transcriptomics Unveil the Crucial Genes Involved in Coumarin Biosynthesis in *Peucedanum praeruptorum* Dunn. *Front Plant Sci*. Frontiers Media S.A.; 2022; doi: 10.3389/fpls.2022.899819.
2. Song Y, Jing W, Yan R, Wang Y. Research progress of the studies on the roots of *Peucedanum praeruptorum* dunn (Peucedani radix). *Pak J Pharm Sci*. 28:71–812015;
3. Lee J, Lee YJ, Kim J, Bang O-S. Pyranocoumarins from Root Extracts of *Peucedanum praeruptorum* Dunn with Multidrug Resistance Reversal and Anti-Inflammatory Activities. *Molecules*. 2015; doi: 10.3390/molecules201219738.
4. Sarkhail P. Traditional uses, phytochemistry and pharmacological properties of the genus *Peucedanum*: a review. *J Ethnopharmacol*. 2014; doi: 10.1016/j.jep.2014.08.034.
5. Río JA Del, Díaz L, García-Bernal D, Blanquer M, Ortuño A, Correal E, et al Chapter 5 - Furanocoumarins: Biomolecules of Therapeutic Interest. In: Atta-ur-Rahman, editor. Elsevier;
6. Bourgaud F, Hehn A, Larbat R, Doerper S, Gontier E, Kellner S, et al. Biosynthesis of coumarins in plants: A major pathway still to be unravelled for cytochrome P450 enzymes. Phytochemistry Reviews.
7. Seigler DS. Coumarins. *Plant Secondary Metabolism*. Boston, MA: Springer US;
8. Berenbaum MR. Chemical Mediation of Coevolution: Phylogenetic Evidence for. *Annals of the Missouri Botanical Garden*. 88:45–592001;
9. Downie SR, Spalik K, Katz-Downie DS, Reduron J-P. Major clades within Apiaceae subfamily Apioideae as inferred by phylogenetic analysis of nrDNA ITS sequences. *Plant Divers Evol*. Schweizerbart; 2010; doi: 10.1127/1869-6155/2010/0128-0005.
10. Magee AR, Calviño CI, Liu MR, Downie SR, Tilney PM, van Wyk BE. New tribal delimitations for the early diverging lineages of Apiaceae subfamily Apioideae. *Taxon*. International Association for Plant Taxonomy; 2010; doi: 10.1002/tax.592021.
11. Spalik K, Piwczyński M, Danderson CA, Kurzyna-Młynik R, Bone TS, Downie SR. Amphitropic amphiantarctic disjunctions in Apiaceae subfamily Apioideae. *J Biogeogr*. 2010; doi: 10.1111/j.1365-2699.2010.02334.x.
12. Weitzel C, Rønsted N, Spalik K, Simonsen HT. Resurrecting deadly carrots: towards a revision of Thapsia (Apiaceae) based on phylogenetic analysis of nrITS sequences and chemical profiles. *Botanical Journal of the Linnean Society*. 2014; doi: 10.1111/boj.12144.
13. Dueholm B, Krieger C, Drew D, Olry A, Kamo T, Taboureau O, et al. Evolution of substrate recognition sites (SRSs) in cytochromes P450 from Apiaceae exemplified by the CYP71AJ subfamily. *BMC Evol Biol*. 2015; doi: 10.1186/s12862-015-0396-z.

14. Khandy MT, Sofronova AK, Gorpenchenko TY, Chirikova NK. Plant Pyranocoumarins: Description, Biosynthesis, Application. *Plants (Basel)*. 2022; doi: 10.3390/plants11223135.

15. Chin C-S, Alexander DH, Marks P, Klammer AA, Drake J, Heiner C, et al.. Nonhybrid, finished microbial genome assemblies from long-read SMRT sequencing data. *Nat Methods*. 2013; doi: 10.1038/nmeth.2474.

16. Durand NC, Robinson JT, Shamim MS, Machol I, Mesirov JP, Lander ES, et al.. Juicebox Provides a Visualization System for Hi-C Contact Maps with Unlimited Zoom. *Cell Syst*. 2016; doi: 10.1016/j.cels.2015.07.012.

17. Marçais G, Kingsford C. A fast, lock-free approach for efficient parallel counting of occurrences of k-mers. *Bioinformatics*. 2011; doi: 10.1093/bioinformatics/btr011.

18. Vurture GW, Sedlazeck FJ, Nattestad M, Underwood CJ, Fang H, Gurtowski J, et al.. GenomeScope: fast reference-free genome profiling from short reads. *Bioinformatics*. 2017; doi: 10.1093/bioinformatics/btx153.

19. Cheng H, Concepcion GT, Feng X, Zhang H, Li H. Haplotype-resolved de novo assembly using phased assembly graphs with hifiasm. *Nat Methods*. 2021; doi: 10.1038/s41592-020-01056-5.

20. Dudchenko O, Batra SS, Omer AD, Nyquist SK, Hoeger M, Durand NC, et al.. De novo assembly of the Aedes aegypti genome using Hi-C yields chromosome-length scaffolds. *Science*. 2017; doi: 10.1126/science.aal3327.

21. Li H. Minimap2: pairwise alignment for nucleotide sequences. *Bioinformatics*. 2018; doi: 10.1093/bioinformatics/bty191.

22. Walker BJ, Abeel T, Shea T, Priest M, Abouelliel A, Sakthikumar S, et al.. Pilon: an integrated tool for comprehensive microbial variant detection and genome assembly improvement. *PLoS One*. 2014; doi: 10.1371/journal.pone.0112963.

23. Manni M, Berkeley MR, Seppey M, Simão FA, Zdobnov EM. BUSCO Update: Novel and Streamlined Workflows along with Broader and Deeper Phylogenetic Coverage for Scoring of Eukaryotic, Prokaryotic, and Viral Genomes. *Mol Biol Evol*. 2021; doi: 10.1093/molbev/msab199.

24. Benson G. Tandem repeats finder: a program to analyze DNA sequences. *Nucleic Acids Res*. 1999; doi: 10.1093/nar/27.2.573.

25. Bao W, Kojima KK, Kohany O. Repbase Update, a database of repetitive elements in eukaryotic genomes. *Mob DNA*. 2015; doi: 10.1186/s13100-015-0041-9.

26. Flynn JM, Hubley R, Goubert C, Rosen J, Clark AG, Feschotte C, et al.. RepeatModeler2 for automated genomic discovery of transposable element families. *Proceedings of the National Academy of Sciences*. 2020; doi: 10.1073/pnas.1921046117.

27. Xu Z, Wang H. LTR_FINDER: an efficient tool for the prediction of full-length LTR retrotransposons. *Nucleic Acids Res*. 2007; doi: 10.1093/nar/gkm286.

28. Saha S, Bridges S, Magbanua Z V., Peterson DG. Empirical comparison of ab initio repeat finding programs. *Nucleic Acids Res*. 2008; doi: 10.1093/nar/gkn064.

29. Slater G, Birney E. Automated generation of heuristics for biological sequence comparison. *BMC Bioinformatics*. 2005; doi: 10.1186/1471-2105-6-31.

30. Shumate A, Salzberg SL. Liftoff: accurate mapping of gene annotations. *Bioinformatics*. 2021; doi: 10.1093/bioinformatics/btaa1016.

31. Stanke M, Schöffmann O, Morgenstern B, Waack S. Gene prediction in eukaryotes with a generalized hidden Markov model that uses hints from external sources. *BMC Bioinformatics*. 2006; doi: 10.1186/1471-2105-7-62.

32. Korf I. Gene finding in novel genomes. *BMC Bioinformatics*. 2004; doi: 10.1186/1471-2105-5-59.

33. Kim D, Langmead B, Salzberg SL. HISAT: a fast spliced aligner with low memory requirements. *Nat Methods*. 2015; doi: 10.1038/nmeth.3317.

34. Kovaka S, Zimin A V., Pertea GM, Razaghi R, Salzberg SL, Pertea M. Transcriptome assembly from long-read RNA-seq alignments with StringTie2. *Genome Biol*. 2019; doi: 10.1186/s13059-019-1910-1.

35. Holt C, Yandell M. MAKER2: an annotation pipeline and genome-database management tool for second-generation genome projects. *BMC Bioinformatics*. 2011; doi: 10.1186/1471-2105-12-491.

36. Li L, Stoeckert CJ, Roos DS. OrthoMCL: Identification of Ortholog Groups for Eukaryotic Genomes. *Genome Res*. 2003; doi: 10.1101/gr.1224503.

37. Katoh K, Standley DM. MAFFT Multiple Sequence Alignment Software Version 7: Improvements in Performance and Usability. *Mol Biol Evol*. 2013; doi: 10.1093/molbev/mst010.

38. Castresana J. Selection of Conserved Blocks from Multiple Alignments for Their Use in Phylogenetic Analysis. *Mol Biol Evol*. 2000; doi: 10.1093/oxfordjournals.molbev.a026334.

39. Stamatakis A. RAxML version 8: a tool for phylogenetic analysis and post-analysis of large phylogenies. *Bioinformatics*. 2014; doi: 10.1093/bioinformatics/btu033.

40. Yang Z. PAML 4: Phylogenetic Analysis by Maximum Likelihood. *Mol Biol Evol*. 2007; doi: 10.1093/molbev/msm088.

41. De Bie T, Cristianini N, Demuth JP, Hahn MW. CAFE: a computational tool for the study of gene family evolution. *Bioinformatics*. 2006; doi: 10.1093/bioinformatics/btl097.

42. Sun P, Jiao B, Yang Y, Shan L, Li T, Li X, et al.. WGDI: A user-friendly toolkit for evolutionary analyses of whole-genome duplications and ancestral karyotypes. *Mol Plant*. 2022; doi: 10.1016/j.molp.2022.10.018.

43. Chen Y, Chen Y, Shi C, Huang Z, Zhang Y, Li S, et al.. SOAPnuke: a MapReduce acceleration-supported software for integrated quality control and preprocessing of high-throughput sequencing data. *Gigascience*. 2018; doi: 10.1093/gigascience/gix120.

44. Langmead B, Salzberg SL. Fast gapped-read alignment with Bowtie 2. *Nat Methods*. 2012; doi: 10.1038/nmeth.1923.

45. Li B, Dewey CN. RSEM: accurate transcript quantification from RNA-Seq data with or without a reference genome. *BMC Bioinformatics*. 2011; doi: 10.1186/1471-2105-12-323.

46. Love MI, Huber W, Anders S. Moderated estimation of fold change and dispersion for RNA-seq data with DESeq2. *Genome Biol*. 2014; doi: 10.1186/s13059-014-0550-8.

47. Langfelder P, Horvath S. WGCNA: an R package for weighted correlation network analysis. *BMC Bioinformatics*. 2008; doi: 10.1186/1471-2105-9-559.

48. Tohge T, Fernie AR. Combining genetic diversity, informatics and metabolomics to facilitate annotation of plant gene function. *Nat Protoc*. 2010; doi: 10.1038/nprot.2010.82.

49. Tamura K, Stecher G, Peterson D, Filipski A, Kumar S. MEGA6: Molecular Evolutionary Genetics Analysis Version 6.0. *Mol Biol Evol*. 2013; doi: 10.1093/molbev/mst197.

50. Chen C, Chen H, Zhang Y, Thomas HR, Frank MH, He Y, et al.. TBtools: An Integrative Toolkit Developed for Interactive Analyses of Big Biological Data. *Mol Plant*. 2020; doi: 10.1016/j.molp.2020.06.009.

51. Bailey TL, Boden M, Buske FA, Frith M, Grant CE, Clementi L, et al.. MEME SUITE: tools for motif discovery and searching. *Nucleic Acids Res*. 2009; doi: 10.1093/nar/gkp335.

52. Lescot M. PlantCARE, a database of plant cis-acting regulatory elements and a portal to tools for in silico analysis of promoter sequences. *Nucleic Acids Res*. 2002; doi: 10.1093/nar/30.1.325.

53. Jung Y, Han D. BWA-MEME: BWA-MEM emulated with a machine learning approach. *Bioinformatics*. 2022; doi: 10.1093/bioinformatics/btac137.

54. Manni M, Berkeley MR, Seppey M, Simão FA, Zdobnov EM. BUSCO Update: Novel and Streamlined Workflows along with Broader and Deeper Phylogenetic Coverage for Scoring of

Eukaryotic, Prokaryotic, and Viral Genomes. *Mol Biol Evol*. 2021; doi: 10.1093/molbev/msab199.

55. Majoros WH, Pertea M, Salzberg SL. TigrScan and GlimmerHMM: two open source *ab initio* eukaryotic gene-finders. *Bioinformatics*. 2004; doi: 10.1093/bioinformatics/bth315.

56. Stanke M, Steinkamp R, Waack S, Morgenstern B. AUGUSTUS: a web server for gene finding in eukaryotes. *Nucleic Acids Res*. 2004; doi: 10.1093/nar/gkh379.

57. Holt C, Yandell M. MAKER2: an annotation pipeline and genome-database management tool for second-generation genome projects. *BMC Bioinformatics*. 2011; doi: 10.1186/1471-2105-12-491.

58. Song X, Sun P, Yuan J, Gong K, Li N, Meng F, et al.. The celery genome sequence reveals sequential paleo-polyploidizations, karyotype evolution and resistance gene reduction in apiales. *Plant Biotechnol J*. Blackwell Publishing Ltd; 2021; doi: 10.1111/pbi.13499.

59. Vanholme R, Sundin L, Seetso KC, Kim H, Liu X, Li J, et al.. COSY catalyses trans–cis isomerization and lactonization in the biosynthesis of coumarins. *Nat Plants*. Palgrave Macmillan Ltd.; 2019; doi: 10.1038/s41477-019-0510-0.

60. Hamerski D, Schmitt D, Matern U. Induction of two prenyltransferases for the accumulation of coumarin phytoalexins in elicitor-treated Ammi majus cell suspension cultures. *Phytochemistry*. 1990; doi: 10.1016/0031-9422(90)85417-E.

61. Dhillon DS, Brown SA. Localization, purification, and characterization of dimethylallylpyrophosphate:Umbelliferone dimethylallyltransferase from Ruta graveolens. *Arch Biochem Biophys*. 1976; doi: 10.1016/0003-9861(76)90417-3.

62. Ellis BE, Brown SA. Isolation of Dimethylallylpyrophosphate:Umbelliferone Dimethylallyltransferase from *Ruta graveolens*. *Can J Biochem*. 1974; doi: 10.1139/o74-104.

63. Vogt T. Phenylpropanoid Biosynthesis. *Mol Plant*. 2010; doi: 10.1093/mp/ssp106.

64. Robe K, Izquierdo E, Vignols F, Rouached H, Dubos C. The Coumarins: Secondary Metabolites Playing a Primary Role in Plant Nutrition and Health. *Trends Plant Sci*. 2021; doi: 10.1016/j.tplants.2020.10.008.

65. Karamat F, Olry A, Doerper S, Vialart G, Ullmann P, Werck-Reichhart D, et al.. CYP98A22, a phenolic ester 3’-hydroxylase specialized in the synthesis of chlorogenic acid, as a new tool for enhancing the furanocoumarin concentration in Ruta graveolens. *BMC Plant Biol*. 2012; doi: 10.1186/1471-2229-12-152.

66. Kai K, Mizutani M, Kawamura N, Yamamoto R, Tamai M, Yamaguchi H, et al.. Scopoletin is biosynthesized via *ortho* -hydroxylation of feruloyl CoA by a 2-oxoglutarate-dependent

dioxygenase in *Arabidopsis thaliana*. *The Plant Journal*. 2008; doi: 10.1111/j.1365-313X.2008.03568.x.

67. Vialart G, Hehn A, Olry A, Ito K, Krieger C, Larbat R, et al.. A 2-oxoglutarate-dependent dioxygenase from Ruta graveolens L. exhibits p-coumaroyl CoA 2′-hydroxylase activity (C2′H): a missing step in the synthesis of umbelliferone in plants. *The Plant Journal*. 2012; doi: 10.1111/j.1365-313X.2011.04879.x.

68. Matsumoto S, Mizutani M, Sakata K, Shimizu B-I. Molecular cloning and functional analysis of the ortho-hydroxylases of p-coumaroyl coenzyme A/feruloyl coenzyme A involved in formation of umbelliferone and scopoletin in sweet potato, Ipomoea batatas (L.) Lam. *Phytochemistry*. 2012; doi: 10.1016/j.phytochem.2011.11.009.

69. Nelson D, Werck-Reichhart D. A P450-centric view of plant evolution. *Plant Journal*. 2011; doi: 10.1111/j.1365-313X.2011.04529.x.

70. Larbat R, Hehn A, Hans J, Schneider S, Jugde H, Schneider B, et al.. Isolation and functional characterization of CYP71AJ4 encoding for the first P450 monooxygenase of angular furanocoumarin biosynthesis. *Journal of Biological Chemistry*. 2009; doi: 10.1074/jbc.M807351200.

71. Hehmann M, Lukačin R, Ekiert H, Matern U. Furanocoumarin biosynthesis in Ammi majus L. Cloning of bergaptol O-methyltransferase. *Eur J Biochem*. 2004; doi: 10.1111/j.1432-1033.2004.03995.x.

72. Xu W, Dubos C, Lepiniec L. Transcriptional control of flavonoid biosynthesis by MYB–bHLH–WDR complexes. *Trends Plant Sci*. 2015; doi: 10.1016/j.tplants.2014.12.001.

73. Sun B, Zhu Z, Cao P, Chen H, Chen C, Zhou X, et al.. Purple foliage coloration in tea (Camellia sinensis L.) arises from activation of the R2R3-MYB transcription factor CsAN1. *Sci Rep*. 2016; doi: 10.1038/srep32534.

74. Döll S, Kuhlmann M, Rutten T, Mette MF, Scharfenberg S, Petridis A, et al.. Accumulation of the coumarin scopolin under abiotic stress conditions is mediated by the *Arabidopsis thaliana* <scp>THO</scp> / <scp>TREX</scp> complex. *The Plant Journal*. 2018; doi: 10.1111/tpj.13797.

75. Liao B, Shen X, Xiang L, Guo S, Chen S, Meng Y, et al.. Allele-aware chromosome-level genome assembly of Artemisia annua reveals the correlation between ADS expansion and artemisinin yield. *Mol Plant*. 2022; doi: 10.1016/j.molp.2022.05.013.

76. Liu JX, Liu H, Tao JP, Tan GF, Dai Y, Yang LL, et al.. High-quality genome sequence reveals a young polyploidization and provides insights into cellulose and lignin biosynthesis in

water dropwort (Oenanthe sinensis). *Ind Crops Prod*. Elsevier B.V.; 2023; doi: 10.1016/j.indcrop.2022.116203.

77. Han X, Li C, Sun S, Ji J, Nie B, Maker G, et al.. The chromosome-level genome of female ginseng (Angelica sinensis) provides insights into molecular mechanisms and evolution of coumarin biosynthesis. *Plant Journal*. John Wiley and Sons Inc; 2022; doi: 10.1111/tpj.16007.

78. Wang Y-H, Liu P-Z, Liu H, Zhang R-R, Liang Y, Xu Z-S, et al.. Telomere-to-telomere carrot (*Daucus carota*) genome assembly reveals carotenoid characteristics. *Hortic Res*. Oxford University Press (OUP); 2023; doi: 10.1093/hr/uhad103.

Figure 1

Click here to access/download;Figure;Figure 1 Overview of the P praeruptorum and its T2T genome.pdf

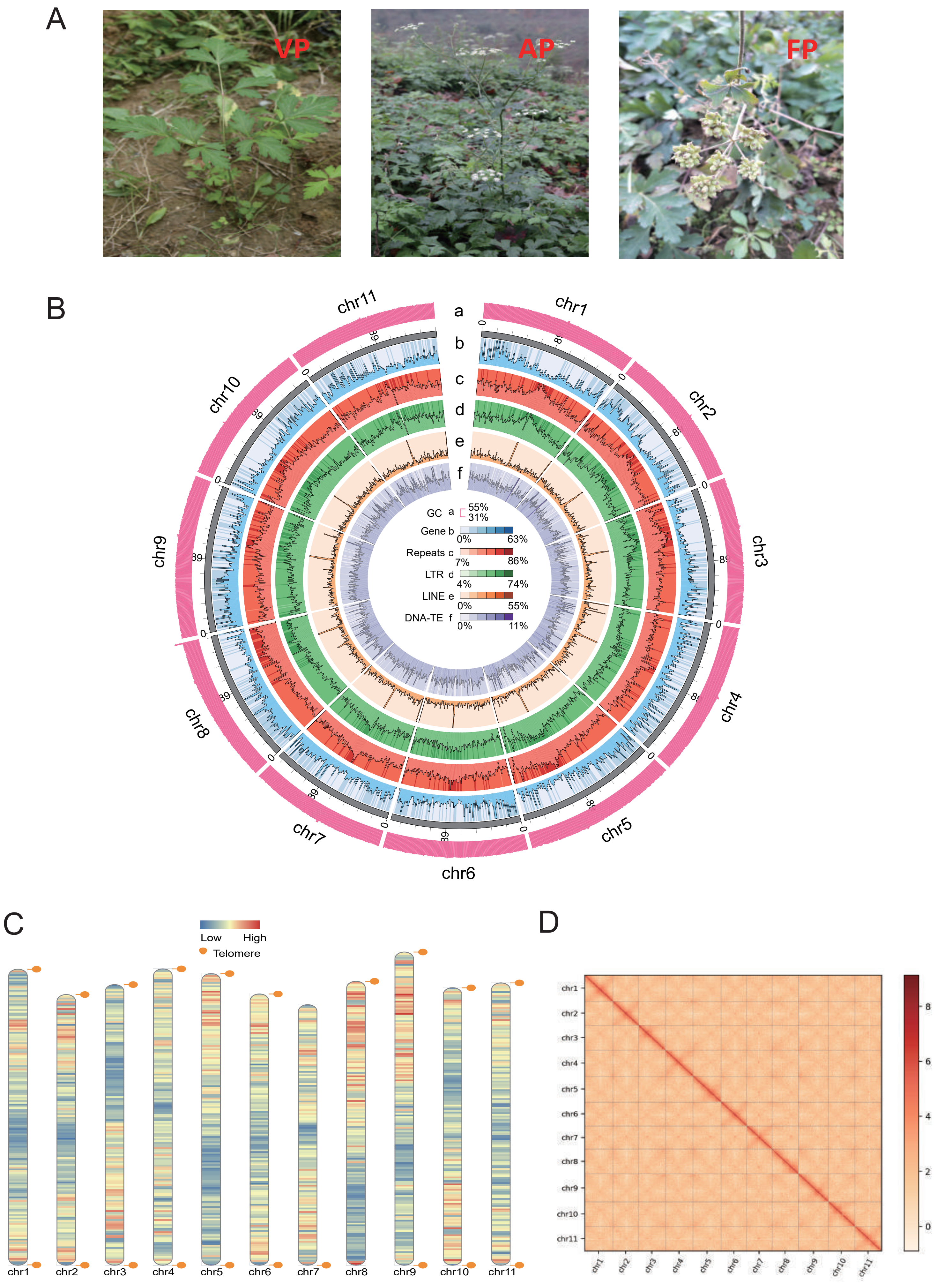

Figure 2

Click here to access/download;Figure;Figure 2 Comparative genomic analysis.pdf

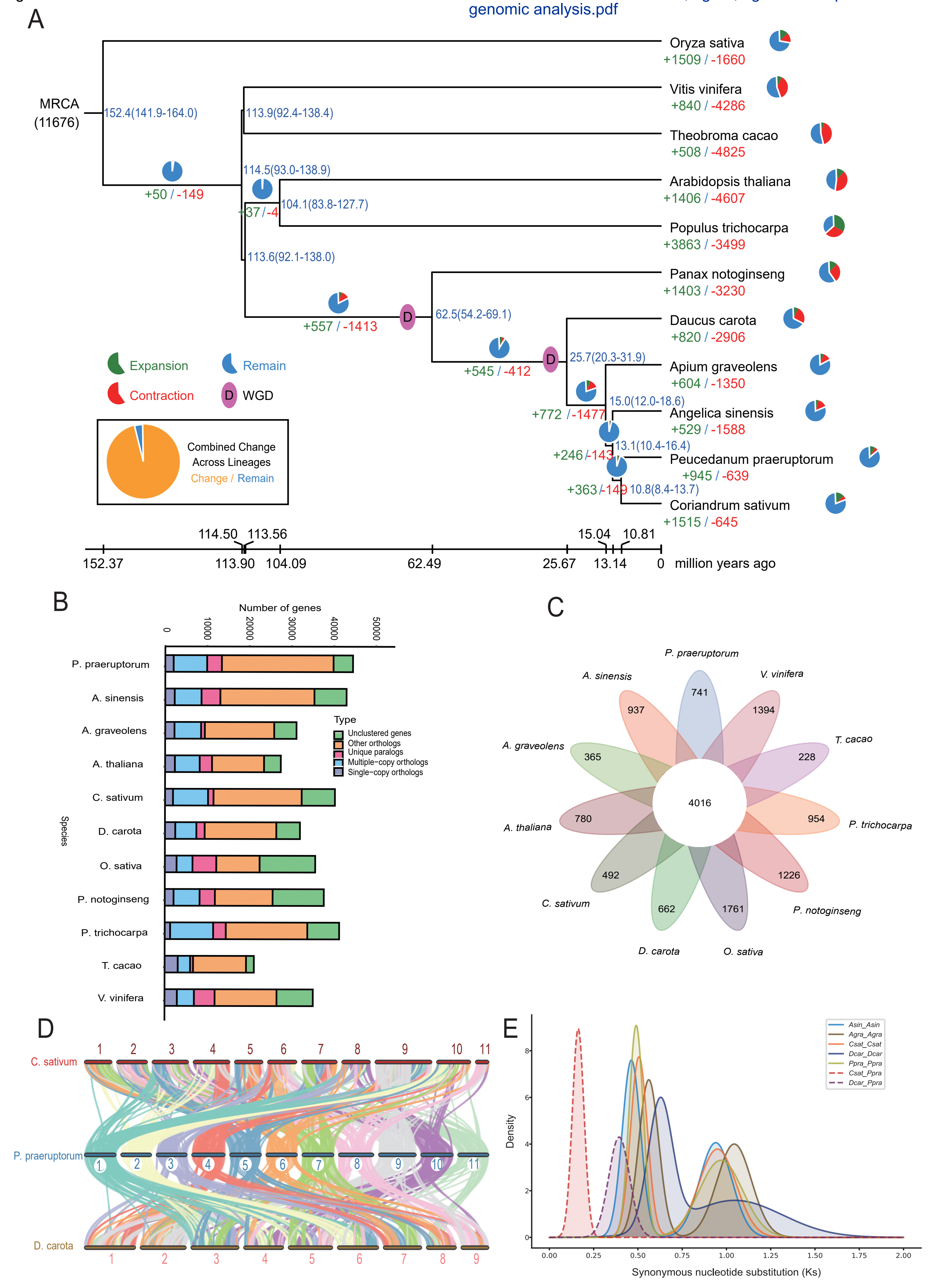

Figure 3

Click here to access/download;Figure;Figure 3 The coumarins biosynthesis pathway.pdf

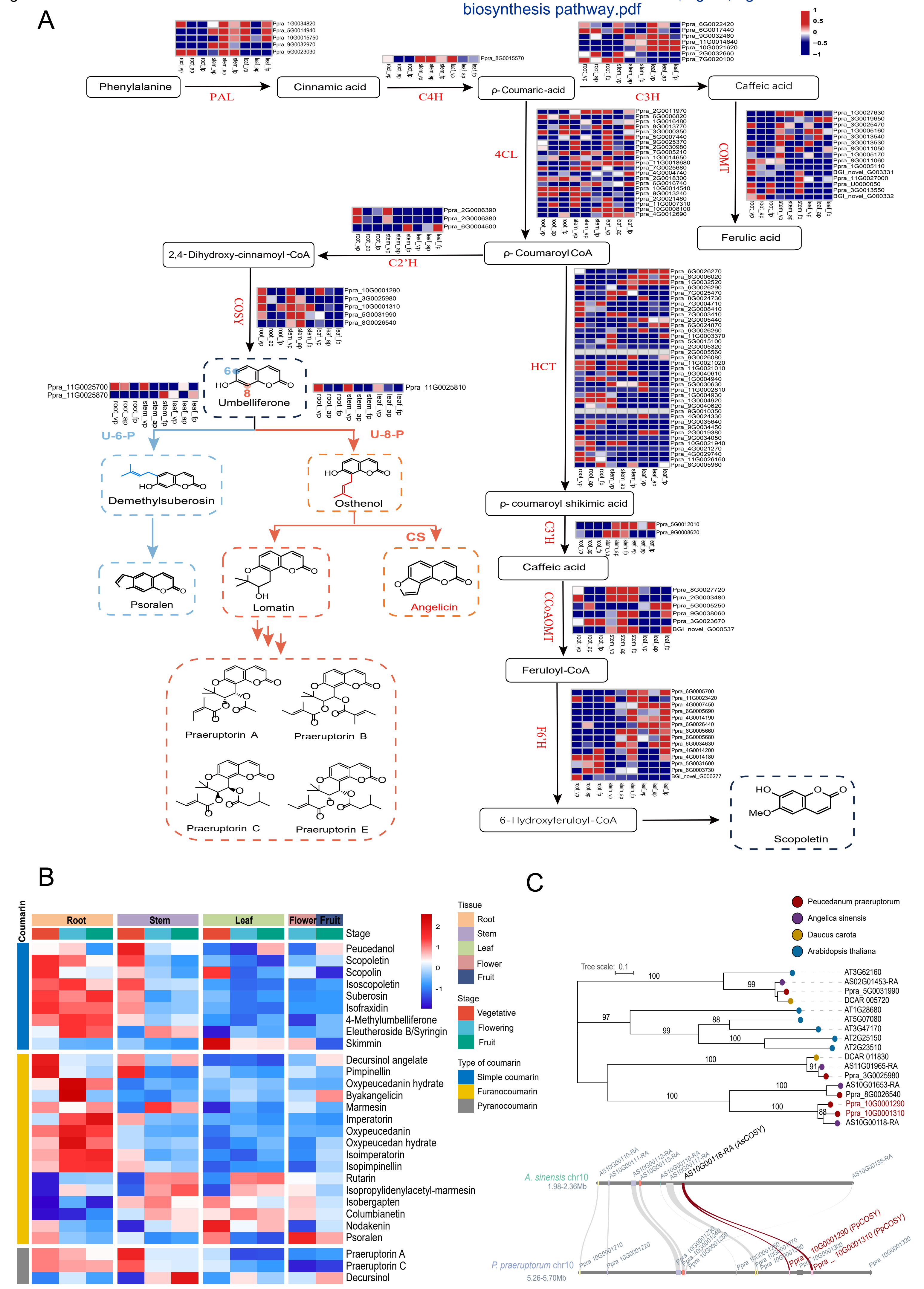

Click here to access/download;Figure;Figure 4 Cytochrome P450 genes related to coumarins.pdf

Figure 4

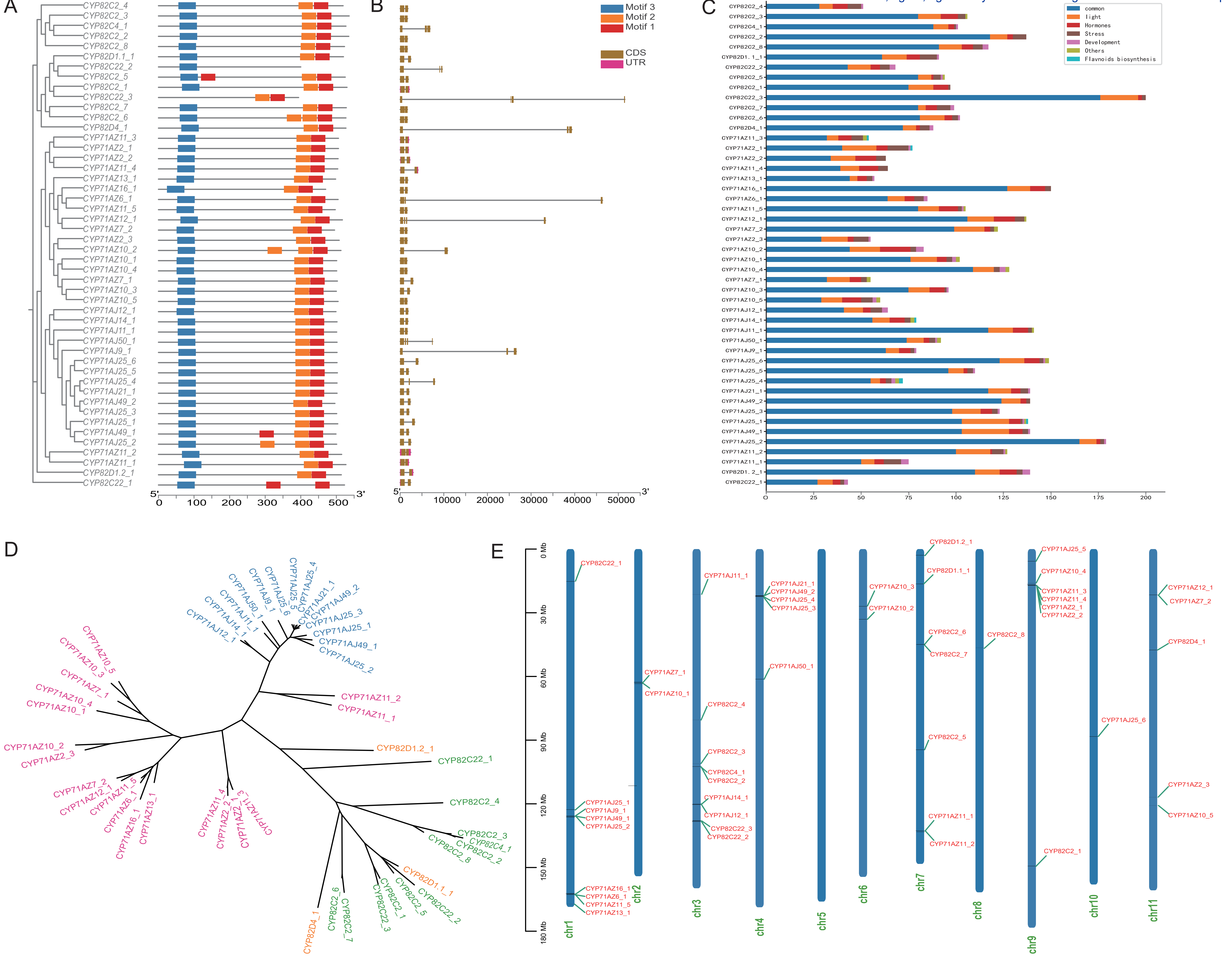

Figure 5

Click here to access/download;Figure;Figure 5 Co-expression Analysis.pdf

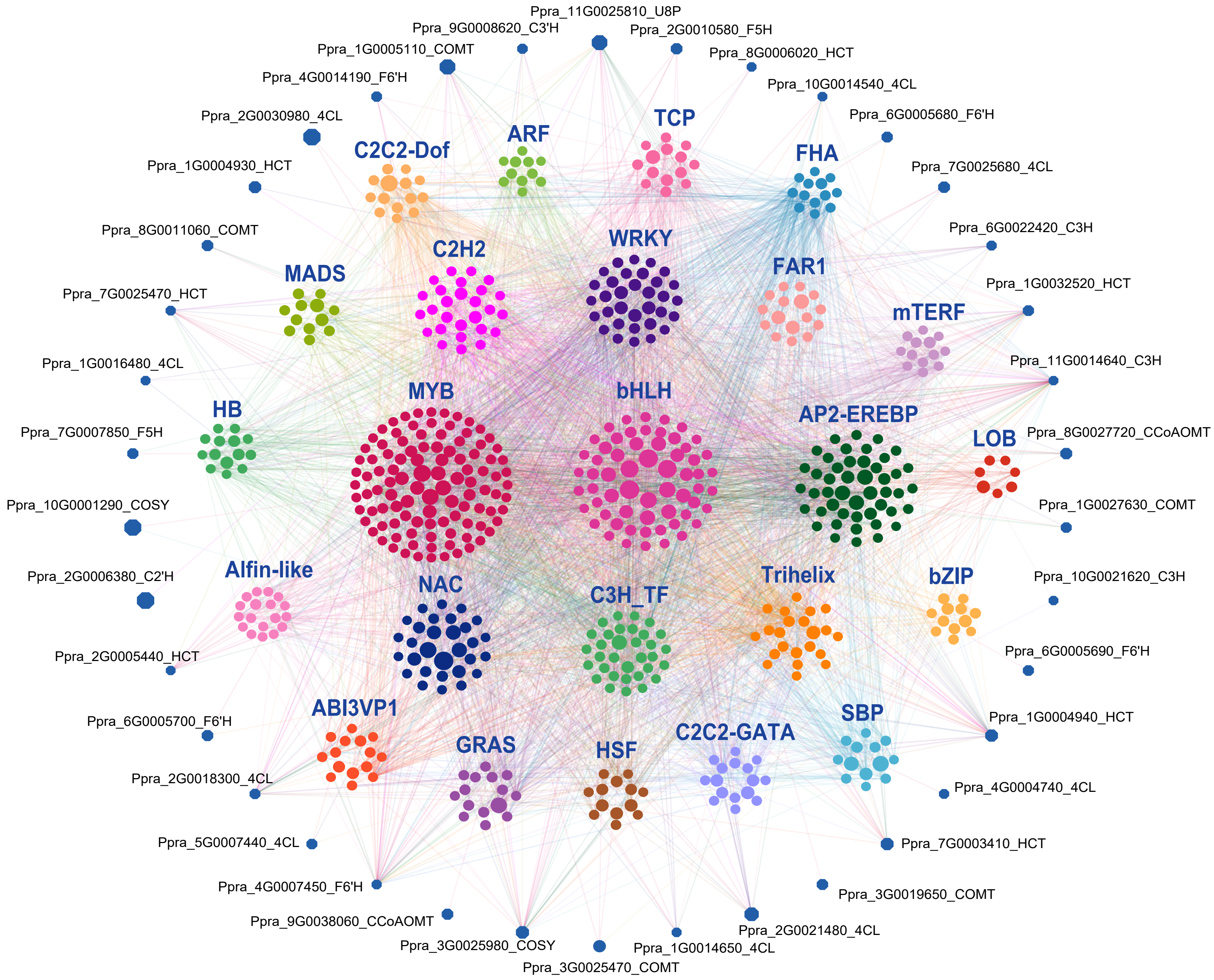

Supplementary Figures

Click here to access/download
Supplementary Material
Supplyment Figures.docx

Supplementary tables

Click here to access/download
Supplementary Material
Supplyment Tables data.xlsx
