# Supplementary material for: The telomere-to-telomere (T2T) genome of Peucedanum praeruptorum Dunn provides insights into the genome evolution and coumarin biosynthesis
Source: Gigascience. 2024 Jun 5;13:giae025. doi: 10.1093/gigascience/giae025 (PMC11152176; doi:10.1093/gigascience/giae025)
Supplement: giae025_GIGA_D_23_00282_Revision_2 [file giae025_giga_d_23_00282_revision_2.pdf]

GigaScience

The telomere-to-telomere (T2T) genome of Peucedanum praeruptorum Dunn provides insights into the genome evolution and coumarin biosynthesis

--Manuscript Draft--

**Manuscript Number:**	GIGA-D-23-00282R2
**Full Title:**	The telomere-to-telomere (T2T) genome of Peucedanum praeruptorum Dunn provides insights into the genome evolution and coumarin biosynthesis
**Article Type:**	Research
**Funding Information:**	Key Technologies Research and Development Program (2022YFD1201600) — Dr Shancen Zhao
**Abstract:**	Background<br>Traditional Chinese medicine has used Peucedanum praeruptorum Dunn (Apiaceae) for a long time. Various coumarins, including the significant constituents Praeruptorin (A-E), are the active constituents of the dried roots of P. praeruptorum. Previous transcriptomic and metabolomic studies attempted to elucidate the distribution and biosynthetic network of these medicinal-valuable compounds. However, the lack of a high-quality reference genome impedes an in-depth understanding of genetic traits and, thus, the development of better breeding strategies.<br>Results<br>A telomere-to-telomere genome was assembled for P. praeruptorum by combining PacBio HiFi, ONT ultra-long, and Hi-C data. The final genome assembly was approximately 1.798 Gb, assigned to 11 chromosomes with genome completeness >98%. Comparative genomic analysis suggested that P. praeruptorum experienced two whole genome duplication events. By the transcriptomic and metabolomic analysis of the coumarin metabolic pathway, we presented coumarins' spatial and temporal distribution and the expression patterns of critical genes for its biosynthesis. Notably, the COSY and cytochrome P450 genes showed tandem duplications on several chromosomes, which may be responsible for the high accumulation of coumarins.<br>Conclusions<br>A T2T genome for P. praeruptorum was obtained, it provides molecular insights into the chromosomal distribution of the coumarin biosynthetic genes. This high-quality genome is an essential resource for designing engineering strategies for improving the production of these valuable compounds.
**Corresponding Author:**	Henrik Toft Simonsen<br>Jean Monnet University: Universite Jean Monnet Saint-Etienne<br>SAINT-ÉTIENNE, FRANCE
**Corresponding Author Secondary Information:**	
**Corresponding Author's Institution:**	Jean Monnet University: Universite Jean Monnet Saint-Etienne
**Corresponding Author's Secondary Institution:**	
**First Author:**	Mingzhou Bai
**First Author Secondary Information:**	
**Order of Authors:**	Mingzhou Bai
	Sanjie Jiang
	Shanshan Chu
	Yangyang Yu
	Dai Shan
	Chun Liu
	Liang Zong

Powered by Editorial Manager® and ProduXion Manager® from Aries Systems Corporation

	Qun Liu
	Nana Liu
	Weisong Xu
	Zhanlong Mei
	Jianbo Jian
	Chi Zhang
	Shancen Zhao
	Tsan-Yu Chiu
	Henrik Toft Simonsen
**Order of Authors Secondary Information:**	
**Response to Reviewers:**	All the comments and suggestions from the reviewer was incoorporated. Also the full manuscript was rewise for the english langaure to provide a better flow in the sentenses through out.<br>We are gratefull for all the comments by the reviewers.<br><br>Yours truly,<br>Henrik Toft Simonsen
**Additional Information:**	
**Question**	**Response**
Are you submitting this manuscript to a special series or article collection?	No
**Experimental design and statistics**<br><br>Full details of the experimental design and statistical methods used should be given in the Methods section, as detailed in our Minimum Standards Reporting Checklist. Information essential to interpreting the data presented should be made available in the figure legends.<br><br>Have you included all the information requested in your manuscript?	Yes
**Resources**<br><br>A description of all resources used, including antibodies, cell lines, animals and software tools, with enough information to allow them to be uniquely identified, should be included in the Methods section. Authors are strongly encouraged to cite Research Resource Identifiers (RRIDs) for antibodies, model organisms and tools, where possible.	Yes

Have you included the information requested as detailed in our Minimum Standards Reporting Checklist?	
**Availability of data and materials**<br><br>All datasets and code on which the conclusions of the paper rely must be either included in your submission or deposited in publicly available repositories (where available and ethically appropriate), referencing such data using a unique identifier in the references and in the “Availability of Data and Materials” section of your manuscript.<br><br>Have you have met the above requirement as detailed in our Minimum Standards Reporting Checklist?	Yes

Powered by Editorial Manager® and ProduXion Manager® from Aries Systems Corporation

Manuscript
Click here to access/download;Manuscript;T2T-Peucedanum praeruptorum Dunn_gigascience May 1st.docx

The telomere-to-telomere (T2T) genome of *Peucedanum praeruptorum* Dunn provides insights into the genome evolution and coumarin biosynthesis

Mingzhou Bai[1,2, +], Sanjie Jiang[2, +], Shanshan Chu[3,4, +], Yangyang Yu[2], Dai Shan[2], Chun Liu[5], Liang Zong[6], Qun Liu[6], Nana Liu[7,8], Weisong Xu[2], Zhanlong Mei[2], Jianbo Jian[1,2], Chi Zhang[2], Shancen Zhao[2], Tsan-Yu Chiu[2,8 *], Henrik Toft Simonsen[9, *]

[1] DTU Bioengineering, Technical University of Denmark, Kongens Lyngby 2800, Denmark, mingbai@dtu.dk.

[2] BGI-Genomics, BGI-Shenzhen, Shenzhen 518000, China.

[3] School of Pharmacy, Anhui University of Chinese Medicine, Hefei 230000, China.

[4] Anhui Province Key Laboratory of Research and Development of Chinese Medicine, Hefei 230000, China.

[5] College of Tropical Crops, Hainan University, Haikou 570228, China.

[6] Wuhan BGI Technology Service Co., Ltd. BGI-Wuhan, Wuhan 430000, China.

[7] College of Pharmaceutical Science, Zhejiang University of Technology, Hangzhou, China.

[8] HIM-BGI Omics Center, Zhejiang Cancer Hospital, Hangzhou Institute of Medicine (HIM), Chinese Academy of Sciences (CAS), Hangzhou, China.

[9] Laboratoire Biotechnologies Végétales Plantes aromatiques et médicinales, Université Jean Monnet, St. Étienne 42023, France.

*Corresponding author: qiucanyu@genomics.cn and henrik.toft.simonsen@univ-st-etienne.fr.

[+] Contributed equally to the paper.

Mingzhou Bai [0000-0001-5666-8200];

Sanjie Jiang [0000-0002-7036-2803];

Shanshan Chu [0000-0003-1996-5593];

Zhanlong Mei [0000-0003-2203-2495];

Jianbo Jian [0000-0003-2187-5490];

Shancen Zhao [0000-0001-8779-6969];

Tsan-Yu Chiu [0000-0002-6622-0773];

Henrik Toft Simonsen [0000-0003-3070-807X]

Abstract

Background

Traditional Chinese medicine has used *Peucedanum praeruptorum* Dunn (Apiaceae) for a long time. Various coumarins, including the significant constituents Praeruptorin (A-E), are the active constituents of the dried roots of *P. praeruptorum*. Previous transcriptomic and metabolomic studies attempted to elucidate the distribution and biosynthetic network of these medicinal-valuable compounds. However, the lack of a high-quality reference genome impedes an in-depth understanding of genetic traits and, thus, the development of better breeding strategies.

Results

A telomere-to-telomere genome was assembled for *P. praeruptorum* by combining PacBio HiFi, ONT ultra-long, and Hi-C data. The final genome assembly was approximately 1.798 Gb, assigned to 11 chromosomes with genome completeness >98%. Comparative genomic analysis suggested that *P. praeruptorum* experienced two whole genome duplication events. By the transcriptomic and metabolomic analysis of the coumarin metabolic pathway, we presented coumarins' spatial and temporal distribution and the expression patterns of critical genes for its biosynthesis. Notably, the *COSY* and cytochrome *P450* genes showed tandem duplications on several chromosomes, which may be responsible for the high accumulation of coumarins.

Conclusions

A T2T genome for *P. praeruptorum* was obtained, it provides molecular insights into the chromosomal distribution of the coumarin biosynthetic genes. This high-quality genome is an essential resource for designing engineering strategies for improving the production of these valuable compounds.

Keywords

Peucedanum praeruptorum, T2T genome, coumarin biosynthesis

Background

Peucedanum praeruptorum Dunn (NCBI:txid312531) belongs to the plant family Apiaceae. Its dried root "Peucedani Radix" is used in traditional Chinese medicine, and the pharmacological activity is attributed to terpenoids and coumarins (e.g. Praeruptorin A-E) [1]. The root extracts of *Peucedanum praeruptorum* have been applied to treat headaches, coughing, and vomiting and have the potential to reverse multidrug resistances [1]. Among the active ingredients, coumarins are a class of compounds with a core structure that comprise of a fused benzene and α-pyrone ring. Generally, coumarins can be classified as: simple coumarins, furocoumarins, pyranocoumarins, phenylcoumarins, and biscoumarins [2,3]. Simple coumarins are widespread in various plant families, but the distribution of furanocoumarins is more limited. Furanocoumarins are identified in some plant families, including Apiaceae, Asteraceae, Moraceae, Pittosporaceae, Rosaceae, Rutaceae, Solanaceae, and Thymelaeaceae [4]. Linear furanocoumarins have been found in at least 19 plant families, with the majority found in Rutaceae and Apiaceae. The Apiaceae family, especially in the subfamily Apioideae, is a notable source of both linear and angular furanocoumarins [3,4]. While many plants synthesize linear furanocoumarins without angular counterparts, the production of angular furanocoumarins without linear ones is rare, suggesting a more recent evolution of angular biosynthesis [2,3]. However, no genetic evidences have supported this hypothesis yet.

The biosynthesis of the coumarin core structure is derived from phenylalanine. The phenylalanine is deaminated by phenylalanine ammonia lyase (PAL) to cinnamic acid and sequentially metabolised by cinnamate 4-hydroxylase (C4'H), 4-coumarate-coenzyme A (CoA) ligase (4'CL), *p*-coumaroyl-CoA 2′-hydroxylase (C2'H) to form umbelliferone [5,6]. Many

feeding studies have shown that umbelliferone is the precursor to form more complex coumarins (e.g. pyranocoumarins or furanocoumarins) [7].

The umbelliferone dimethylallyltransferases (UDT) are enzymes belonging to the prenyltransferases family. The UDTs perform specific prenylation at either the C6 or C8 position of umbelliferone, which then lead to linear or angular furano/pyranocoumarins, respectively [8]. Currently, through the analysis of both transcriptomic and metabolomic data, three distinct prenyltransferases (PpPT1-3) in *P. praeruptorum* have been identified as responsible for the prenylation of the simple coumarin skeleton, forming linear or angular precursors. Additionally, two novel CYP450 cyclases (PpDC and PpOC) have been shown to be responsible for the cyclization of these linear/angular precursors into either tetrahydrofurans or tetrahydropyrans [9]. Another recent study in *P. praeruptorum* also combined comparative transcriptomics and metabolomics to provide insights into transcriptional changes and the reduction of coumarins after blooming disclosing the key gene regulatory networks of coumarin biosynthesis in *P. Praeruptorum* at the vegetative growth stages and reproductive stages [10].

Comparative genomic analysis across species provides insights into understanding evolutionary relationships and the genetic basis of speciation. Several high-quality genomes in Apioideae have been published, including carrot [11], coriander [12], celery [13,14] and medicinal plants such as *Angelica sinensis* [15,16] and *Bupleurum chinense* [17]. A chromosomal-level genome of *P. praeruptorum* was very recently published [18]. Here, we independently assembled a Telomere to Telomere (T2T) genome of *P. praeruptorum* along with identifications of genes that coded for enzymes that are involved in the biosynthesis of the medically important coumarins. The genetic basis of these key traits in *P. praeruptorum* can provide a clear roadmap (e.g. gene clusters, regulatory elements) for future breeding or even the key biosynthetic gene discoveries for synthetic biology applications.

Methods

Plant materials and DNA/RNA isolation

The individual plants of *Peucedanum praeruptorum Dunn* (Apiaceae) were collected between April 2022 and November 2022 at the Anhui University of Chinese Medicine Garden in Heifei City, Anhui Province, China. The plant growth site was situated amidst the Huai and Yangtze Rivers, commonly called the Jianghuai area. Fresh, young, and healthy leaves were harvested for the extraction of high-molecular-weight genomic DNA using a modified cetyltri-methylammonium bromide (CTAB) method and nuclei method, respectively, for short reads and long reads (PacBio and Nanopore ultra-long) sequencing. Samples from leaves, stems, roots, flowers, and fruit tissues at three different growth stages were utilized for RNA extraction employing a RNeasy PowerWater Kit (Qiagen, Carlsbad, CA, USA).

Library preparation and sequencing

The quality control and quantity assessment of the isolated DNA was conducted using a NanoDrop 2000 (Thermo Scientific, CA, USA) and a Qubit 2.0 Fluorometer (Life Technologies, CA, USA). Following purification with the Qiagen genomic kit (Qiagen, 13343), approximately 5 μg of *P. praeruptorum* DNA was utilized for constructing short DNA insert size (~350 bp) libraries using the MGIEasy Universal DNA Library Prep Kit and generating 20 kb PacBio HiFi sequencing libraries with the SMRTbell Prep Kit 2.0. Subsequently, short libraries were sequenced on an DNBSEQ-T7 (RRID:SCR_017981) sequencing platform with 150 bp paired-end reads. The SageHLS HMW library system (Sage Science, USA) was utilized to select approximately 10 μg of gDNA with a size of about 100 kb for the construction of an ultra-long Nanopore library using the ONT 1D Sequencing Kit (SQK-LSK109). PacBio HiFi

sequencing and ultra-long ONT libraries were performed on the PacBio SequeII platform (RRID:SCR_017990) and Nanopore PromethION sequence (RRID:SCR_017987). SMRT cell subread was generated and processed using the CCS algorithm of SMRTLink (v8.0.0) [19,20]. The MGIEasy RNA Directional Library Prep Kit (MGI) was utilized to construct RNA libraries, with approximately 1-2 μg of total RNA from each tissue sample employed. Subsequently, all libraries were subjected to sequencing on DNBSEQ-G400 (RRID:SCR_017980), generating 150 bp paired-end reads.

The Hi-C library was prepared to facilitate the anchoring of assembled contigs to chromosomes through the following steps. The fresh young leaves were cross-linked using formaldehyde (Sigma), followed by resuspension in lysis buffer. Chromatins were fragmented using MboI (NEB) restriction endonucleases. Biotin labelling was performed, and cross-linking was achieved using T4 DNA Ligase (ENZYMATICS). The captured fragments were isolated using Streptavidin-coated magnetic beads (ThermoFisher SCIENTIFIC). An "A" base was added at the 3'-end of each strand using the KAPA HYPER PREP KIT (KAPA). After purification, the Hi-C library was sequenced with PE150 in the DNBSEQ-T7 sequencing platform.

Genome survey and *de novo* assembly

A pilot genome survey was performed prior to the long read (PacBio and ONT) sequencing. This was done to establish a cost-effective strategy. With the 150-bp short reads, Jellyfish [21] and Genomescope (RRID:SCR_017014) 1.0 were used to predict the genomic characteristics [22]. The genome size and heterozygosity rate of the *P. praeruptorum* were determined through Kmer analysis. This showed that PacBio, Hi-C and ONT could be done on the extracted DNA. With the combination of PacBio HiFi, Hi-C, and ONT Ultra-long data (with a length of 100kb or more), an initial *P. praeruptorum* contig assembly was performed by Hifiasm (RRID:SCR_021069) (Version 0.19.5) with default parameters to obtain the draft genome [23].

Purge_haplotigs (RRID:SCR_017616) (Version 1.0.4, parameter: -a 70)[25] was applied to identify possible hybrid sequences in the draft genome based on sequence similarity and read coverage depth, such hybrid sequences was removed prior to genome anchoring according to the interaction and depth conditions. To identify possible contamination and plastid (chloroplasts and mitochondrial) sequences, BLASTN (RRID:SCR_001598) (Version 2.11.0+, parameters: -evalue 0.00001 -max_hsps 1) was used to perform NT alignment on the draft genome to identify such sequences. These were removed prior to the genome anchoring.

Following the “clean-up” of the sequence data, Juicer (RRID:SCR_017226) (Version 1.6, parameter: default) [20] was used to align the Hi-C data to the draft genome, and 3D-DNA (Version 180922, parameter: -r 0) [24] was used for preliminary anchoring. JuiceBox (RRID:SCR_021172) (Version 1.11.08)[26] was applied to visualize the 3D-DNA results for manual error correction, generating a 'chromosome genome.' The ONT ultra-long reads were aligned to the 'chromosome genome' sequences using minimap2 (Version 2-2.24) [27] and the gap filing was facilitated by TGS-GapCloser (RRID:SCR_017633) (Version v1.2.0, parameter: --min_nread 10) [28]. All reads aligned once within 100 bp at the end of the chromosome were collected, and the reads containing artefact sequences were filtered out. The read with the median extendable length was defined as ref and the others as a query. Medaka_consensus (Version 1.7.2, parameter: -ax map-ont) [29] was applied to reassemble the ref telomere and the query telomere to get the consensus sequences.

The consensus sequences (more than four repeat units) were aligned to both ends of each chromosome by BLASTN (Version 2.11.0+) [30] according to the positional relationship in the alignment. The telomere sequence was replaced with the aligned sequences at coverage >=90. The gap-free genome sequence was obtained, and error correction was performed on short reads using Pilon (RRID:SCR_014731) (Version 1.23, parameters: --fix snps, indels) [31]. The distribution of all repeat categories was investigated. The LINE/L1

distribution is consistent with the centromere distribution of *P. praeruptorum*, and this region is also located in a low-gene region. The completeness of the new genome was assessed using BUSCO (RRID:SCR_015008) (Version 5.1.2) with the embryophyta_odb10 database, which comprises 1,614 conserved core eukaryotic genes [32].

Genome annotation

The newly gap-free assembled genomes of *P. praeruptorum* were utilized to annotate repetitive elements and genes. The annotation of repetitive sequences is performed using a combination of methods. Firstly, *de novo* prediction based on features of repeated sequences utilizing TRF (Version 4.09) was performed [33]. Secondly, a homology-based prediction method employing RepeatMasker (RRID:SCR_012954) (Version open-4.0.9) was utilized [34] based on repeat database [35], followed by the construction of a custom library for repetitive sequence features using RepeatModeler (RRID:SCR_015027) (Version open-1.0.11v2.0) [36] and LTR_FINDER (Version 1.0.7) [37]. *De novo* predictions were performed through RepeatMasker (Version open-4.0.9) [34].

The prediction of the gene set was conducted by integrating different methods, including homologous prediction based on homologs from nine closely related species (*Angelica sinensis*, *Apium graveolens*, *Aralia elata*, *Coriandrum sativum*, *Daucus carota*, *Eleutherococcus senticosus*, *Oenanthe sinensis*, *Panax ginseng*, *Panax notoginseng*) using as Exonerate (RRID:SCR_016088) (Version 2.2.0) [38] and Liftoff (Version 1.6.3) [39]. *De novo* prediction based on ab initio approaches was also employed, including AUGUSTUS (RRID:SCR_008417) (Version v3.2.3) [40] and GlimmerHMM (RRID:SCR_008417) (Version 3.0.4) [41], and the Transcriptome-based prediction was performed using RNAseq data. A total of 198Gb of the 33 samples of newly sequenced RNAseq data were mapped to the newly assembled genome sequences using HISAT2 (RRID:SCR_015530) (Version 2.1.0) [42]. Stringtie

(RRID:SCR_016323) 2.1.6 [43] was employed for transcript identification and transcript-assisted annotation. Finally, the gene set of *P. praeruptorum* was integrated from the different methods by implementing the MAKER pipeline (RRID:SCR_005309) (v3.31.8) [44]. For non-coding RNA, tRNAscan-SE (RRID:SCR_008637) (Version 1.3.1)[45] was used to identify tRNA sequences in the genome based on the structural characteristics of tRNA. Since rRNA is highly conserved, rRNA sequences of the closely related species mentioned above were selected as reference sequences, and BLASTN alignment was used to identify rRNA. MiRNA and snRNA sequences were annotated by the covariance model of the Rfam family and INFERNAL that comes with Rfam (RRID:SCR_007891) (Version 14.8) [46]. The completeness of the genome annotation was assessed using BUSCO (Version 5.1.2) [32] with the embryophyta_odb10 database.

Gene family and phylogenomic analysis

The genome sequences of *P. praeruptorum* and ten representative plants (*Angelica sinensis, Apium graveolens, Arabidopsis thaliana, Coriandrum sativum, Daucus carota, Oryza sativa, Panax notoginseng, Populus trichocarpa, Theobroma cacao, Vitis vinifera*) were utilized for gene family clustering and phylogenetic analysis. The gene sets of the 11 species included in the analysis were processed as follows. In cases where multiple transcripts of a gene (resulting from variable splicing) existed in the annotation files, only the longest transcript was retained. Genes encoding proteins with fewer than 30 amino acids or genes containing internal stop codons were excluded. The protein sequence similarity among all species was determined using an all-vs-all BLASTP (RRID:SCR_001010) (e-value 1e-5) approach, followed by gene family clustering using OrthoMCL (RRID:SCR_007839) (Version 2.0.9) [47]. A total of 489 single-copy orthologous genes were identified, and multi-sequence alignment of coding sequences was aligned using MAFFT (RRID:SCR_011811) (Version 7.487) [48]. This was followed by

a single-copy supergene filtered with a minimum corresponding amino acid length of 100. The conserved sites were obtained using the default parameters of Gblocks (RRID:SCR_015945) (Version 0.91b) [49], followed by the construction of a phylogenetic tree using RaxML (RRID:SCR_006086) (Version 8.2.12) [50] with parameter (-fa -N 100 -m GTRGAMMA). The divergence time of 11 target plant species was inferred using MCMCtree of PAML (RRID:SCR_014932) (Version 4.9j) [51], incorporating two calibrated divergence time intervals: Oryza sativa - *Vitis vinifera* (163.5-142.1 Mya) and *Panax notoginseng–Daucus carota* (69.0-54.3 Mya byTimeTree (RRID:SCR_021162). The gene family expansion and contraction of 11 species were identified using the CAFE (Version 4.2) pipeline [41], and the gene families were subjected to KEGG and GO enrichment analysis to elucidate their functional roles. The collinearity of genome and whole genome duplication (WGD) events were analyzed by the WGDI pipeline [53]. Firstly, the protein sequences of *P. praeruptorum* were compared to those of three other species (*Angelica sinensis*, *Coriandrum sativum*, and *Daucus carota)* through all-vs-all BLASTP analysis with an e-value threshold set at 1e-5. Subsequently, gene location information and chromosome length data were extracted. The syntenic blocks' synonymous substitution rate (Ks) were used to plot the dot.

RNA-Seq Data Analysis

The experiment involved a total of 33 samples, including three different tissue types at different growth stages: Vegetative growth stage (VP) - root, stem, leaf; Flowering stage (AP) - root, stem, leaf, flower; Fruiting stage (FP) - root, stem, leaf, fruit (Figure 1). Three biological replicates represented each tissue type. The low-quality raw reads of each sample were processed firstly using SOAPnuke (RRID:SCR_015025) (Version 1.5.2) [54]. The clean reads were aligned to the newly assembled references genome and gene sequence using HISAT (RRID:SCR_015530) (Version 2.1.0) [42] and Bowtie2 (RRID:SCR_016368) (Version 2.4.5)

[55], respectively. The gene and transcript expression levels were quantified using RSEM (RRID:SCR_000262) (Version 1.2.8) [56]. Differentially expressed genes (DEGs) were calculated by DESeq2 (RRID:SCR_015687) [57]. The co-expression network was constructed using the WGCNA (RRID:SCR_003302) (Version 1.71) package in R [58]. The phenotypic data (the value of specialized metabolites in each tissue) were utilized and imported into the WGCNA framework, enabling the calculation of correlation-based associations between them. Subsequently, the adjacency matrix was transformed into a topological overlap matrix using WGCNA. The node and edge datasets were imported into cytoscape (RRID:SCR_003032) (Version 3.10.0) [59] for the final figure. The genes (e.g., phenylalanine ammonia-lyase (PAL), cinnamate 4-hydroxylase (C4H), cinnamate 3-hydroxylase (C3H), CoA O-methyltransferase (COMT), 4-coumarate-CoA ligase (4CL), *p*-coumaroyl CoA 2'-hydroxylase (C2'H), coumarin synthase (COSY), shikimate hydroxycinnamoyl transferase (HCT), *p*-coumaroyl 5-O-quinate/shikimate 3'-hydroxylase (C3'H), caffeoyl-CoA O-methyltransferase (CCoAOMT), feruloyl-CoA 6'-hydroxylase (F6'H), glucose 6-phosphate (U-6-P), and glucose 6-phosphate (U-8-P)) involved in the coumarin biosynthesis in *P. praeruptorum* were identified by using the genes from *A. Sinensis*, *A. thaliana, Z. officinale, and P. sativa* as a query. The genes with identity >=80 and coverage >=70% were selected. The heatmap of their expression patterns was displayed by pheatmap (RRID:SCR_016418) (Version 1.0.8) in.

Quantitative PCR (qPCR)

Quantitative PCR (qPCR) was employed to assess mRNA transcription levels for PpPT1/PpPT2/PpOC/PpDC. The assay utilized ensured unbiased amplification of the prevalent alleles at each locus, excluding amplification of all other loci. The primers used for amplification were listed in Supplementary Table 23. Reverse transcription was conducted using the HiScript Ill All-in-one RT SuperMix Perfect for qPCR (R333-01) from Vazyme.

Amplification of candidate genes and the reference gene SAND cDNA was carried out using Pro Universal SYBR gPCR Master Mix (Vazyme) on a QuantStudio 3 Real-Time PCR Systems (Thermo Fisher Scientific). The average expression level of each gene was normalized to that of SAND and calculated using the 2-ΔΔCt method, where Ct represents the threshold cycle [60]. In correlation analysis between the log2 Fold Change (log2FC) (RNA-seq) and log2FC (qPCR), the linear regression r^2 (goodness-of-fit) is reported.

Metabolic analysis

A total of 66 tissues were collected for metabolic analysis. Per the transcriptome samples, each tissue was represented by six biological replicates. The metabolite profiling from each tissue was performed using a nontargeted metabolomics approach, following the established protocol by Tohge and Fernie (2010) [61]. High-resolution mass spectrometry (HRMS) was conducted using an ultraperformance liquid chromatography (UPLC) system followed by The Q Exactive™ Plus Hybrid Quadrupole-Orbitrap™ Mass Spectrometer (MS). The base peak chromatogram (BPC) was utilized to represent a continuous depiction of the highest ion intensities recorded at each time point. All quality control samples were superimposed in positive and negative ion modes, demonstrating excellent stability and high-quality data obtained from the instrument detection process. The differential metabolites between the two biological groups were screened using univariate and multivariate analyses with a VIP ≧ 1, Fold Change ≧ 1.2 or ≦ 0.83, and q-value <0.05.

Analysis of coumarin biosynthesis-related cytochrome P450 gene family

The coumarin biosynthesis-related cytochrome P450 gene family with CYP71A and CYP82C was used as a query to blast the *P. praeruptorum* genomes with the similarity of 70% as the cutoff. A phylogenetic tree of these members was constructed using the adjacency method of

RAxML (Version 8.2.0)[50]. The chromosome location of these identified cytochrome P450 genes was labeled, and the gene structure was visualized using TBtools (RRID:SCR_023018) (Version 2.019) [62].

MEME (RRID:SCR_001783) (Version 5.5.5) was used to predict the motifs of these CYP genes, and the number was set to 10 [63]. The promoter region of each CYP gene with a length of 2000 bp was obtained from the genome, and cis-acting regulatory elements were predicted according to the PlantCARE database [64]. The predicted cis-elements were divided into seven functional categories: common, light, hormone, stress, development, other, and flavonoid biosynthesis. A statistical histogram of the number of cis-elements was generated.

Results

Genome assembly and genome annotation

The preliminary genomic information of *P. praeruptorum* (Figure 1A) estimated a genome size of 1.78 Gb, and the heterozygosity rate was calculated to be 1.3 % (Supplementary Figure S1 and Data Table S1). Further, we applied PacBio HiFi sequencing (72.6 Gb), ONT Ultra-Long DNA sequencing (349.7 Gb), and Hi-C sequencing (533 Gb) to yield highly accurate long-read sequencing datasets (Supplementary Data Table S2). After initial assembly by hifiasm, a 1.86 Gb size draft genome was generated with a GC content of approximately 35.5% (Supplementary Figure S2, Supplementary Data Table S3). A chromosome-level genome assembly (hereafter named 'chromosome genome') with Hi-C alignment yielded a 1.8 Gb genome with contig N50 148.7 Mb. Then, the ONT Ultra-Long sequences were used to fill the gaps of the 'chromosome genome,' thereby a telomere-to-telomere level genome was generated consisting of 11 chromosomes (Figures 1B and 1C). This nearly gapless genome consists of 253 contigs, N50 of 161 Mb, and a GC content of 35.5% (Supplementary Data Table S3). The telomeres and centromeres were identified and characterized (Supplementary Data Table S4

and S5). The final HiC-heatmap of this T2T genome is presented in Figure 1D. The short-read and long-read data were mapped to the newly assembled sequences using BWA [65] and minimap2 [27]; this allowed evaluation of the accuracy of assembly sequences. Respectively, the 99.73% mapping rate and 99.91% coverage rate with depth >4 showed a high consistency between assembly results and reads; it also plotted the GC content and depth distribution for analyzing the sequencing uniformity (Supplementary Figure S3). Furthermore, the Benchmarking Universal Single-Copy Orthology (BUSCO) analysis showed that the assembled genomes exhibit a completeness of more than 98.2% identified in the "eukaryote_odb10" database (Supplementary Data Table S6).

A total of 1.07 Gb of repeat sequences were detected, accounting for 59.67% of the assembled genome (Supplementary Data Table S7). This repeat content was less than the value (79.3%) predicted by the k-mer analysis (Supplementary Data Table S1). The most abundant transposable elements were long terminal repeats (LTR), which account for 49.02% of the genome (Supplementary Data Table S8). A total of 247,398 and 164,100 protein-coding genes were *de novo* predicted using the GlimmerHMM and AUGUSTUS, respectively (Supplementary Data Table S9). Nine well-assembled plant genomes in Apiales, including the Apiaceae species *Angelica sinensis, Apium graveolens, Coriandrum sativum, Daucus carota,* and *Oenanthe sinensis*, and the Araliaceae species *Aralia elata, Eleutherococcus senticosus, Panax ginseng*, and *Panax notoginseng* were used for homologous prediction. The predicted genes were integrated into a non-redundant, more complete gene set with 53,756 protein-coding genes by MAKER2 [66] (Supplementary Figure S4-S6). A final reliable set of 44,468 high-confidence genes was obtained using the in-house script (Supplementary Data Table S9 and S10). The gene function of the protein-coding genes was defined by the following databases: NR (94.18%), SwissProt (56.85%), TrEMBL (93.83%), KOG (65.75%), TF (5.96%), InterPro (77.48%), GO (57.43%), KEGG_ALL (85.47%), KEGG_KO (32.73%) and

Pfam (69.61%) (Supplementary Figures S7-9). A total of 95.4% of protein-coding genes were annotated (Supplementary Data Table S11). We also annotated the non-coding RNAs and acquired 181 miRNAs, 2359 tRNAs, 6879 rRNAs, and 8823 snRNAs (Supplementary Data Table S12). Furthermore, BUSCO analysis showed that the annotated genes exhibit 97.3% completeness in the database (Supplementary Data Table S6). Compared with the newly published *P. praeruptorum* genome [18], the data from this study demonstrated high quality in terms of assembly and annotation (Supplementary Data Table S13 and S14, Supplementary Figure S10).

Evolutionary analysis

A phylogenetic tree was constructed to estimate the divergence time of *P. praeruptorum* and ten other representative plant species (Figure 2A and Supplementary Figure S11). *P. praeruptorum* belongs to the Order of Apiales; it diverged from the other plant orders approximately 113.6 million years ago (Mya). Within the Apiales, *P. praeruptorum* clustered with its relatives in the Apiaceae family, which diverged from the Araliaceae family member *Panax notoginseng* about 62.5 Mya (Figure 2A). In total, 725 gene family contractions and 913 gene family expansions were detected in *P. praeruptorum.* Functional enrichment analysis was performed for those expansion and contraction genes (Supplementary Figures S12-S15). The genome of an organism is a dynamic landscape, shaped by evolutionary forces and environmental pressures. The expansion of these gene families contributes to the resilience and adaptability of organisms, allowing them to exploit diverse ecological niches. The top three expanded gene families were enriched in the GO terms of monooxygenase activity, oxidoreductase activity, and iron ion binding respectively (Supplementary Figures S14), and in the KEGG pathways of photosynthesis, spliceosome, and protein processing in endoplasmic reticulum (Supplementary Figures S15). It is worth mentioning that the secretion of coumarins,

phenolic secondary metabolites deriving from the general phenylpropanoid pathway, is a common approach induced by iron starvation and is thought to mobilize the recalcitrant iron pools. Interestingly, the expansion of gene families associated with monooxygenase activity, oxidoreductase activity, and iron ion binding signifies an enhanced capacity for *P. praeruptorum* to synthesize diverse coumarins, engage in crucial redox reactions, and efficiently manage iron acquisition in nature. In contrast, the top 2 contracted gene families were enriched in the GO terms of pathways of intramolecular transferase activity and hydrolase activity, and hydrolysing O-glycosyl compounds (Supplementary Figures S12), and in the KEGG pathways of galactose metabolism and sucrose metabolism (Supplementary Figures S13).

The enrichment of single-copy and multiple-copy genes was analysed in 11 plant species to investigate species-specific gene families, common gene families, homologous genes, and gene family clusters. (Figure 2B). *P. praeruptorum* carries 44,468 genes, clustered into 19402 gene families containing 489 single copy gene families (Figure 2B and Supplementary Data Table S15). Among the gene families, 4016 common gene families were shared with other plant species, and 741 gene families were specific to *P. praeruptorum* (Figure 2C). We further performed collinearity analysis between *P. praeruptorum, D. carota, C. sativum,* and *A. sinensis.* The results showed a few major chromosomal rearrangements occurred between those species (Figure 2D and Supplementary Figure S16). The distribution of synonymous substitutions per synonymous site (*Ks*) for Apiaceae plants was compared. Two major peaks were observed, consistent with the previous hypothesis that *P. praeruptorum* experienced two WGD events (Figure 2E).

As the first species to be separated in our phylogeny in the Apiaceae, *D. carota* harbours nine chromosomes in a haploid. In contrast, many other members (e.g., *A. graveolens*, *A. sinensis*, *C. sativum*, *P. praeruptorum*, *Thapsia garganica*, *Thapsia smittii*) all have 11 chromosomes in

a haploid (Supplementary Figure S17)[13,67,68]. The complete dot-plot-based deconvolution into 11 reconstructed Conserved Ancestral Regions (CARs) of the observed synteny and paralogy among *P. praeruptorum* and its Apiaceae siblings suggested the 11 proposed protochromosomes as the origin of Apiaceae. Our analysis is also consistent with the previous report that modern celery chromosomes are well represented by the Apiaceae protochromosomes (Supplementary Figure S17)[13]. In addition, comparing *D. carota* with the other Apiaceae members with 11 chromosomes indicated that chromosome 10 and chromosome 6 experienced fission and fusion that reduced chromosome numbers in *D. carota* (Supplementary Figure S17).

Biosynthesis of coumarins

The biosynthesis of coumarins is initiated at the phenylpropanoids pathway whereas the L-phenylalanine is catalysed by PAL to form cinnamic acid (Fig 3A). The cinnamic acid is further converted to *p*-coumaric acid by C4H and transformed into *p*-coumaroyl CoA by a member of the 4CL family. The CoA-esters are subsequently hydroxylated at the position ortho to the aromatic ring aliphatic side chain through either C2'H. The coumarin core structure (e.g. umbelliferone in the roots is catalysed by Coumarin Synthase (COSY) [59]. For *P. praeruptorum* the biosynthetic genes (*PAL*, *C4H*, *4CL*, *C2'H*) in the initial steps of phenylpropanoid pathway showed diverse spatial and temporal expression patterns since these compounds are common precursors for downstream anthocyanins, lignin and flavonoids pathways [69](Supplementary Figure S19). The genes (*C2'H*, *COSY*, *U-8-P* and *U-6-P*) that are involved in the formation of umbelliferone and its derivatives, are mainly expressed in the roots and stems at the vegetative stage (Supplementary Data Table S18 andS19). However, during the reproductive stage (e.g. anthesis and fruit), these transcripts showed reduced expression, which is also supported by the metabolomic data (Fig 3B). The metabolomic

analysis showed that the major forms of coumarins could be detected in the roots during the growth stages (Figure 3B). This was consistent with previous reports where coumarins were secreted into the rhizosphere due to their allelopathic properties or to be involved in iron acquisition [64]. A few coumarins such as skimmin, rutarin, isopropylidenylacetyl-marmesin, isobergapten and decursinol were mainly observed in the leaves (Figure 3B), but in general the coumarins could be found in the roots and stems (Supplementary Data Table S20).

Coumarin Synthase (COSY)

Previously, biochemical and molecular experiments done in the *Ruta graveolens* and Arabidopsis suggested that the trans–cis isomerization and lactonization forming the coumarin core structure was a spontaneous reaction catalyzed by lights [70–73]. However, a BAHD family member named coumarin synthase (COSY) was cloned in Arabidopsis and was demonstrated to catalyze the reaction without light (Figure 3). Since COSY is a key gene for the ring closing in coumarin skeleton, we specifically examine their expression and evolution in Apiaceae. COSY is mainly expressed in the roots, an organ away from lights, and is a conserved gene across many plant species[74]. We used four *A. sinesis* COSY (*AS10G01653*; *AS02G01453*; *AS10G00118* and *AS11G01965*) as the query to identify COSY genes in two other Apiaceae plants (*P. praeruptorum* and *D. carota*) (Supplementary Data Table S21). Phylogenetic analysis of plant COSY enzymes showed that there are five major clades. Interestingly there is a multicollinearity comparison between *P. praeruptorum* and *A. Sinensis*, and there is one more copy of *COSY* in *P. praeruptorum* on chromosome 10, which occurred by tandem duplication after *P. praeruptorum* diverged from *A. Sinensis* (Figure 3C and Figure 2A). The expression patterns of this pair were slightly different at developmental stages. The expression of *Ppra_10G0001290* was downregulated at the anthesis and the fruiting periods

(Figure 3A). This suggests that these two genes may play different roles in coping with developmental needs. However, the detailed functions still need to be clarified.

The biosynthesis of complex coumarins following the umbelliferone has recently been described in *P. praeruptorum*. Prenylation of the umbelliferone carbon skeleton 6 or skeleton 8, followed by subsequent cyclization, are regarded as crucial steps to form furanocoumarins or pyranocoumarins. These steps play roles in determining the linear or the angular structures of either furanocoumarins or the pyranocoumarins [75]. Seven prenyltransferases (PT) in total were identified based on the transcriptome and metabolome data analysis and six out of seven prenyltransferases with catalytic activities of prenylating the umbelliferone were characterized (Supplementary Figure S20). PpPT1 (ON934685) has a umbelliferone 6-prenyltransferase (U6P) activity and PpPT2 (ON934686) has a umbelliferone 8-prenyltransferase (U8P) activity with a minor U6P activity. PpPT3 (ON934687) has both U6P and U8P activities, whereas the rest of the three homologs have weak U6P activities [9]. Additionally, two CYP P450 monooxygenases PpDC (ON934691) and PpOC (ON934692) have been identified [9] .

Genes that play role in the production of specific metabolites are frequently grouped in clusters to enable synchronized expression, a phenomenon commonly in plants and described for many different specialised metabolites [76–80]. Thus, we used the sequence information provided above and blasted the genome we had in hand. Interestingly, all the three major PTs (PT1-3) are located on the chromosome 11 (Fig 3D). Additionally, the *PpOC* (*ON934692*) is located on chromosome 11 and forms a functional gene pair with *PpPT2* (*ON934686*). We looked into the details of gene annotations in this region and identified other three gene pairs comprised of *PT* and *C'2H*. Frequently, gene pairs usually undergo tight regulation together at the nucleosome level and are co-expressed together [81]. We checked the expression pattern between these gene pairs. Notably, the *PpOC* (*ON934692*) and *PpPT2* (*ON934686*) display similar expression patterns in spatial and temporal manners (Figure 3E). The expressions of

PT1/PT2/PpOC/PpDC were double-checked with qPCR and showed high correlations with the transcriptome (Supplementary Figure S19). The other three pairs also showed similar expression except for the gene pair of *Ppra_11G0025740* and *Ppra_11G0025750*. These two genes displayed similar root-specific expression but with opposite temporal expression patterns (Figure 3E). Here, we demonstrated how a well-assembled genome could provide a roadmap to pathway elucidation. However, how these gene pairs function in the coumarin biosynthetic pathway requires further exploration.

Cytochrome P450 genes in coumarin biosynthesis

Plant cytochrome P450s catalyze several regio- and stereo-specific hydroxylations that play important roles in the general and specialized metabolites biosynthesis [82–84]. Based on the radioactive labeling of *Ammi majus* cell cultures, it is suggested that the cytochrome P450s are involved in the coumarins biosynthesis [85]. Thus, to identify the functional genes, we initially screened all the genes within the CYP71 family in the *P. praeruptorum* genome. This approach was based on the hypothesis that the isopentene group cyclization mechanism shares similarities with the CYP71 menthofuran synthase derived from *Mentha piperita*. Moreover, several members of the CAYP71AJ subfamily have been cloned and characterized [86,87]. The *AmCYP71AJ1* cloned from *A. majus* (Apiaceae) is responsible for catalysing the linear furanocoumarins formation [88]. In contrast, the *PsCYP71AJ4* from *Pastinaca sativa* is an angelicin synthase, which is an angular furanocoumarin and that have been modelled as well [87,89]. Additionally, we analyzed a total of 48 cytochrome P450 genes by homology comparison with CYP71AJ gene family members of *A. sinesis* (Figure. 4). The phylogenetic tree demonstrated that these genes were distributed into three major lineages (e.g., *PpCYP71AJ*, *PpCYP71AZ*, and *PpCYP82C*). Interestingly, the members of *PpCYP71AZ* and *PpCYP82C* are expanded significantly in *P. praeruptorum* when compared with other Apiaceae members (e.g.,

A. sinensis, *C. sativum*, and *P. notoginseng*) (Figure 4D and Supplementary Data Table S22). These expanded genes may be responsible for catalyzing the successive regio- and stereo-specific hydroxylation in the complex coumarin biosynthesis. This result is consistent with the diverse coumarins detected in the *P. praeruptorum* (Figure 3 and Supplementary Data Table S20). Based on the gene annotations, these CYP genes all shared similar gene structures, which contain two main CDS and three major motifs in their cis-elements (Figures 4A and 4B). In addition to the common cis-elements, light, hormone, and stress-responsive elements were the three major types. This suggests that the whole gene duplication events are the major drive to expand this gene family. Several tandem and proximal duplications were observed except on chromosomes 5, 8, and 10. Interestingly, no *PpCYP71* nor *PpCYP82* members can be identified on chromosome 5 (Figure 4E).

The systematic regulation of coumarin biosynthesis

Since the cis-elements of *PpCYP71AJ* members showed diverse regulatory elements, we developed a systematic view of the regulatory network of coumarin biosynthesis and the potential transcription factors (TFs) that are associated with these genes. A co-expression network connecting key node genes in coumarin biosynthesis with TFs was analyzed. The expression patterns of *C3H* (*Ppra_11G0014640*), *4CL* (*Ppra_2G0018300*, *Ppra_2G0018300*), *F6'H* (*Ppra_4G0007450*), and *COSY* (*Ppra_3G0025980*) were found to be highly related to the expression patterns of numerous TFs (Figure 5). The MYB, bHLH, AP2-EREBP, and WRKY were the four major TFs regulating coumarin biosynthesis. The R2R3-MYB and bHLH were well known that they, together with WD40, form ternary complexes that positively or negatively regulate flavonoid biosynthesis genes [90,91]. The simple coumarin scopolin was known to be accumulated under abiotic stress conditions [92]. Based on our results, the AP2-

EREBP and WRKY were biotic and/or abiotic stress response-related TFs, which further reflected the biosynthesis of these diverse coumarins to cope with various stress conditions.

Terpene biosynthesis

Terpenoids, a class of natural products that are rich in herbal plants, have been widely studied for their therapeutic efficacy especially in Apiaceae [93–95]. The distribution of terpenoids was examined in the root/stem and leaf with three different developmental stages. These compounds are widely distributed in the various tissues in *P. praeruptorum* (Supplementary Figure S18C). To identify the key TPS genes involved in the production of major terpenoids, we conducted a comprehensive analysis of the *P. praeruptorum* genome. The full-length *PpTPS* genes were obtained through genome scanning (Supplementary Figure S18). A total of 48 *PpTPS* genes were identified and categorized into six subfamilies (*PpTPS-a*, *b*, *c*, *e*, *f* and *g*) based on phylogenetic analysis, following the previously established nomenclature (Supplementary Data Table S16) [96]. It is worth mentioning that the *TPS-a* and *TPS-b* subfamilies have the largest number of members (Supplementary Figure S18B). Specifically, the *PpTPS-b* subfamily, which encodes angiosperm-specific monoterpene synthases, was substantially expanded with 24 members (Supplementary Figure S18B, Supplementary Data Table S17). Most of the TPSs are expressed during the vegetative stages in the root, the stem, and the leaf (Supplementary Figure S18B). By analysing their chromosomal localization, the distribution of *PpTPS* genes is across all 11 chromosomes. Chr04 hosts the greatest abundance of *TPS* genes, amounting to a total of 12 genes. Among them, ten genes have formed pairs through tandem duplication events (Supplementary Figure S18D).

Discussions

P. praeruptorum is a valuable Chinese medicinal plant commonly used to treat coughing and as an anti-mucus agent. Among the bioactive compounds, coumarins show high bioactivity to reduce multi-drug resistance in cancer cells with low toxicity [97]. Thus, *P. praeruptorum* is considered a great resource for isolating these compounds. High-quality reference genomes could help uncover important traits and elucidate key catalytic enzymes for synthetic biology. A chromosome-level genome assembly of *Artemisia annua* revealed the artemisinin content is correlated to the copy number of amorpha-4,11-diene synthase genes, as one representative example of how genomic information can help to improve plant-specific metabolism [98]. Here, we present the first T2T genome of *P. praeruptorum*. Comparing the distribution of synonymous substitutions per synonymous site (Ks) in *P. praeruptorum* and the other Apiaceae species shows that the Apiaceae members experienced two WGD events. This is consistent with the previously published data and may be a distinctive genomic signature of the Apiaceae family [11,99,100]. A chromosomal collinear analysis compared *P. praeruptorum, D. carota,* and *A. sinensis* to reconstruct plant chromosome evolution. Several chromosomal rearrangements have occurred to reshape the genome landscape of *P. praeruptorum* and are disclosed here (Figure 2D and Supplementary Figure S16).

The molecular basis of coumarin biosynthesis and their distribution have been described previously [4]. Most coumarins we evaluated here were also detected in the roots, which is consistent with the expression patterns of its biosynthetic genes. However, we also detected some of the coumarins solely accumulated in the aerial parts, which had not been disclosed in the past (Figure 3B).

P450s are a well-established enzyme superfamily present across various organisms. Their primary function involves catalyzing monooxygenation/oxidation reactions, making them valuable tools for constructing intricate molecules [101,102]. These enzymes play essential roles in complex metabolic networks, serving as major contributors to phytochemical

diversification and aiding in adaptation to fluctuating environmental conditions [102,103]. In the late 1980s, Hamerski and Mattern identified that a P450 in *Ammi majus* serve as the marmesin synthase (MS) [104]. In *P. praeruptorum*, two P450s (e.g. PpOC and PpDC) belong to CYP71 or CYP71-clan families are identified [105]. In addition, the physical clustering of homologous P450s, often observed in recently tandem-duplicated P450s, indicates active evolutionary dynamics favoring the acquisition of new activities. It also suggests that some of the clustered genes may function in the same pathway [102,103]. This clustering phenomenon has been previously identified in genes associated with furanocoumarin biosynthesis, as seen with CYP71AJ3 and CYP71AJ4 in *Pastinaca sativa* [106]. With this T2T genome, we recognized *PpPT2* and *PpOC* are clustered as functional gene pairs and two other annotated *PT* and *C2'H* gene pairs on chromosome 11 (Figure 3D). With the elucidation of PpOC that catalyzes the formation of lomatin, the enzyme that is involved in the hydroxylation of C-4′ skeleton to form a khellactone are still elusive. Since several hydroxylases form functional gene pairs with the *PTs* and these genes are co-expressed, this gives a hotspot for mining the candidate enzymes for C-4′ skeleton hydroxylation in this region. However, this hypothesis needs to be further confirmed biochemically in the future.

Data Availability

The genome sequencing data, including PacBio HiFi, ONT Ultra-long, DNBseq short reads, Hi-C data, and transcriptome data, have been deposited into the NCBI database and are available via the BioProject accession number PRJNA1011536. The genome assemblies and gene annotations have been deposited at Figshare [107]. All additional supporting data are available in the *GigaScience* repository, GigaDB [108].

Additional Files

Supplementary Figure S1: The K-mer depth distribution for *Peucedanum praeruptorum* Dunn genome size evaluation.

Supplementary Figure S2: The pipeline for the assembly of the T2T genome of *Peucedanum praeruptorum* Dunn.

Supplementary Figure S3: Statistical graph of correlation analysis between GC content and depth (short and long reads).

Supplementary Figure S4: The Gene structure prediction results and gene set statistics (comparison with gene elements of closely related species).

Supplementary Figure S5: The cumulative distribution statistics graph of gene set element length (comparison with gene elements of closely related species).

Supplementary Figure S6: Venn diagram of homologous gene families of *Peucedanum praeruptorum* Dunn in the genome.

Supplementary Figure S7: GO enrichment annotation of *Peucedanum praeruptorum* Dunn.

Supplementary Figure S8: Kyoto Encyclopedia of Genes and Genomes (KEGG) enrichment annotation of *Peucedanum praeruptorum* Dunn.

Supplementary Figure S9: Venn diagram of functional annotation of *Peucedanum praeruptorum* Dunn in different databases.

Supplementary Figure S10: The syntenic comparison with the newly published genome of *Peucedanum praeruptorum* Dunn.

Supplementary Figure S11: The phylogenetic tree of the 11 species genomes with 489 single-copy genes.

Supplementary Figure S12: GO enrichment results of *Peucedanum praeruptorum* Dunn contraction gene family.

Supplementary Figure S13： KEGG enrichment results of *Peucedanum praeruptorum* Dunn contraction gene family.

Supplementary Figure S14: GO enrichment results of *Peucedanum praeruptorum* Dunn expansion gene family.

Supplementary Figure S15: KEGG enrichment results of *Peucedanum praeruptorum* Dunn expansion gene family.

Supplementary Figure S16: The gene synthetic compaction among the *Angelica sinensis*, *Peucedanum praeruptorum* Dunn, and *Daucus carota*.

Supplementary Figure S17: Inference of polyploidization and speciation history in Apiaceae.

Supplementary Figure S18: Gene family identification and analysis of the terpene biosynthetic pathway.

Supplementary Figure S19: Gene family identification and analysis of the coumarin biosynthetic pathway.

Supplementary Figure S20: The phylogenetic tree of different species genomes with PT genes.

Supplementary Data Table S1: Statistical results of DNBSEQ platform sequencing data for genome survey.

Supplementary Data Table S2: Summary statistics for Pacbio HiFi data, ONT Ultra-long, and Hi-C data.

Supplementary Data Table S3: Statistics of genome assembly result of *Peucedanum praeruptorum* Dunn species.

Supplementary Data Table S4: Statistics of the T2T assembled chromosomes of *Peucedanum praeruptorum* Dunn.

Supplementary Data Table S5: Telomere and Centromere sequence identification.

Supplementary Data Table S6: Completeness estimation of *Peucedanum praeruptorum* Dunn genome with Benchmarking Universal Single-Copy Orthologs (BUSCO) analysis.

Supplementary Data Table S7: The statistical results of the repeat sequence.

Supplementary Data Table S8: Repeat sequence classification result statistics.

Supplementary Data Table S9: Basic statistical results of gene annotation prediction.

Supplementary Data Table S10: Statistics of genes annotated in the *Peucedanum praeruptorum* Dunn genome.

Supplementary Data Table S11: The statistical results of gene function annotation.

Supplementary Data Table S12: Statistics of non-coding RNA annotation results.

Supplementary Data Table S13: The comparison of assembly and annotation with the newly published genome of *Peucedanum praeruptorum* Dunn.

Supplementary Data Table S14: The comparison of chromosomes with the newly published genome of *Peucedanum praeruptorum* Dunn

Supplementary Data Table S15: Summary of gene family clustering.

Supplementary Data Table S16: Gene family identification and the phylogenetic analysis of the terpene biosynthetic pathway.

Supplementary Data Table S17: Expression profiles of TPS gene family in different tissues.

Supplementary Data Table S18: The genes associated with the coumarin metabolic pathway and the expression levels of those genes in various tissues and different growth stages.

Supplementary Data Table S19: The statistical results of the PT gene in the four species were utilized for analysis.

Supplementary Data Table S20: The coumarin compounds data obtained through HPLC-MS analysis in different tissues.

Supplementary Data Table S21: The statistical results of the COSY genes in three species were utilized for analysis.

Supplementary Data Table S22: Phylogenomic analysis of cytochrome P450 multigene family.

Supplementary Data Table S23: The primers used in this study.

List of abbreviations

BLAST: Basic Local Alignment Search Tool; Mb: megabase; Gb: gigabase; GO: Gene Ontology; KEGG: Kyoto Encyclopedia of Genes and Genomes; BUSCO: Benchmarking Universal Single-Copy Orthologs; DUP: duplication; CDS: coding sequence; LINE: long interspersed nucleotide element; Chr: Chromosome; TE: transposable element; LTR: long terminal repeat; HPLC: high-performance liquid chromatography; TD: tandem duplication; WGD: whole genome duplication.

Ethics approval and consent to participate

No ethical approval/permission is required to obtain the materials and perform the research in this study.

Competing interests

The authors declare that they have no competing interests.

Funding

This work was supported by the National Key Research and Development Program of China (Grant No.2022YFD1201600) and Shenzhen-Hong Kong-Macao Science and Technology Innovation Project (Category C) (Ref No: EF038/ICMS-LMY/2021/SZSTIC).

Authors' contributions

MB., SJ., and SC.: designed the research. MB., TYC.: performed analyses in the manuscript and prepared the manuscript. SC., LZ., QL., NL., and ZM.: sampled and conducted the experiments. MB., YY., DS., CL., and WX.: analysed the data. SJ., CZ., JJ., and SZ.: revised the manuscript and editing. HTS.: initiated the writing. SZ., TYC., and HTS.: conceived and supervised the project. All authors read and contributed to the final manuscript.

Figure Legends

Figure 1. Overview of the *P. praeruptorum* and its T2T genome.

A. The morphological characteristics of *P. praeruptorum* in three developmental stages. VP: Vegetative Period; AP: Anthesis Period; FP: Fruit Period. **B.** The circos plot from the outer to the inner circle represents eleven T2T chromosomes (Chr01-Chr11). The distribution of genome features within 3Mb windows is presented: a, GC contents; b, gene density; c, repeats density; d, LTR density; e, LINE density; f, DNA-TE density. **C.** The identifications of telomeres and centromeres of the eleven chromosomes. The orange circles represent telomeres on the assembled chromosomes. The high gene densities are displayed in red, and the low gene densities are displayed in blue. **D**. Hi-C heatmap demonstrated the interactions between eleven chromosomes.

Figure 2. Comparative genomic analysis of the *P. praeruptorum* T2T genome.

A. The estimation of divergence time and gene family expansion/contraction. The numbers next to each branch node represent the estimated divergence time (million years ago, Mya), with the confidence range in brackets. The pie chart demonstrates the ratio of gene families with expansion (green), contraction (red), and stable (blue). **B.** Number of homologous genes shared by different species. **C.** The gene family clustering is demonstrated by the Petal Map.

The middle circle is the number of gene families common to all species, and the edge is the number of gene families unique to each species. **D.** The chromosomal collinearity among *C. sativum*, *P. praeruptorum,* and *D. carota*. **E.** The Ks distribution map within and between species

Figure 3. The coumarins biosynthesis pathway in *P. praeruptorum.*

A. The coumarin biosynthesis candidate genes identification in *P. praeruptorum*. **B.** The distribution of coumarins in five different tissues and three different developmental periods. **C.** The phylogenetic analysis of COSY and collinearity analysis between *A. sinensis* and *P. praeruptorum.* **D.** Chromosomal mapping and gene cluster identification of coumarin biosynthetic genes: prenyltransferases (*PT*s), *PpOC*, and *p*-coumaroyl CoA 2'-hydroxylases (*C'2H*)**. E.** Expression profiling of candidate genes pairs in the coumarin synthesis pathway**.**

Figure 4. Cytochrome P450 genes related to coumarins biosynthesis in *P. praeruptorum*.

A. Motif analysis in cis-elements of the coumarins biosynthesis related cytochrome P450 genes. **B.** The gene structures of the coumarins biosynthesis related cytochrome P450 genes. **C.** The analysis of key cis-elements identified in the promoter regions of coumarin-related cytochrome P450 genes. **D.** Phylogenetic tree of cytochrome P450 genes involved in the coumarin biosynthesis. **E.** The gene location of coumarin-related cytochrome P450 genes on the chromosomes.

Figure 5. A co-expression network connecting structural genes in coumarin biosynthesis with transcription factors (TFs) represents the regulation of coumarin biosynthetic genes. The nodes represent structural genes in coumarin biosynthesis and transcription factors. The node size shows the expression changes of each gene (log2FoldChange (root_fp/root_vp)). The

numbers of the nodes demonstrate the number of TF associated with the core biosynthetic genes.

References

1. Song Y, Jing W, Yan R, Wang Y. Research progress of the studies on the roots of *Peucedanum praeruptorum* dunn (Peucedani radix). *Pak J Pharm Sci*. 2015. 28:71–81

2. Seigler DS. Coumarins. *Plant Secondary Metabolism*. 1998. Springer US; https://doi.org/10.1007/978-1-4615-4913-0_9

3. Author A, Berenbaum MR. Chemical Mediation of Coevolution: Phylogenetic Evidence for. Source: Annals of the Missouri Botanical Garden. 2001. Missouri Botanical Garden Press pp. 45-59. https://doi.org/10.2307/2666131

4. Robe K, Izquierdo E, Vignols F, Rouached H, Dubos C. The Coumarins: Secondary Metabolites Playing a Primary Role in Plant Nutrition and Health. *Trends Plant Sci*. 2021; doi: 10.1016/j.tplants.2020.10.008.

5. Bourgaud F, Hehn A, Larbat R, Doerper S, Gontier E, Kellner S, et al. Biosynthesis of coumarins in plants: A major pathway still to be unravelled for cytochrome P450 enzymes. Phytochem Rev 5, 293–308 (2006). https://doi.org/10.1007/s11101-006-9040-2

6. Rodrigues JL, Rodrigues LR. Biosynthesis and heterologous production of furanocoumarins: perspectives and current challenges. *Nat Prod Rep*. The Royal Society of Chemistry; 2021; doi: 10.1039/D0NP00074D.

7. Río JA Del, Díaz L, García-Bernal D, Blanquer M, Ortuño A, Correal E, et al. Chapter 5 - Furanocoumarins: Biomolecules of Therapeutic Interest. In: Atta-ur-Rahman, editor. Elsevier;

8. Karamat F, Olry A, Munakata R, Koeduka T, Sugiyama A, Paris C, et al. A coumarin-specific prenyltransferase catalyzes the crucial biosynthetic reaction for furanocoumarin formation in parsley. *Plant Journal*. 2014; doi: 10.1111/tpj.12409.

9. Zhao Y, He Y, Han L, Zhang L, Xia Y, Yin F, et al. Two types of coumarins-specific enzymes complete the last missing steps in pyran- and furanocoumarins biosynthesis. *Acta Pharm Sin B*. Chinese Academy of Medical Sciences; 2023; doi: 10.1016/j.apsb.2023.10.016.

10. Chu S, Chen L, Xie H, Xie J, Zhao Y, Tong Z, et al. Comparative analysis and chemical profiling of different forms of Peucedani Radix. *J Pharm Biomed Anal*2020; doi: 10.1016/j.jpba.2020.113410.

11. Wang Y-H, Liu P-Z, Liu H, Zhang R-R, Liang Y, Xu Z-S, et al. Telomere-to-telomere carrot (*Daucus carota*) genome assembly reveals carotenoid characteristics. *Hortic Res*. 2023; doi: 10.1093/hr/uhad103.

12. Song X, Wang J, Li N, Yu J, Meng F, Wei C, et al. Deciphering the high-quality genome sequence of coriander that causes controversial feelings. *Plant Biotechnol J*. 2020; doi: 10.1111/pbi.13310.

13. Song X, Sun P, Yuan J, Gong K, Li N, Meng F, et al. The celery genome sequence reveals sequential paleo-polyploidizations, karyotype evolution and resistance gene reduction in apiales. *Plant Biotechnol J*. 2021; doi: 10.1111/pbi.13499.

14. Li MY, Feng K, Hou XL, Jiang Q, Xu ZS, Wang GL, et al. The genome sequence of celery (Apium graveolens L.), an important leaf vegetable crop rich in apigenin in the Apiaceae family. *Hortic Res*. 2020; doi: 10.1038/s41438-019-0235-2.

15. Li S, Chiu TY, Jin X, Cao D, Xu M, Zhu M, et al. Integrating genomic and multiomic data for Angelica sinensis provides insights into the evolution and biosynthesis of pharmaceutically bioactive compounds. *Commun Biol*. 2023; doi: 10.1038/s42003-023-05569-5.

16. Han X, Li C, Sun S, Ji J, Nie B, Maker G, et al. The chromosome-level genome of female ginseng (*Angelica sinensis*) provides insights into molecular mechanisms and evolution of coumarin biosynthesis. *Plant Journal* 2022; doi: 10.1111/tpj.16007.

17. Zhang Q, Li M, Chen X, Liu G, Zhang Z, Tan Q, et al. Chromosome-Level Genome Assembly of Bupleurum chinense DC Provides Insights Into the Saikosaponin Biosynthesis. *Front Genet*. Frontiers Media S.A.; 2022; doi: 10.3389/fgene.2022.878431.

18. Song C, Zhang Y, Manzoor MA, Wei P, Yi S, Chu S, et al. A chromosome-scale genome of *Peucedanum praeruptorum* provide insights into Apioideae evolution and medicinal ingredient biosynthesis. *Int J Biol Macromol*. Elsevier B.V.; 2024; doi: 10.1016/j.ijbiomac.2023.128218.

19. Chin C-S, Alexander DH, Marks P, Klammer AA, Drake J, Heiner C, et al. Nonhybrid, finished microbial genome assemblies from long-read SMRT sequencing data. *Nat Methods*. 2013; doi: 10.1038/nmeth.2474.

20. Durand NC, Robinson JT, Shamim MS, Machol I, Mesirov JP, Lander ES, et al. Juicebox Provides a Visualization System for Hi-C Contact Maps with Unlimited Zoom. *Cell Syst*. 2016; doi: 10.1016/j.cels.2015.07.012.

21. Marçais G, Kingsford C. A fast, lock-free approach for efficient parallel counting of occurrences of k-mers. *Bioinformatics*. 2011; doi: 10.1093/bioinformatics/btr011.

22. Vurture GW, Sedlazeck FJ, Nattestad M, Underwood CJ, Fang H, Gurtowski J, et al. GenomeScope: fast reference-free genome profiling from short reads. *Bioinformatics*. 2017; doi: 10.1093/bioinformatics/btx153.

23. Cheng H, Concepcion GT, Feng X, Zhang H, Li H. Haplotype-resolved de novo assembly using phased assembly graphs with hifiasm. *Nat Methods*. 2021; doi: 10.1038/s41592-020-01056-5.

24. Dudchenko O, Batra SS, Omer AD, Nyquist SK, Hoeger M, Durand NC, et al. De novo assembly of the Aedes aegypti genome using Hi-C yields chromosome-length scaffolds. *Science*. 2017; doi: 10.1126/science.aal3327.

25. Roach MJ, Schmidt SA, Borneman AR. Purge Haplotigs: Allelic contig reassignment for third-gen diploid genome assemblies. *BMC Bioinformatics*. 2018; doi: 10.1186/s12859-018-2485-7.

26. Robinson JT, Turner D, Durand NC, Thorvaldsdóttir H, Mesirov JP, Aiden EL. Juicebox.js Provides a Cloud-Based Visualization System for Hi-C Data. *Cell Syst*. 2018; doi: 10.1016/j.cels.2018.01.001.

27. Li H. Minimap2: pairwise alignment for nucleotide sequences. *Bioinformatics*. 2018; doi: 10.1093/bioinformatics/bty191.

28. Xu M, Guo L, Gu S, Wang O, Zhang R, Peters BA, et al. TGS-GapCloser: A fast and accurate gap closer for large genomes with low coverage of error-prone long reads. *Gigascience* 2020; doi: 10.1093/gigascience/giaa094.

29. Wang Y, Zhao Y, Bollas A, Wang Y, Au KF. Nanopore sequencing technology, bioinformatics and applications. Nat Biotechnol. 2021 Nov;39(11):1348-1365. doi: 10.1038/s41587-021-01108-x.

30. Camacho C, Coulouris G, Avagyan V, Ma N, Papadopoulos J, Bealer K, et al. BLAST+: Architecture and applications. *BMC Bioinformatics*. 2009; doi: 10.1186/1471-2105-10-421.

31. Walker BJ, Abeel T, Shea T, Priest M, Abouelliel A, Sakthikumar S, et al. Pilon: an integrated tool for comprehensive microbial variant detection and genome assembly improvement. *PLoS One*. 2014; doi: 10.1371/journal.pone.0112963.

32. Manni M, Berkeley MR, Seppey M, Simão FA, Zdobnov EM. BUSCO Update: Novel and Streamlined Workflows along with Broader and Deeper Phylogenetic Coverage for Scoring of Eukaryotic, Prokaryotic, and Viral Genomes. *Mol Biol Evol*. 2021; doi: 10.1093/molbev/msab199.

33. Benson G. Tandem repeats finder: a program to analyze DNA sequences. *Nucleic Acids Res*. 1999; doi: 10.1093/nar/27.2.573.

34. Saha S, Bridges S, Magbanua Z V., Peterson DG. Empirical comparison of ab initio repeat finding programs. *Nucleic Acids Res*. 2008; doi: 10.1093/nar/gkn064.

35. Bao W, Kojima KK, Kohany O. Repbase Update, a database of repetitive elements in eukaryotic genomes. *Mob DNA*. 2015; doi: 10.1186/s13100-015-0041-9.

36. Flynn JM, Hubley R, Goubert C, Rosen J, Clark AG, Feschotte C, et al. RepeatModeler2 for automated genomic discovery of transposable element families. *Proceedings of the National Academy of Sciences*. 2020; doi: 10.1073/pnas.1921046117.

37. Xu Z, Wang H. LTR_FINDER: an efficient tool for the prediction of full-length LTR retrotransposons. *Nucleic Acids Res*. 2007; doi: 10.1093/nar/gkm286.

38. Slater G, Birney E. Automated generation of heuristics for biological sequence comparison. *BMC Bioinformatics*. 2005; doi: 10.1186/1471-2105-6-31.

39. Shumate A, Salzberg SL. Liftoff: accurate mapping of gene annotations. *Bioinformatics*. 2021; doi: 10.1093/bioinformatics/btaa1016.

40. Stanke M, Schöffmann O, Morgenstern B, Waack S. Gene prediction in eukaryotes with a generalized hidden Markov model that uses hints from external sources. *BMC Bioinformatics*. 2006; doi: 10.1186/1471-2105-7-62.

41. Korf I. Gene finding in novel genomes. *BMC Bioinformatics*. 2004; doi: 10.1186/1471-2105-5-59.

42. Kim D, Langmead B, Salzberg SL. HISAT: a fast spliced aligner with low memory requirements. *Nat Methods*. 2015; doi: 10.1038/nmeth.3317.

43. Kovaka S, Zimin A V., Pertea GM, Razaghi R, Salzberg SL, Pertea M. Transcriptome assembly from long-read RNA-seq alignments with StringTie2. *Genome Biol*. 2019; doi: 10.1186/s13059-019-1910-1.

44. Holt C, Yandell M. MAKER2: an annotation pipeline and genome-database management tool for second-generation genome projects. *BMC Bioinformatics*. 2011; doi: 10.1186/1471-2105-12-491.

45. Chan PP, Lin BY, Mak AJ, Lowe TM. TRNAscan-SE 2.0: Improved detection and functional classification of transfer RNA genes. *Nucleic Acids Res*. 2021; doi: 10.1093/nar/gkab688.

46. Kalvari I, Nawrocki EP, Ontiveros-Palacios N, Argasinska J, Lamkiewicz K, Marz M, et al. Rfam 14: Expanded coverage of metagenomic, viral and microRNA families. *Nucleic Acids Res*. 2021; doi: 10.1093/nar/gkaa1047.

47. Li L, Stoeckert CJ, Roos DS. OrthoMCL: Identification of Ortholog Groups for Eukaryotic Genomes. *Genome Res*. 2003; doi: 10.1101/gr.1224503.

48. Katoh K, Standley DM. MAFFT Multiple Sequence Alignment Software Version 7: Improvements in Performance and Usability. *Mol Biol Evol*. 2013; doi: 10.1093/molbev/mst010.

49. Castresana J. Selection of Conserved Blocks from Multiple Alignments for Their Use in Phylogenetic Analysis. *Mol Biol Evol*. 2000; doi: 10.1093/oxfordjournals.molbev.a026334.

50. Stamatakis A. RAxML version 8: a tool for phylogenetic analysis and post-analysis of large phylogenies. *Bioinformatics*. 2014; doi: 10.1093/bioinformatics/btu033.

51. Yang Z. PAML 4: Phylogenetic Analysis by Maximum Likelihood. *Mol Biol Evol*. 2007; doi: 10.1093/molbev/msm088.

52. De Bie T, Cristianini N, Demuth JP, Hahn MW. CAFE: a computational tool for the study of gene family evolution. *Bioinformatics*. 2006; doi: 10.1093/bioinformatics/btl097.

53. Sun P, Jiao B, Yang Y, Shan L, Li T, Li X, et al. WGDI: A user-friendly toolkit for evolutionary analyses of whole-genome duplications and ancestral karyotypes. *Mol Plant*. 2022; doi: 10.1016/j.molp.2022.10.018.

54. Chen Y, Chen Y, Shi C, Huang Z, Zhang Y, Li S, et al. SOAPnuke: a MapReduce acceleration-supported software for integrated quality control and preprocessing of high-throughput sequencing data. *Gigascience*. 2018; doi: 10.1093/gigascience/gix120.

55. Langmead B, Salzberg SL. Fast gapped-read alignment with Bowtie 2. *Nat Methods*. 2012; doi: 10.1038/nmeth.1923.

56. Li B, Dewey CN. RSEM: accurate transcript quantification from RNA-Seq data with or without a reference genome. *BMC Bioinformatics*. 2011; doi: 10.1186/1471-2105-12-323.

57. Love MI, Huber W, Anders S. Moderated estimation of fold change and dispersion for RNA-seq data with DESeq2. *Genome Biol*. 2014; doi: 10.1186/s13059-014-0550-8.

58. Langfelder P, Horvath S. WGCNA: an R package for weighted correlation network analysis. *BMC Bioinformatics*. 2008; doi: 10.1186/1471-2105-9-559.

59. Shannon P, Markiel A, Ozier O, Baliga NS, Wang JT, Ramage D, et al. Cytoscape: A software Environment for integrated models of biomolecular interaction networks. *Genome Res*. 2003; doi: 10.1101/gr.1239303.

60. Livak KJ, Schmittgen TD. Analysis of relative gene expression data using real-time quantitative PCR and the 2-ΔΔCT method. *Methods*. 2001; doi: 10.1006/meth.2001.1262.

61. Tohge T, Fernie AR. Combining genetic diversity, informatics and metabolomics to facilitate annotation of plant gene function. *Nat Protoc*. 2010; doi: 10.1038/nprot.2010.82.

62. Chen C, Chen H, Zhang Y, Thomas HR, Frank MH, He Y, et al. TBtools: An Integrative Toolkit Developed for Interactive Analyses of Big Biological Data. *Mol Plant*. 2020; doi: 10.1016/j.molp.2020.06.009.

63. Bailey TL, Boden M, Buske FA, Frith M, Grant CE, Clementi L, et al. MEME SUITE: tools for motif discovery and searching. *Nucleic Acids Res*. 2009; doi: 10.1093/nar/gkp335.

64. Lescot M. PlantCARE, a database of plant cis-acting regulatory elements and a portal to tools for in silico analysis of promoter sequences. *Nucleic Acids Res*. 2002; doi: 10.1093/nar/30.1.325.

65. Jung Y, Han D. BWA-MEME: BWA-MEM emulated with a machine learning approach. *Bioinformatics*. 2022; doi: 10.1093/bioinformatics/btac137.

66. Holt C, Yandell M. MAKER2: an annotation pipeline and genome-database management tool for second-generation genome projects. *BMC Bioinformatics*. 2011; doi: 10.1186/1471-2105-12-491.

67. Rasmussen SK, Avato P. Characterization of Chromosomes and Genome Organization of Thapsia Garganica L. by Localizations of rRNA Genes using Fluorescent in Situ Hybridization. *Hereditas*. John Wiley & Sons, Ltd; 1998; doi: https://doi.org/10.1111/j.1601-5223.1998.t01-1-00231.x.

68. Weitzel C, Rønsted N, Spalik K, Simonsen HT. Resurrecting deadly carrots: Towards a revision of *Thapsia* (Apiaceae) based on phylogenetic analysis of nrITS sequences and chemical profiles. *Botanical Journal of the Linnean Society*. 2014; doi: 10.1111/boj.12144.

69. Vogt T. Phenylpropanoid Biosynthesis. *Mol Plant*. 2010; doi: 10.1093/mp/ssp106.

70. Karamat F, Olry A, Doerper S, Vialart G, Ullmann P, Werck-Reichhart D, et al. CYP98A22, a phenolic ester 3'-hydroxylase specialized in the synthesis of chlorogenic acid, as a new tool for enhancing the furanocoumarin concentration in Ruta graveolens. *BMC Plant Biol*. 2012; doi: 10.1186/1471-2229-12-152.

71. Kai K, Mizutani M, Kawamura N, Yamamoto R, Tamai M, Yamaguchi H, et al. Scopoletin is biosynthesized via *ortho* -hydroxylation of feruloyl CoA by a 2-oxoglutarate-dependent dioxygenase in *Arabidopsis thaliana*. *The Plant Journal*. 2008; doi: 10.1111/j.1365-313X.2008.03568.x.

72. Vialart G, Hehn A, Olry A, Ito K, Krieger C, Larbat R, et al. A 2-oxoglutarate-dependent dioxygenase from Ruta graveolens L. exhibits p-coumaroyl CoA 2′-hydroxylase activity (C2′H): a missing step in the synthesis of umbelliferone in plants. *The Plant Journal*. 2012; doi: 10.1111/j.1365-313X.2011.04879.x.

73. Matsumoto S, Mizutani M, Sakata K, Shimizu B-I. Molecular cloning and functional analysis of the ortho-hydroxylases of p-coumaroyl coenzyme A/feruloyl coenzyme A involved in formation of umbelliferone and scopoletin in sweet potato, Ipomoea batatas (L.) Lam. *Phytochemistry*. 2012; doi: 10.1016/j.phytochem.2011.11.009.

74. Vanholme R, Sundin L, Seetso KC, Kim H, Liu X, Li J, et al. COSY catalyses trans–cis isomerization and lactonization in the biosynthesis of coumarins. *Nat Plants*. Palgrave Macmillan Ltd.; 2019; doi: 10.1038/s41477-019-0510-0.

75. Karamat F, Olry A, Munakata R, Koeduka T, Sugiyama A, Paris C, et al. A coumarin-specific prenyltransferase catalyzes the crucial biosynthetic reaction for furanocoumarin formation in parsley. *Plant Journal*. 2014; doi: 10.1111/tpj.12409.

76. Mao L, Kawaide H, Higuchi T, Chen M, Miyamoto K, Hirata Y, et al. Genomic evidence for convergent evolution of gene clusters for momilactone biosynthesis in land plants. *Proc Natl Acad Sci U S A*. National Academy of Sciences; 2020; doi: 10.1073/pnas.1914373117.

77. Wu YS, Hillwig ML, Wang Q, Peters RJ. Parsing a multifunctional biosynthetic gene cluster from rice: Biochemical characterization of CYP71Z6 & 7. *FEBS Lett*. 2011; doi: 10.1016/j.febslet.2011.09.038.

78. Wang Q, Hillwig ML, Okada K, Yamazaki K, Wu Y, Swaminathan S, et al. Characterization of CYP76M5–8 Indicates Metabolic Plasticity within a Plant Biosynthetic Gene Cluster. *Journal of Biological Chemistry*. 2012; doi: 10.1074/jbc.M111.305599.

79. Bryson AE, Lanier ER, Lau KH, Hamilton JP, Vaillancourt B, Mathieu D, et al. Uncovering a miltiradiene biosynthetic gene cluster in the Lamiaceae reveals a dynamic evolutionary trajectory. *Nat Commun*. 2023; doi: 10.1038/s41467-023-35845-1.

80. Takos AM, Knudsen C, Lai D, Kannangara R, Mikkelsen L, Motawia MS, et al. Genomic clustering of cyanogenic glucoside biosynthetic genes aids their identification in *Lotus japonicus* and suggests the repeated evolution of this chemical defence pathway. *The Plant Journal*. 2011; doi: 10.1111/j.1365-313X.2011.04685.x.

81. Soler-Oliva ME, Guerrero-Martínez JA, Bachetti V, Reyes JC. Analysis of the relationship between coexpression domains and chromatin 3D organization. *PLoS Comput Biol*. Public Library of Science; 2017; doi: 10.1371/journal.pcbi.1005708.

82. Nelson D, Werck-Reichhart D. A P450-centric view of plant evolution. *Plant Journal*. 2011; doi: 10.1111/j.1365-313X.2011.04529.x.

83. Weitzel C, Simonsen HT. Cytochrome P450-enzymes involved in the biosynthesis of mono- and sesquiterpenes. *Phytochemistry Reviews*. 2015; doi: 10.1007/s11101-013-9280-x.

84. Hamberger B, Bak S. Plant P450s as versatile drivers for evolution of species-specific chemical diversity. *Philosophical Transactions of the Royal Society B: Biological Sciences*. 2013; doi: 10.1098/rstb.2012.0426.

85. Hamerski D, Schmitt D, Matern U. Induction of two prenyltransferases for the accumulation of coumarin phytoalexins in elicitor-treated Ammi majus cell suspension cultures. *Phytochemistry*. 1990; doi: 10.1016/0031-9422(90)85417-E.

86. Li M-Y, Feng K, Hou X-L, Jiang Q, Xu Z-S, Wang G-L, et al. The genome sequence of celery (Apium graveolens L.), an important leaf vegetable crop rich in apigenin in the Apiaceae family. *Hortic Res*. 2020; doi: 10.1038/s41438-019-0235-2.

87. Vialart G, Hehn A, Olry A, Ito K, Krieger C, Larbat R, et al. A 2-oxoglutarate-dependent dioxygenase from *Ruta graveolens* L. exhibits *p*- coumaroyl CoA 2′-hydroxylase activity

(C2′H): a missing step in the synthesis of umbelliferone in plants. *The Plant Journal*. 2012; doi: 10.1111/j.1365-313X.2011.04879.x.

88. Matsumoto S, Mizutani M, Sakata K, Shimizu B-I. Molecular cloning and functional analysis of the ortho-hydroxylases of p-coumaroyl coenzyme A/feruloyl coenzyme A involved in formation of umbelliferone and scopoletin in sweet potato, Ipomoea batatas (L.) Lam. *Phytochemistry*. 2012; doi: 10.1016/j.phytochem.2011.11.009.

89. Krieger C, Kamo T, Bourgaud F, Olry A, Weitzel C, Dueholm B, et al. Evolution of substrate recognition sites (SRSs) in cytochromes P450 from Apiaceae exemplified by the CYP71AJ subfamily. *BMC Evol Biol*. 2015; doi: 10.1186/s12862-015-0396-z.

90. Xu W, Dubos C, Lepiniec L. Transcriptional control of flavonoid biosynthesis by MYB–bHLH–WDR complexes. *Trends Plant Sci*. 2015; doi: 10.1016/j.tplants.2014.12.001.

91. Sun B, Zhu Z, Cao P, Chen H, Chen C, Zhou X, et al. Purple foliage coloration in tea (Camellia sinensis L.) arises from activation of the R2R3-MYB transcription factor CsAN1. *Sci Rep*. 2016; doi: 10.1038/srep32534.

92. Döll S, Kuhlmann M, Rutten T, Mette MF, Scharfenberg S, Petridis A, et al. Accumulation of the coumarin scopolin under abiotic stress conditions is mediated by the *Arabidopsis thaliana* THO/TREX complex. *The Plant Journal*. 2018; doi: 10.1111/tpj.13797.

93. Simonsen HT, Weitzel C, Christensen SB. Guaianolide sesquiterpenoids: Pharmacology and biosynthesis. In: Ramawat KG, Merillon JM, editors. *Natural Products: Phytochemistry, Botany and Metabolism of Alkaloids, Phenolics and Terpenes*. 2013. Berlin, Germany: Springer-Verlag; ISBN-10: 9783642221439

94. Christensen SB, Simonsen HT, Engedal N, Nissen P, Møller JV, Denmeade SR, et al. From Plant to Patient: Thapsigargin, a Tool for Understanding Natural Product Chemistry, Total Syntheses, Biosynthesis, Taxonomy, ATPases, Cell Death, and Drug Development. In: Kinghorn AD, Falk H, Gibbons S, Asakawa Y, Liu J-K, Dirsch VM, editors. *Progress in the*

Chemistry of Organic Natural Products 115. 2021. Cham: Springer International Publishing; https://doi.org/10.1007/978-3-030-64853-4

95. Drew DP, Krichau N, Reichwald K, Simonsen HT. Guaianolides in Apiaceae: Perspectives on pharmacology and biosynthesis. *Phytochemistry Reviews*. 2009; doi: 10.1007/s11101-009-9130-z.

96. Chen F, Tholl D, Bohlmann J, Pichersky E. The family of terpene synthases in plants: A mid-size family of genes for specialized metabolism that is highly diversified throughout the kingdom. *Plant Journal*. 2011; doi: 10.1111/j.1365-313X.2011.04520.x.

97. Song C, Li X, Jia B, Liu L, Wei P, Manzoor MA, et al. Comparative Transcriptomics Unveil the Crucial Genes Involved in Coumarin Biosynthesis in *Peucedanum praeruptorum* Dunn. *Front Plant Sci*. 2022; doi: 10.3389/fpls.2022.899819.

98. Liao B, Shen X, Xiang L, Guo S, Chen S, Meng Y, et al. Allele-aware chromosome-level genome assembly of Artemisia annua reveals the correlation between ADS expansion and artemisinin yield. *Mol Plant*. 2022; doi: 10.1016/j.molp.2022.05.013.

99. Liu JX, Liu H, Tao JP, Tan GF, Dai Y, Yang LL, et al. High-quality genome sequence reveals a young polyploidization and provides insights into cellulose and lignin biosynthesis in water dropwort (*Oenanthe sinensis*). *Ind Crops Prod*. 2023; doi: 10.1016/j.indcrop.2022.116203.

100. Han X, Li C, Sun S, Ji J, Nie B, Maker G, et al. The chromosome-level genome of female ginseng (Angelica sinensis) provides insights into molecular mechanisms and evolution of coumarin biosynthesis. *Plant Journal*2022; doi: 10.1111/tpj.16007.

101. Nelson DR. Cytochrome P450 diversity in the tree of life. *Biochim Biophys Acta Proteins Proteom*. 2018; doi: 10.1016/j.bbapap.2017.05.003.

102. Nelson D, Werck-Reichhart D. A P450-centric view of plant evolution. *Plant Journal*. 2011; doi: 10.1111/j.1365-313X.2011.04529.x.

103. Mizutani M, Ohta D. Diversification of P450 genes during land plant evolution. *Annu Rev Plant Biol*. 2010; doi: 10.1146/annurev-arplant-042809-112305.

104. Hamerski D, Matern U. Elicitor-induced biosynthesis of psoralens in *Ammi majus* L. suspension cultures. *Eur J Biochem*. 1988; doi: 10.1111/j.1432-1033.1988.tb13800.x.

105. Jian X, Zhao Y, Wang Z, Li S, Li L, Luo J, et al. Two CYP71AJ enzymes function as psoralen synthase and angelicin synthase in the biosynthesis of furanocoumarins in *Peucedanum praeruptorum* Dunn. *Plant Mol Biol*. 2020; doi: 10.1007/s11103-020-01045-4.

106. Roselli S, Olry A, Vautrin S, Coriton O, Ritchie D, Galati G, et al. A bacterial artificial chromosome (BAC) genomic approach reveals partial clustering of the furanocoumarin pathway genes in parsnip. *Plant Journal*. 2017; doi: 10.1111/tpj.13450.

107. Bai M. Genome and Gene of Peucedanum praeruptorum. Figshare Dataset. 2024. https://doi.org/10.6084/m9.figshare.25249453.v1

108. Bai M, Jiang S, Chu S, Yu Y, Shan D, Liu C, et al. Supporting data for "The telomere-to-telomere (T2T) genome of *Peucedanum praeruptorum* Dunn provides insights into the genome evolution and coumarin biosynthesis" GigaScience Database. 2024. https://doi.org/10.5524/102520

Figure 1

Click here to access/download;Figure;Figure 1.pdf

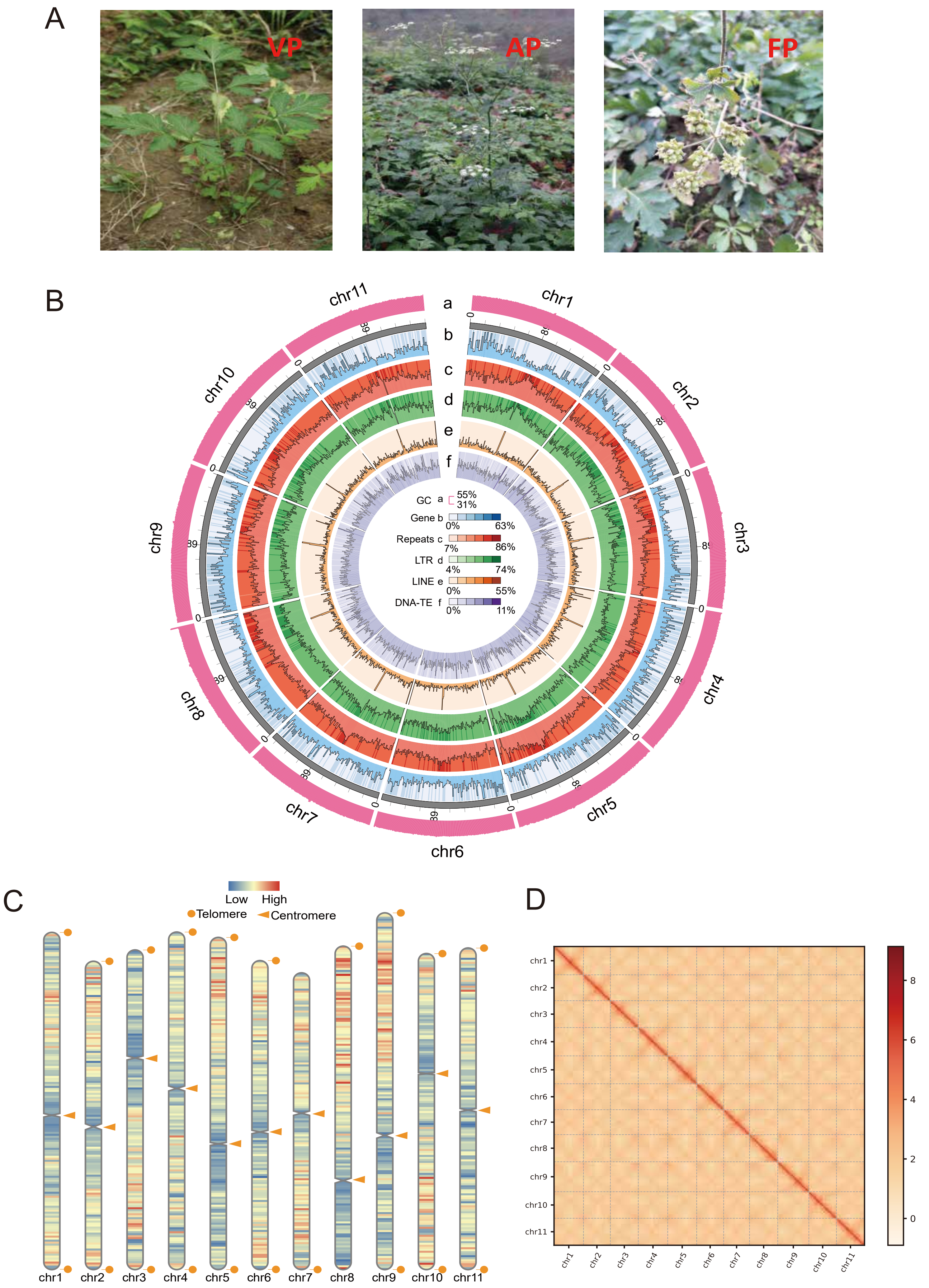

Figure 2

Click here to access/download;Figure;Figure 2-new.pdf

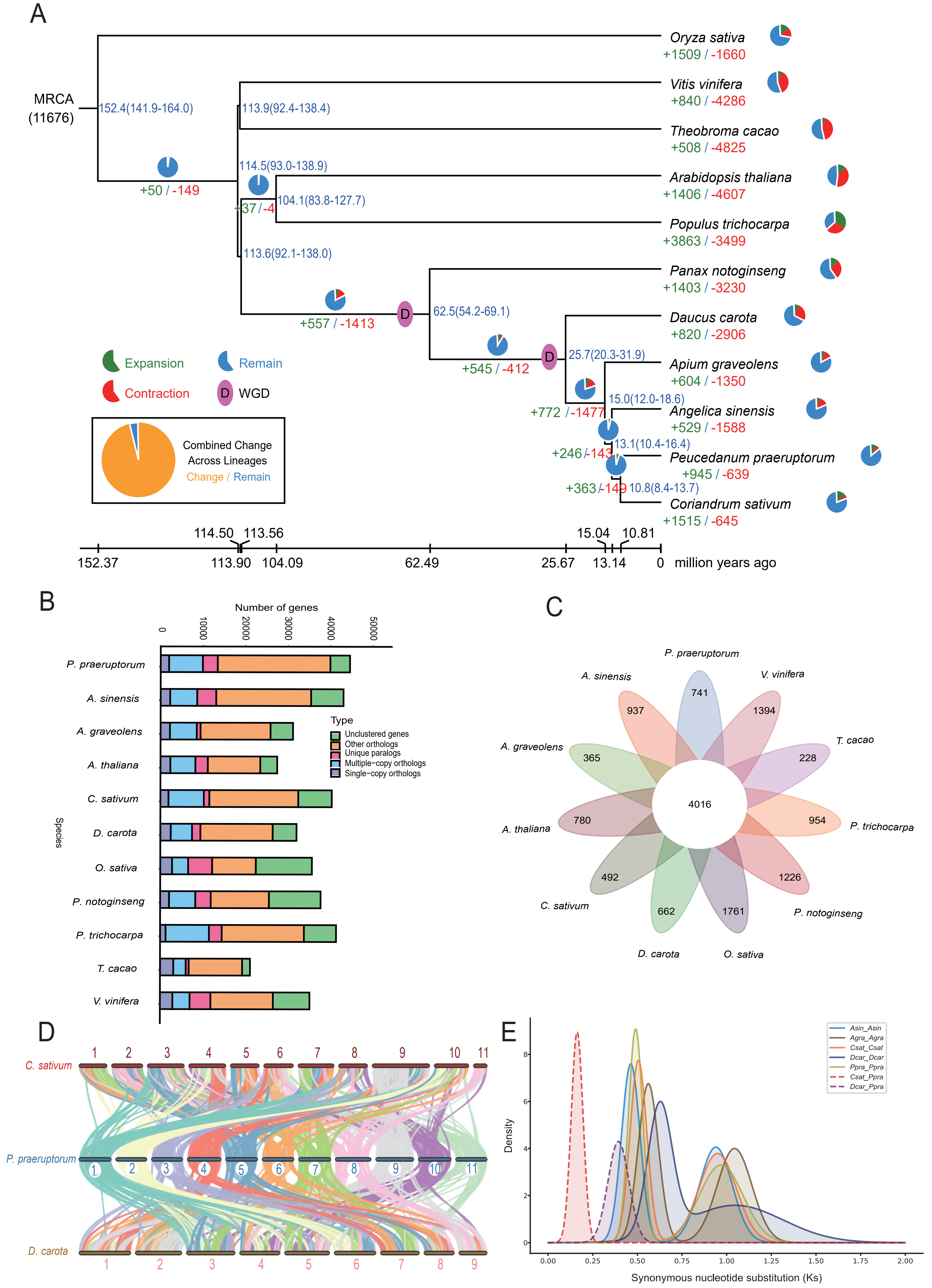

Figure 3 Click here to access/download;Figure;Figure 3.pdf

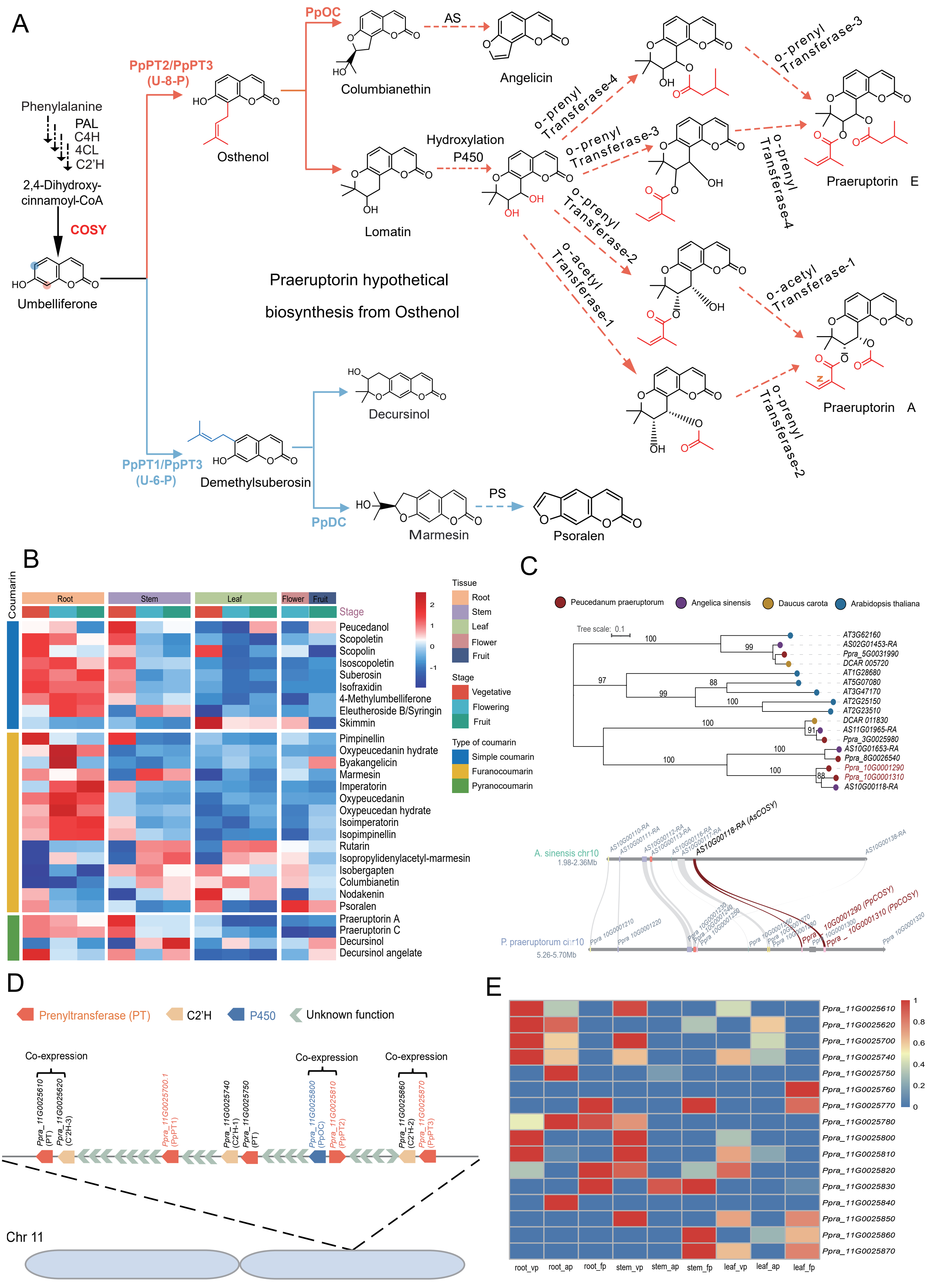

Figure 4

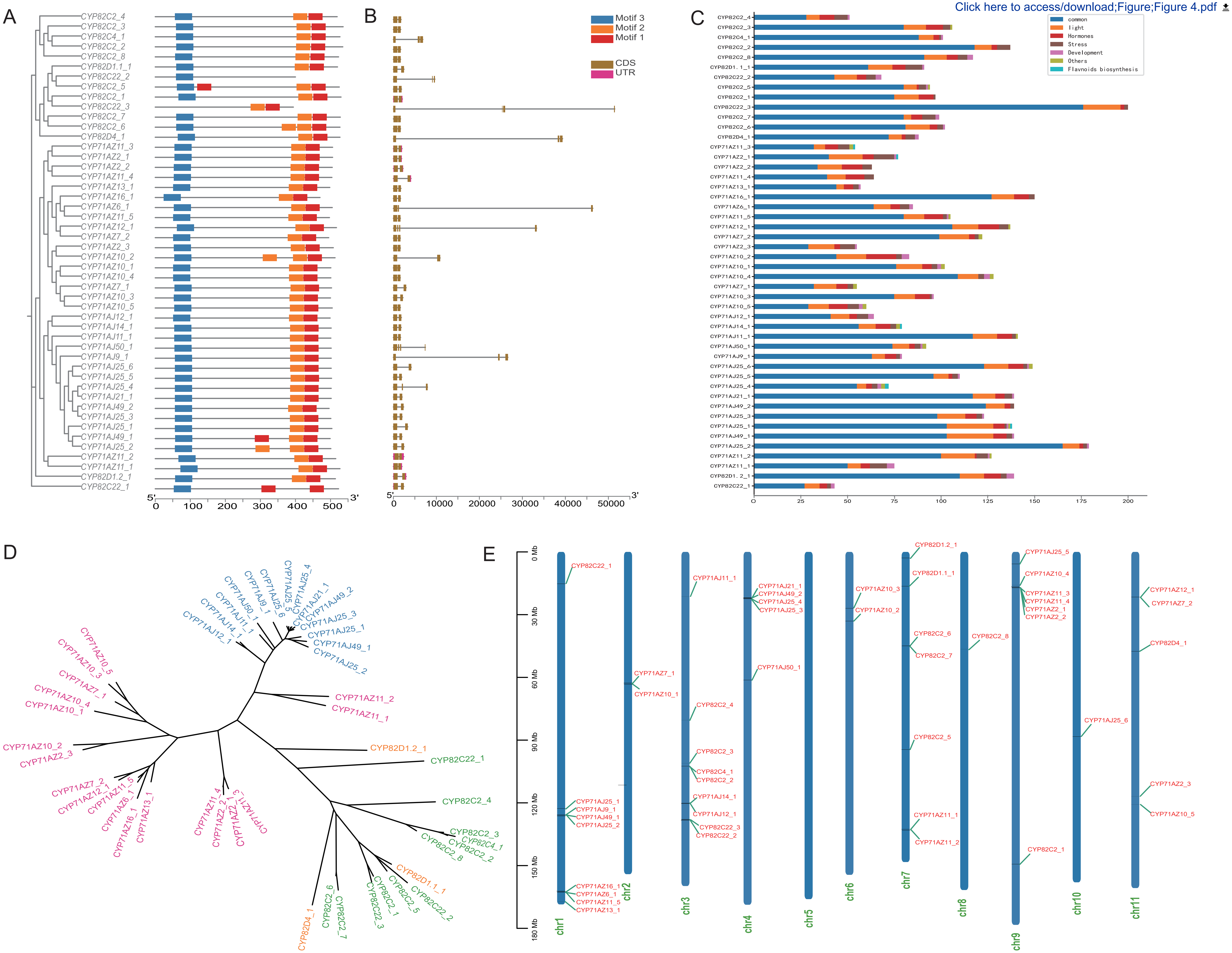

A
CYP82C2_4
CYP82C2_3
CYP82C4_1
CYP82C2_2
CYP82C2_8
CYP82D1.1_1
CYP82C22_2
CYP82C2_5
CYP82C2_1
CYP82C22_3
CYP82C2_7
CYP82C2_6
CYP82D4_1
CYP71AZ11_3
CYP71AZ2_1
CYP71AZ2_2
CYP71AZ11_4
CYP71AZ13_1
CYP71AZ16_1
CYP71AZ6_1
CYP71AZ11_5
CYP71AZ12_1
CYP71AZ7_2
CYP71AZ2_3
CYP71AZ10_2
CYP71AZ10_1
CYP71AZ10_4
CYP71AZ7_1
CYP71AZ10_3
CYP71AZ10_5
CYP71AJ12_1
CYP71AJ14_1
CYP71AJ11_1
CYP71AJ50_1
CYP71AJ9_1
CYP71AJ25_6
CYP71AJ25_5
CYP71AJ25_4
CYP71AJ21_1
CYP71AJ49_2
CYP71AJ25_3
CYP71AJ25_1
CYP71AJ49_1
CYP71AJ25_2
CYP71AZ11_2
CYP71AZ11_1
CYP82D1.2_1
CYP82C22_1
5' 0 100 200 300 400 500 3'
B
Motif 3
Motif 2
Motif 1
CDS
UTR
5' 0 10000 20000 30000 40000 50000 3'
C
common
light
Hormones
Stress
Development
Others
Flavnoids biosynthesis
0 25 50 75 100 125 150 175 200
D
E
0 Mb
30 Mb
60 Mb
90 Mb
120 Mb
150 Mb
180 Mb
chr1
chr2
chr3
chr4
chr5
chr6
chr7
chr8
chr9
chr10
chr11

Figure 5

Click here to access/download;Figure;Figure 5.pdf

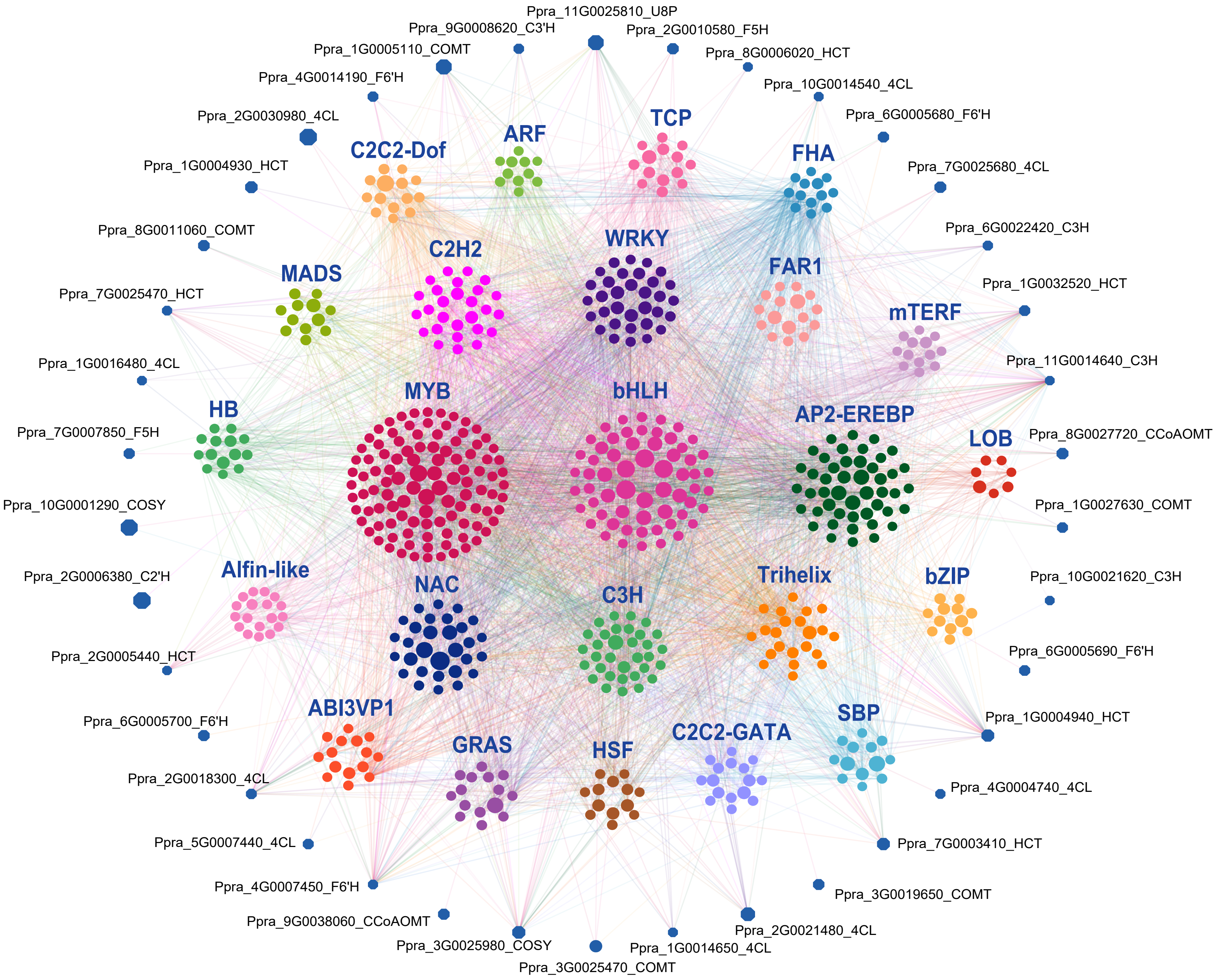

Supp figures

Click here to access/download

Supplementary Material

Supplement Figures Peucedanum praeruptorum Dunn T2T-0217.docx

Click here to access/download
Supplementary Material
Supplyment Tables peucedanum praeruptorum Dunn data0220.xlsx
