# Supplementary material for: The telomere-to-telomere (T2T) genome of Peucedanum praeruptorum Dunn provides insights into the genome evolution and coumarin biosynthesis
Source: Gigascience. 2024 Jun 5;13:giae025. doi: 10.1093/gigascience/giae025 (PMC11152176; doi:10.1093/gigascience/giae025)
Supplement: giae025_Response_to_Reviewer_Comments_Original_Submission [file giae025_response_to_reviewer_comments_original_submission.pdf]

Author's Response To Reviewer Comments

Reviewer reports:
Thanks for the comments. The authors appreciate all the valuable comments from the reviewers. All authors have contributed
manuscript was in review. The corresponding responses were listed below point-by-point.

Reviewer #1: This manuscript completed the assembly and annotation work of the T2T level genome of Peucedanum praerup
provides molecular insights into the chromosomal distribution of the coumarin biosynthetic genes and serves as an important
supplement the validation work of some structural genes or transcription factors. It is also acceptable to add some bioinforma

Response:
Thanks for the comments.
In the revised version, we have modified the manuscript with more analysis and have focused our main story on the importar
Fiugre 3. We identified that all the PpPT1-3 were located on chromosome 11 and noticed that PpPT2 and PpOC formed a func
steps to the Praeruptorin A or Praeruptorin E (Figure 3A). We also analyzed the Terpene synthases (TPS) gene family, which

Some software does not indicate version, please verify the entire manuscript.
Response:
The versions of the applicable software were double checked throughout the entire manuscript. The missed details were adde

Why does the phylogenetic analysis of the P450 family only focus on the CYP71 and CYP82? Can the phylogenetic analysis of
Response:
To identify the functional genes associated with the isopentene group cyclization mechanism, we conducted a comprehensive
derived from Mentha piperita (reference 1). Furthermore, certain enzymes involved in downstream coumarin processes, such
2). Thus, we systematically analyzed the CYP71 family in P. praeruptorum and their cis-elements based on the well-assemble
Reference:
Bertea CM, Schalk M, Karp F, Maffei M, Croteau R. Demonstration that menthofuran synthase of mint (Mentha) is a cytochron
Zhao, Yucheng et al. "Two types of coumarins-specific enzymes complete the last missing steps in pyran- and furanocoumari

Please provide the accession number for genome assembly and annotation
Response:
The genome assemblies and gene annotations have been deposited at Figshare with the accession link： https://figshare.com

The Genome assembly does not describe how the HiFi+Hi-C+Ultra long (365 contig) version of the genome was optimized to
Response:
More detailed information on the process of genome assembly have been supplied in the result section. There were three vers
it reads,
Further, we applied PacBio HiFi sequencing (72.6 Gb), ONT Ultra-Long DNA sequencing (349.7 Gb), and Hi-C sequencing (53
S3, Supplementary Data Table S3). A chromosome-level genome assembly (hereafter named 'chromosome genome') with Hi
chromosomes (Figure 1B and 1C; Supplementary Data Table S3 & S4). This nearly gapless genome consists of 253 contigs, N

Adding non-coding RNA annotation method.
Response:
Thanks for the comments. The non-coding RNA annotation method was added. Now it reads,
For non-coding RNA, tRNAscan-SE (version 1.3.1) was used to identify tRNA sequences in the genome based on the structura
covariance model of the Rfam family and INFERNAL that comes with Rfam (version 14.8).

Adding the Telomere sequence identification method.
Response:
Thanks for the comments. The methods for identification of telomeres and centromeres were added, now it reads,
The ONT ultra-long reads were aligned to the 'chromosome genome' sequences using minimap2 (version 2-2.24) and facilitat
with the median extendable length were defined as ref and the others as query. Medaka_consensu (version 1.7.2, parameter
2.11.0+) according to the positional relationship in the alignment. The telomere sequence was replaced with the aligned sequ
and the LINE/L1 distribution is consistent with the centromere distribution of P. praeruptorum, and this region is also located

Table s3, Please provide statistical information on the HiFi and HiFi+HiC versions of the genome.
Response:
The detailed information on statistics of genome assembly result of P. praeruptorum was supplied. There were three versions
Statistics without reference Draft genome Chromosome genome T2T genome

Table s5, Unexplained why the Telomere sequence was not detected at chr7 and chr8.
Response:
Thanks for the comments. The authors checked that there were repeat sequences at chr7 and chr8. However, according to th
sequences were found at chr7 and chr8.

Table s6, Did you conduct a busco evaluation of Genome annotation, and what were the results?
Response:
Yes, a BUSCO evaluation of genome annotation was added. Now it reads,
Furthermore, BUSCO analysis showed that the annotated genes exhibit 97.3% completeness in the database (Supplementary

Line 146, Only the assembly software version is written here, but the name is not written?
Response:
Sorry for the mistake. Thanks for pointing it out. Now it reads,
The initial P. praeruptorum contig assembly was performed by hifiasm (version 0.19.5) with default parameters to obtain the

Line 195, Has the model been filtered before executing RaxML?
Response:
Thanks for the comments. Yes, it was filtered before executing RaxML. Now it reads,
Then, the corresponding protein sequences of single-copy supergene were filtered with a minimum amino acid length of 200,

Line 262, According to supply table s2, should the output of PacBio HiFi sequencing be 72.6Gb?
Response:
Sorry for the mistake. Now it reads,
Further, we applied PacBio HiFi sequencing (72.6 Gb), ONT Ultra-Long DNA sequencing (349.7 Gb), and Hi-C sequencing (53

Line 267, According to supply table s2, should the output of ONT Ultra-Long DNA sequencing data be 350Gb？
Response:
Sorry for the mistake. Now it reads,
Further, we applied PacBio HiFi sequencing (72.6 Gb), ONT Ultra-Long DNA sequencing (349.7 Gb), and Hi-C sequencing (53

Reviewer #2: General comments:
Peucedanum praeruptorum is a valuable Chinese medicinal plant used to treat coughs with thick sputum and dyspnea for a lo
descriptive. I have many concerns about this manuscript, especially regarding the T2T assembly process, subsequent bioinfor

Major comments:
1. The authors briefly described the T2T assembly process. As the T2T assembly is the main highlight of this manuscript, I su
gaps after Ultra-Long promotion. In Fig. 1c, the telomeres were marked, but I did not find any description of the identificatio
recommend that the authors compare the two assemblies and highlight the improvement of their assembly. "Genome annota
Response:
Thanks for the comments. "Genome assembly" and "genome annotation" were merged into one section as suggested by the
rewritten. Now it reads,
Further, we applied PacBio HiFi sequencing (72.6 Gb), ONT Ultra-Long DNA sequencing (349.7 Gb), and Hi-C sequencing (53
S2, Supplementary Data Table S3). A chromosome-level genome assembly (hereafter named 'chromosome genome') with Hi
chromosomes (Figure 1B and 1C; Supplementary Data Table S3 & S4). This nearly gapless genome consists of 253 contigs, N

The methods for identification of telomeres and centromeres were added, now it reads,
The ONT ultra-long reads were aligned to the 'chromosome genome' sequences using minimap2 (version 2-2.24) and facilitat
with the median extendable length were defined as ref and the others as query. Medaka_consensu (version 1.7.2, parameter
2.11.0+) according to the positional relationship in the alignment. The telomere sequence was replaced with the aligned sequ
and the LINE/L1 distribution is consistent with the centromere distribution of P. praeruptorum, and this region is also located

Also, we performed more analysis on the comparison with the newly published P. praeruptorum genome, the data from this s

2. Lines 323-324: The statement, "This is consistent with the previous hypothesis that the Apiaceae members experienced tw
accurate to say that P. praeruptorum experienced two rounds of WGDs rather than attributing it to the entire Apiaceae family
only one round of WGD
(https://apc01.safelinks.protection.outlook.com/?url=https%3A%2F%2Fwww.sciencedirect.com%2Fscience%2Farticle%2Fpii
FpbGZsb3d8eyJWIjoiMC4wLjAwMDAiLCJQIjoiV2luMzIiLCJBTiI6Ik1haWwiLCJXVCI6Mn0%3D%7C3000%7C%7C%7C&sdata=vI
Response:
Yes, we over stated the species that experienced two rounds of WGDs. The context was corrected as suggested by the review
Two major peaks were observed, which is consistent with the previous hypothesis that P. praeruptorum experienced two WG

3. I highly suggest the authors to at least hypothesize the categories of enzymes involved in the biosynthesis of Praeruptorin
pyrancoumarins, and these have been reported in multiple previous studies, which made the results tedious and lack of innov
Response:
Thanks for pointing this out. The biosynthesis pathway of Praeruptorin was revised as suggested by the reviewer, Figure 3 wa
P450s are a well-established enzyme superfamily present across various organisms. Their primary function involves catalyzin
contributors to phytochemical diversification and aiding in adaptation to fluctuating environmental conditions (Mizutani & Oht
PpDC) belong to CYP71 or CYP71-clan families are identified. In addition, the physical clustering of homologous P450s, often
Nelson & Werck-Reichhart, 2011). This clustering phenomenon has been previously identified in genes associated with furanc
pairs on chromosome 11 (Figure 3D). With the elucidation of PpOC that catalyzes the formation of lomatin, the enzyme that i
candidate enzymes for C-4′ skeleton hydroxylation and the following O-prenyltation step in this region. However, this hypoth

4. The authors did not pay enough attention to the language and the logical flow between paragraphs. Especially, the logic in
these contents or delete them. I have caught quite some improper usage of language and grammar mistakes. I highly require
Response:
Thanks for pointing this out. The organization, language and grammar of this manuscript was checked by a professional langu

Minor comments:
1. Line 62-63: "Several previous publications have shown that coevolution between plant and their predators is a matter of a
Response:
Sorry for the mistake. We revised the introduction and deleted these statement.

2. Lines 145-146: "v0.7" seems to be version information rather than software.
Response:
Sorry for the mistake. Thanks for pointing it out. Now it reads,
The initial P. praeruptorum contig assembly was performed by hifiasm (version 0.19.5) with default parameters to obtain the

3. Line 148: "The interaction ma generated by Juicer was manual checking and the visual refinement by Juicebox Assembly T
Response:
This part on genome assembly was rewritten. Now it reads,
Meanwhile, Blastn (version 2.11.0+, parameters: -evalue 0.00001 -max_hsps 1) was used to perform NT alignment on the d
redundant and contaminating sequences were removed, and finally generated a ‘chromosome genome’.

4. Lines 198-200: "two calibrated divergence time intervals: Oryza sativa - Vitis vinifera (163.5-142.1 Mya) and Panax notog
Response:
Thanks for pointing this out. The calibration time points come from the commonly used website https://timetree.org/. The co

5. Lines 215-216: "The clean reads were aligned to the newly assembled references genome and gene sequence using HISAT
Response:
Thanks. HISAT was used to align to the genome and Bowtie2 was used to align to the genes, respectively.

6. Lines 246-247: "The coumarin biosynthesis related cytochrome P450 gene family with CYP71A and CYP82C as query to bla
Response:
Thanks for the comments. Homology-based enzyme identification is with the assumption that closely related organisms share

enzymes to known enzymes in well-characterized organisms. With a more stringent similarity cutoff, we retrieved fewer gene
less than 70%, this may mistakenly include enzymes that may not have similar functions and increase the workload of future

7. Lines 262 and 267: The authors generated 726 Mb of data for PacBio HiFi sequencing and 350 Mb of data for ONT Ultra-Lo
Response:
Sorry for the mistake. Now it reads,
Further, we applied PacBio HiFi sequencing (72.6 Gb), ONT Ultra-Long DNA sequencing (349.7 Gb), and Hi-C sequencing (53

8. Lines 288-294: The same description has been stated in the "Methods" section; please simplify these statements. Addition
Response:
Sorry for the mistake. We used GlimmerHMM and AUGUSTUS for de novo annotation. The context was corrected as suggeste

9. Lines 311-312: Authors mentioned the performance of functional enrichment analysis. Could you please provide details on
Response:
Thanks for pointing this out. We have provided details on the findings of biological processes or functions associated with the
The genome of an organism is a dynamic landscape, shaped by evolutionary forces and environmental pressures. The expans
oxidoreductase activity, and iron ion binding respectively (Supplementary Figures S14), and in the KEGG pathways of photos
pathway, is a common approach induced by iron starvation and is thought to mobilize the recalcitrant iron pools. Interestingl
reactions, and efficiently manage iron acquisition in nature. In contrast, the top 2 contracted gene families were enriched in t
(Supplementary Figures S13).

10. Lines 326 and 327: "haploid state" sounds wired.
Response:
Thanks for pointing this out. The context was corrected as suggested by the reviewer. Now it reads,
D. carota habours nine chromosomes in a haploid.

11. Line 460: "Data Availability". Authors should release the genome assembly and annotation rather than just raw data.
Response:
The genome assemblies and gene annotations have been deposited at Figshare with the accession link : https://figshare.com

12. Line 345-363: This paragraph describes the biosynthesis of Praeruptorin. I suggest that some enzyme names should be s
Response:
Thanks for pointing this out. The full name of the genes were listed. Now it reads,
phenylalanine ammonia lyase (PAL), cinnamate 4-hydroxylase (C4H), cinnamate 3-hydroxylase (C3H), CoA O-methyltransfer
CoA O-methyltransferase (CCoAOMT), feruloyl-CoA 6'-hydroxylase (F6’H), glucose 6-phosphate (U-6-P), glucose 6-phosphate

13. Line 395: "AYP71AJ"? or AmCYP71AJ?
Response:
Thanks for pointing this out. The context was corrected as suggested by the reviewer.
