# Supplementary material for: The telomere-to-telomere (T2T) genome of Peucedanum praeruptorum Dunn provides insights into the genome evolution and coumarin biosynthesis
Source: Gigascience. 2024 Jun 5;13:giae025. doi: 10.1093/gigascience/giae025 (PMC11152176; doi:10.1093/gigascience/giae025)
Supplement: giae025_Response_to_Reviewer_Comments_Revision_1 [file giae025_response_to_reviewer_comments_revision_1.pdf]

Author's Response To Reviewer Comments

All the comments and suggestions from the reviewer was incoorporated. Also the full manuscript was rewise for the english langaure to provide a better flow in the sentenses through out.
We are gratefull for all the comments by the reviewers.

Yours truly,
Henrik Toft Simonsen
