# Supplementary material for: The telomere-to-telomere (T2T) genome of Peucedanum praeruptorum Dunn provides insights into the genome evolution and coumarin biosynthesis
Source: Gigascience. 2024 Jun 5;13:giae025. doi: 10.1093/gigascience/giae025 (PMC11152176; doi:10.1093/gigascience/giae025)
Supplement: giae025_Reviewer_1_Report_Original_Submission — Wei Sun -- 11/27/2023 [file giae025_reviewer_1_report_original_submission.pdf]

Reviewer Report

Title: The telomere-to-telomere (T2T) genome of Peucedanum praeruptorum Dunn provides insights into the genome evolution and coumarin biosynthesis

Version: Original Submission **Date:** 11/27/2023

Reviewer name: Wei Sun

Reviewer Comments to Author:

This manuscript completed the assembly and annotation work of the T2T level genome of Peucedanum praeruptorum, and conducted gene family and phylogenetic genomics analysis. The final genome assembly was approximately 1.798 Gb, assigned to 11 chromosomes, and genome completeness > 98%. This high-quality genome was obtained which provides molecular insights into the chromosomal distribution of the coumarin biosynthetic genes and serves as an important resource for designing engineering strategies for improving the production of these valuable compounds. However, I believe that this manuscript is lacking in terms of workload. I suggest that the author conduct a major revision and supplement the validation work of some structural genes or transcription factors. It is also acceptable to add some bioinformatics analysis work if possible. In addition, the following content in the manuscript needs to be modified and supplemented:
Some software does not indicate version, please verify the entire manuscript.
Why does the phylogenetic analysis of the P450 family only focus on the CYP71 and CYP82? Can the phylogenetic analysis of the P450 family be added?
Please provide the accession number for genome assembly and annotation.
The Genome assembly does not describe how the HiFi+Hi-C+Ultra long (365 contig) version of the genome was optimized to the T2T (253contig) version.
Adding non-coding RNA annotation method.
Adding the Telomere sequence identification method.
Table s3, Please provide statistical information on the HiFi and HiFi+HiC versions of the genome.
Table s5, Unexplained why the Telomere sequence was not detected at chr7 and chr8.
Table s6, Did you conduct a busco evaluation of Genome annotation, and what were the results?
Line 146, Only the assembly software version is written here, but the name is not written?
Line 195, Has the model been filtered before executing RaxML?
Line 262, According to supply table s2, should the output of PacBio HiFi sequencing be 72.6Gb?
Line 267 , According to supply table s2, should the output of ONT Ultra-Long DNA sequencing data be 350Gbï¼Ÿ

Methods

Are the methods appropriate to the aims of the study, are they well described, and are necessary controls included? Choose an item.

Conclusions

Are the conclusions adequately supported by the data shown? Choose an item.

Reporting Standards

Does the manuscript adhere to the journal's guidelines on minimum standards of reporting? Choose an item.

Choose an item.

Statistics

Are you able to assess all statistics in the manuscript, including the appropriateness of statistical tests used? Choose an item.

Quality of Written English

Please indicate the quality of language in the manuscript: Choose an item.

Declaration of Competing Interests

Please complete a declaration of competing interests, considering the following questions:

- Have you in the past five years received reimbursements, fees, funding, or salary from an organisation that may in any way gain or lose financially from the publication of this manuscript, either now or in the future?
- Do you hold any stocks or shares in an organisation that may in any way gain or lose financially from the publication of this manuscript, either now or in the future?
- Do you hold or are you currently applying for any patents relating to the content of the manuscript?
- Have you received reimbursements, fees, funding, or salary from an organization that holds or has applied for patents relating to the content of the manuscript?
- Do you have any other financial competing interests?
- Do you have any non-financial competing interests in relation to this paper?

If you can answer no to all of the above, write 'I declare that I have no competing interests' below. If your reply is yes to any, please give details below.

I declare that I have no competing interests

I agree to the open peer review policy of the journal. I understand that my name will be included on my report to the authors and, if the manuscript is accepted for publication, my named report including any attachments I upload will be posted on the website along with the authors' responses. I agree for my report to be made available under an Open Access Creative Commons CC-BY license (http://creativecommons.org/licenses/by/4.0/). I understand that any comments which I do not wish to

be included in my named report can be included as confidential comments to the editors, which will not be published.

Choose an item.

To further support our reviewers, we have joined with Publons, where you can gain additional credit to further highlight your hard work (see: https://publons.com/journal/530/gigascience). On publication of this paper, your review will be automatically added to Publons, you can then choose whether or not to claim your Publons credit. I understand this statement.

Yes Choose an item.
