# Supplementary material for: The telomere-to-telomere (T2T) genome of Peucedanum praeruptorum Dunn provides insights into the genome evolution and coumarin biosynthesis
Source: Gigascience. 2024 Jun 5;13:giae025. doi: 10.1093/gigascience/giae025 (PMC11152176; doi:10.1093/gigascience/giae025)
Supplement: giae025_Reviewer_2_Report_Original_Submission — Li Wang -- 11/28/2023 [file giae025_reviewer_2_report_original_submission.pdf]

Reviewer Report

Title: The telomere-to-telomere (T2T) genome of Peucedanum praeruptorum Dunn provides insights into the genome evolution and coumarin biosynthesis

Version: Original Submission **Date:** 11/28/2023

Reviewer name: Li Wang

Reviewer Comments to Author:

General comments:
Peucedanum praeruptorum is a valuable Chinese medicinal plant used to treat coughs with thick sputum and dyspnea for a long time. Here, Bai et al. reported a T2T genome assembly for P. praeruptorum, which serves as an important resource for future breeding and synthetic biology applications. That being said, the findings from this manuscript are a little bit descriptive. I have many concerns about this manuscript, especially regarding the T2T assembly process, subsequent bioinformatic analysis, and the writing. I believe the manuscript needs substantial revision before it can be published.
Major comments:
1. The authors briefly described the T2T assembly process. As the T2T assembly is the main highlight of this manuscript, I suggest the authors provide a detailed explanation of the process. For instance, they could elaborate on the number of gaps after the HiFi data assembly, how the Ultra-Long data helped close these gaps, and how they filled in the remaining gaps after Ultra-Long promotion. In Fig. 1c, the telomeres were marked, but I did not find any description of the identification of telomeres and centromeres in either the "Methods" or "Results" sections, which are important for quality control of T2T assembly. Additionally, I observed that a genome assembly for P. praeruptorum has already been published. I recommend that the authors compare the two assemblies and highlight the improvement of their assembly. "Genome annotation" is quite descriptive. To make the "Results" section more compact, I suggest merging "genome assembly" and "genome annotation".
2. Lines 323-324: The statement, "This is consistent with the previous hypothesis that the Apiaceae members experienced two WGD events", is inaccurate. In Fig. 2a, the WGD (before 62.5 mya) is shared in Apiales, and the WGD (before 25.7 mya) is shared in Apioideae. Without considering the older WGD (e.g., the WGT shared by core eudicots), it would be more accurate to say that P. praeruptorum experienced two rounds of WGDs rather than attributing it to the entire Apiaceae family. In this study, the authors only sampled Apioideae species within Apiaceae, so the statement "Apiaceae members experienced two WGD events" is entirely incorrect. In the Mackinlayoideae subfamily, species like Centella asiatica experience only one round of WGD (https://www.sciencedirect.com/science/article/pii/S0888754321001920?via%3Dihub). Hence, the author should carefully and substantively review the relevant statements.
3. I highly suggest the authors to at least hypothesize the categories of enzymes involved in the biosynthesis of Praeruptorin and provide a list of potentially candidate genes based on their expressional profile among tissues and phylogenetic reconstructions. The genes introduced in the current version are all from the upstream biosynthesis pathway of pyrancoumarins, and these have

been reported in multiple previous studies, which made the results tedious and lack of innovation.
4. The authors did not pay enough attention to the language and the logical flow between paragraphs. Especially, the logic in the introduction is difficult to follow. Please do consider re-organizing paragraphs in the introduction section. The co-evolution between insect/ predators and plants is not relevant to the main results. Please do consider to largely shorten these contents or delete them. I have caught quite some improper usage of language and grammar mistakes. I highly require the authors to carefully edit the languages.

Minor comments:

1. Line 62-63: "Several previous publications have shown that coevolution between plant and their predators is a matter of arms race" needs references.
2. Lines 145-146: "v0.7" seems to be version information rather than software.
3. Line 148: "The interaction ma generated by Juicer was manual checking and the visual refinement by Juicebox Assembly Tools". What is "ma"? Do the authors mean "map"?
4. Lines 198-200: "two calibrated divergence time intervals: Oryza sativa - Vitis vinifera (163.5-142.1 Mya) and Panax notoginseng - Daucus carota (69.0-54.3 Mya)". When estimating the divergence time, authors should provide references for the calibration time points.
5. Lines 215-216: "The clean reads were aligned to the newly assembled references genome and gene sequence using HISAT v2.1.0 [33] and Bowtie2 [44], respectively". Why did the authors use two mapping algorithm? The explanation is needed.
6. Lines 246-247: "The coumarin biosynthesis related cytochrome P450 gene family with CYP71A and CYP82C as query to blast the P. praeruptorum genomes with the similarity of 70% as cutoff". Why did authors use "70%" as cutoff? Please provide the explanation.
7. Lines 262 and 267: The authors generated 726 Mb of data for PacBio HiFi sequencing and 350 Mb of data for ONT Ultra-Long sequencing. It seems odd! The 726 Mb HiFi data represent only 0.4x depth (726 Mb / 1.8Gb), and the 350 Mb ONT data represent only 0.2x depth. Did the authors determine that this data was sufficient to assemble a gap-free genome?
8. Lines 288-294: The same description has been stated in the "Methods" section; please simplify these statements. Additionally, in the "Methods" section, the authors claimed AUGUSTUS and SNAP were used for de novo prediction; however, in the "Results" section, the methods shifted to GlimmerHMM and AUGUSTUS. This is confusing.
9. Lines 311-312: Authors mentioned the performance of functional enrichment analysis. Could you please provide details on the findings? Specifically, what biological processes or functions are associated with the expanded and contracted genes identified in the study?
10. Lines 326 and 327: "haploid state" sounds wired.
11. Line 460: "Data Availability". Authors should release the genome assembly and annotation rather than just raw data.
12. Line 345-363: This paragraph describes the biosynthesis of Praeruptorin. I suggest that some enzyme names should be spelled in full to make it easier for unfamiliar readers to understand. For example, PAL, C4H, 4CL...
13. Line 395: "AYP71AJ"? or AmCYP71AJ?

Methods

Are the methods appropriate to the aims of the study, are they well described, and are necessary controls included? Choose an item.

Conclusions

Are the conclusions adequately supported by the data shown? Choose an item.

Reporting Standards

Does the manuscript adhere to the journal's guidelines on [minimum standards of reporting?]() Choose an item.

Choose an item.

Statistics

Are you able to assess all statistics in the manuscript, including the appropriateness of statistical tests used? Choose an item.

Quality of Written English

Please indicate the quality of language in the manuscript: Choose an item.

Declaration of Competing Interests

Please complete a declaration of competing interests, considering the following questions:

- Have you in the past five years received reimbursements, fees, funding, or salary from an organisation that may in any way gain or lose financially from the publication of this manuscript, either now or in the future?
- Do you hold any stocks or shares in an organisation that may in any way gain or lose financially from the publication of this manuscript, either now or in the future?
- Do you hold or are you currently applying for any patents relating to the content of the manuscript?
- Have you received reimbursements, fees, funding, or salary from an organization that holds or has applied for patents relating to the content of the manuscript?
- Do you have any other financial competing interests?
- Do you have any non-financial competing interests in relation to this paper?

If you can answer no to all of the above, write 'I declare that I have no competing interests' below. If your reply is yes to any, please give details below.

I declare that I have no competing interests

I agree to the open peer review policy of the journal. I understand that my name will be included on my report to the authors and, if the manuscript is accepted for publication, my named report including any attachments I upload will be posted on the website along with the authors' responses. I agree for my report to be made available under an Open Access Creative Commons CC-BY license (http://creativecommons.org/licenses/by/4.0/). I understand that any comments which I do not wish to be included in my named report can be included as confidential comments to the editors, which will not be published.

Choose an item.

To further support our reviewers, we have joined with Publons, where you can gain additional credit to further highlight your hard work (see: https://publons.com/journal/530/gigascience). On publication of this paper, your review will be automatically added to Publons, you can then choose whether or not to claim your Publons credit. I understand this statement.

Yes Choose an item.
